# Surface‐Engineered 2D Nanomaterials in Gas Sensors: Advancement and Challenges

**DOI:** 10.1002/smll.202410360

**Published:** 2025-07-04

**Authors:** Radha Bhardwaj, Martin Pumera

**Affiliations:** ^1^ Future Energy and Innovation Laboratory Central European Institute of Technology Brno University of Technology Purkynova 123 Brno 61200 Czech Republic; ^2^ Advanced Nanorobots & Multiscale Robotics Laboratory Faculty of Electrical Engineering and Computer Science VSB – Technical University of Ostrava 17. listopadu 2172/15 Ostrava 70800 Czech Republic

**Keywords:** 2D materials, gas sensing

## Abstract

2D nanomaterials like transition metal dichalcogenides (TMDs), MXene, nitrides, and black phosphorus‐based gas sensors have garnered extensive attention in recent decades. The extra ordinary physicochemical and electrical properties of 2D nanomaterials make them highly sensitive toward gas molecules at room temperature. However, despite their potential, the current gas sensing technology suffers from inadequate selectivity, inaccurate detection and environmental instability. This review provides an overview of recent developments in surface‐engineering routes to improve the sensing properties of 2D nanomaterials‐based gas sensors. First, it covers emerging 2D nanomaterials, their synthesis routes, and gas‐sensing mechanisms. Later on, thoroughly explores renowned surface‐engineering strategies such as defect modulation, nanoparticle functionalization, and heteroatom doping to enhance the gas sensing performance. Metal intercalation and partial surface oxidation/reduction approaches are also discussed to tune the sensing characteristics. Furthermore, single‐atom catalyst engineering highlights the anchoring of metal atoms on 2D nanomaterials to achieve enhanced atom utilization, leading to better catalytic sensing activities. The engineering techniques introduce effective surface sensitization, modulated carrier concentration in 2D materials. This review outlines the key objectives of surface‐engineering strategies to overcome the limitations of hybrid materials and pave the way for next‐generation sensors with enhanced sensing performance to impact a wide range of applications.

## Graphical abstract

This review article gives a detailed overview of the surface‐engineered two‐dimensional (2D) nanomaterials gas sensors, highlighting the potential of various surface engineering techniques for the design of advanced and smart gas sensors. This article covers synthesis techniques, properties and recent developments and challenges in the designing of surface‐engineered 2D nanomaterials‐based gas sensors for a variety of applications.

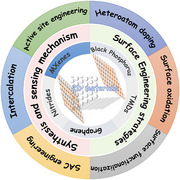

## 1 Introduction

Since the discovery and later commercialization of gas sensors owing to their ability to detect gas analytes at ultra‐low concentrations, dedicated to unveiling their sensing material engineering and different fabrication techniques, which have attracted much attention from academia and the industry. Amongst these, the sensing material is the core that defines the performance ability of a gas‐sensing device. Till now, a wide range of gas sensing materials such as metal oxides,^[^ ^1^, ^2^, ^3^, ^4^ ^]^ polymers,^[^ ^5^ ^]^ and 2D nanomaterials,^[^ ^6^, ^7^, ^8^, ^9^, ^10^, ^11^ ^]^ have all been explored. Primitive metal oxides marked their superior position by being the first gas‐sensing material in the 1960s.^[^ ^12^ ^]^ Many research and review articles have discussed gas‐sensing properties and modification engineering of metal oxides.^[^ ^1^, ^2^, ^3^, ^4^, ^12^, ^13^, ^14^ ^]^ Despite the huge implications of metal oxide sensors, their unresolved issues, like high operating temperature and poor selectivity, demand new‐age sensing materials to develop next‐generation gas detectors.^[^ ^13^, ^14^ ^]^ In the early 2000s, 2D nanomaterials were introduced in gas‐sensing technology to improve the 4S features of sensor performance: selectivity, sensitivity, speed, and stability.

First, in 2007, Schedin et al. fabricated the first 2D nanomaterial (graphene) based gas sensor and demonstrated that a single gas molecule can change the channel resistance by altering the local charge carrier concentration.^[^ ^15^ ^]^ This breakthrough research on semiconducting thin film sparked curiosity in using 2D materials as a gas‐sensing layer. Now, scientists have begun to turn to these other two‐dimensional crystals like TMDs,^[^ ^7^, ^8^, ^16^, ^17^, ^18^ ^]^ black phosphorus,^[^ ^19^, ^20^, ^21^ ^]^ 2D nitrides,^[^ ^10^, ^22^, ^23^ ^]^ and MXene,^[^ ^9^, ^24^, ^25^, ^26^ ^]^ have been potentially grown for gas sensing applications. The 2D nanomaterials show tremendous advantages in gas sensing owing to their atomically thick layered structure, intrinsic large specific surface area, and outstanding electrochemical properties.^[^ ^7^, ^27^, ^28^ ^]^ Unlike conventional semiconductors, 2D materials rely on direct charge transfer during gaseous interaction, facilitating the room temperature sensing abilities.^[^ ^16^, ^27^, ^28^ ^]^ In these two decades, other 2D materials excluding graphene became more mature and their synthesis techniques such as mechanical exfoliation,^[^ ^29^ ^]^ chemical exfoliation,^[^ ^24^, ^25^, ^26^ ^]^ electrochemical exfoliation,^[^ ^30^ ^]^ chemical vapor deposition (CVD),^[^ ^31^, ^32^, ^33^ ^]^ etc., advancements resulted in high purity atomically thin materials accelerating the practical implications in the gas sensor. 2D transition metal dichalcogenides (TMDs), as their name suggests, are made up of MX_2_ (M: Mo or W, X: S, Se, or Te) where transition metals such as Mo or W are linked with the chalcogens such as S and Se.^[^ ^8^, ^33^ ^]^ Unlike graphene, TMDs are not composed of a single layer of atoms; rather, they are a transition metal atom layer sandwiched between two layers of chalcogen atoms. TMDs are the second most explored 2D material in gas sensing, and have been exploited for the detection of gases such as NO_2_, NO, NH_3,_ and various volatile organic compounds (VOCs) at room temperature (RT).^[^ ^8^, ^16^, ^29^, ^34^ ^]^ Nevertheless, in 2017 MXene, a 2D carbide material with a formula M_n+1_X_n_T_x_, where M is a transition metal (like Ti, V, Cr, Zr, Nb, and Mo), X denoted as C or N and T represents surface terminal groups (like O, F, and OH) have been introduced in the gas sensing field.^[^ ^9^, ^35^, ^36^, ^37^ ^]^ Introducing MXenes in gas sensing brought a boom in the market by reporting its significance at the fastest pace, and became a topic of great interest. Chemically exfoliated 2D MXene sheets reported the detection of ultra‐low gas concentration with the lowest signal noise due to their metallic conductive nature.^[^ ^38^, ^39^, ^40^, ^41^ ^]^ The highly oxygen‐functionalized surface of MXene promotes the adsorption of gaseous molecules and exhibits excellent gas response to NH_3_, and NO_2_ to various VOC molecules.^[^ ^38^, ^39^ ^]^ In the 2D nitrides family, hexagonal boron nitride (hBN), also called “white graphene,” is a wide bandgap and the only existing insulating material in the 2D family.^[^ ^11^ ^]^ Chemically inert hBN is famous for gas sensing in harsh atmospheres and is reported for detecting various gases (NO_2_, NO, NH_3_, CO, CH_4_, H_2_, etc.).^[^ ^22^, ^42^, ^43^, ^44^, ^45^, ^46^ ^]^ Graphitic carbon nitride (g‐C_3_N_4_) nanomaterial possesses a huge specific surface area and reaction sites for gaseous interaction. The catalytic activity of g‐C_3_N_4_ under visible light illumination makes it highly attractive and engages researchers to work on efficient light‐assisted gas sensors.^[^ ^47^, ^48^, ^49^, ^50^, ^51^ ^]^ Lastly, black phosphorus (BP) is a promising 2D layered semiconductor material for next‐generation gas sensors that have high carrier mobility and thickness‐dependent electronic properties.^[^ ^52^, ^53^, ^54^ ^]^ BP is extensively studied as a selective gas sensing material owing to its natural selectivity toward the NO_2_ gas molecules. Remarkable research addressed highly sensitive, selective, and lower detection limits of BP‐based NO_2_ sensors.^[^ ^52^, ^53^, ^54^, ^55^, ^56^ ^]^ However, the purest form of 2D materials also shows some drawbacks in their sensing performance. For instance, the extremely sluggish response and huge noise in TMDs sensor,^[^ ^57^, ^58^, ^59^, ^60^ ^]^ high surface reactivity of MXene resulted in very unselective detection,^[^ ^61^, ^62^ ^]^ insufficient sensitivity of 2D TMDs at low detection limits, and so on, restricting the practical implementation of the gas sensors. Therefore, several surface and heterointerface engineering strategies have been adopted to overcome the aforementioned limitations of 2D materials. The surface engineering routes are more appropriate and can overcome the drawbacks of hybrid formation, such as poor uniformity and changing the inherent properties of materials. Conveniently, atomically thin 2D materials are favorable for surface treatment routes such as active sites modulation,^[^ ^8^, ^40^, ^41^, ^59^, ^60^, ^61^ ^]^ doping,^[^ ^22^, ^63^ ^]^ surface functionalization, single atom catalyst (SAC) engineering ^[^ ^16^, ^50^, ^51^, ^64^, ^65^ ^]^ and so on to modulate their structure, bandgap, and carrier mobility and enhance gas sensing performances. Each surface engineering technique has its own working mechanism and changes the material properties differently. A plethora of research has been published on 2D nanomaterials; however, they tend to focus on a specific 2D material and its progress in the gas sensing field.^[^ ^34^, ^35^, ^66^, ^67^, ^68^ ^]^ Hence, this review aims to build a bridge between all emerging 2D materials and give a comparative analysis of a surface engineering strategy for gas sensing applications. Consequently, a thorough and systematic review to understand the impact of surface engineering approaches on different 2D nanomaterials in the gas sensing field is extremely required. The main objective of this review article is to give insight into the advancement of modification techniques of 2D materials to address the existing limitations and achieve desirable sensing outcomes.

**Figure 1:**
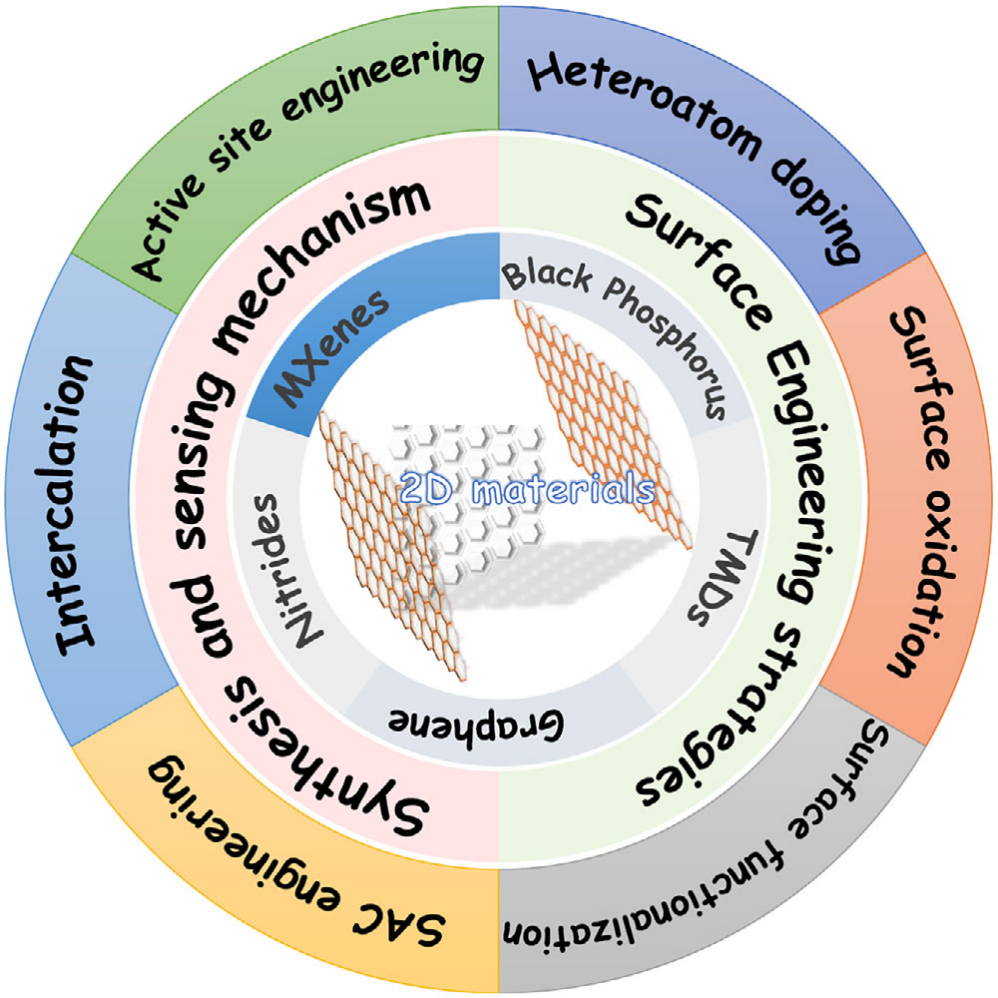
An overview of 2D materials and their surface engineering strategies, including synthesis and sensing mechanisms of the 2D materials.

Herein, we present a progressive review article that discusses state‐of‐the‐art surface engineering in 2D materials (excluding graphene), as shown in **Figure** **1**. We outline the different types of 2D materials and the recent trends of surface engineering by various modification routes. We explain how surface engineering routes modulate the chemical and electronic properties of 2D materials without hampering their crystal structure. We shed light on the sensing performance improvement in a surface‐treated 2D material‐based device. The review article highlights the challenges and future opportunities in developing 2D material‐based surface‐engineered gas sensing platforms. This review article discusses detailed dynamic evolution in different 2D materials under surface modification to conveniently achieve excellent sensing outcomes. Finally, the review article aims to advance research and innovation while laying the groundwork for upcoming developments in surface‐engineered 2D material‐based gas sensors.

## 2 Synthesis of Emerging 2D Nanomaterials

Layered materials with stacked sheets can be exfoliated from bulk to single‐layered material. In many layered materials, sheets are bonded with weak van der Waals forces that can be exfoliated by chemical and mechanical routes. The exfoliation of these sheets and the number of layers can be controlled and depend on the exfoliation technique. The synthesis of most 2D materials is classified under the top‐down and bottom‐up approaches. In the top‐down technique, 2D material is produced from the exfoliation of bulk layered solids, whereas bottom‐up methods involve the production of material from the atomic or molecular precursors. Single‐layered 2D materials are marked as most appropriate for sensing, where all surface atoms participate in the gaseous adsorption and have the largest surface area per unit volume.

**Figure 2:**
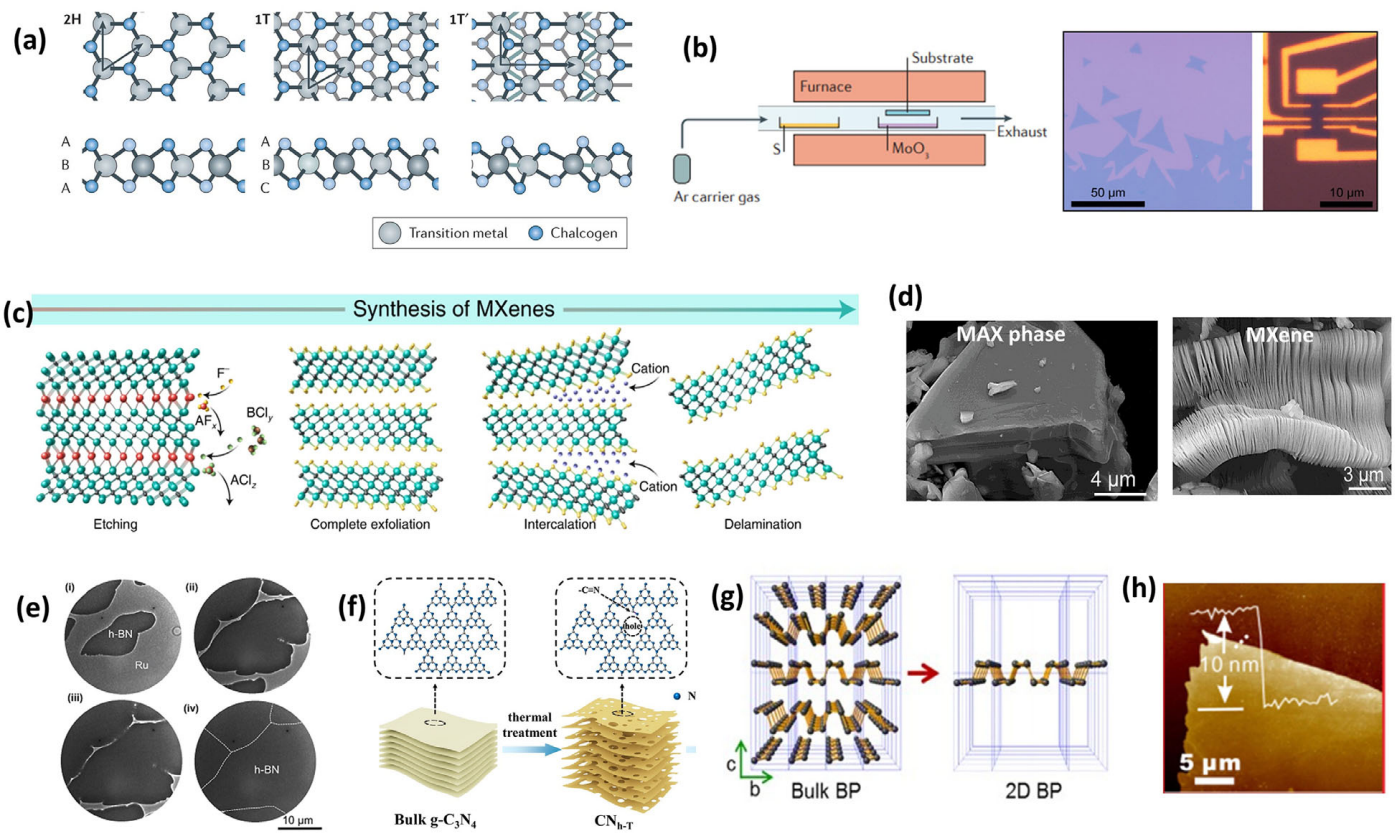
Example of 2D nanomaterials for gas sensing. a) Atomic structure of monolayer transition metal dichalcogenides (TMDCs) in their trigonal prismatic (2H), distorted octahedral (1T), and dimerized (1Tʹ) phases. Reproduced with permission.^[^ ^71^ ^]^ Copyright 2017, Springer. b) Optical image of CVD‐grown single MoS_2_ sheet on a silicon substrate. Reproduced with permission.^[^ ^33^ ^]^ Copyright 2014, American Chemical Society. c) Schematic of steps involved in the synthesis of MXene sheets. Reproduced with permission.^[^ ^37^ ^]^ Copyright 2022, Springer. d) SEM micrographs of Ti_3_AlC_2_ MAX phases and Ti_3_AlC_2_ MXene after HF treatment. Reproduced with permission.^[^ ^79^ ^]^ Copyright 2012, American Chemical Society. e) Low energy electron microscopy (LEEM) images i–iv) of CVD‐grown monolayer hBN domains on Ru(0001) from borazine at different elapsed times, such as i) 0, ii) 600, iii) 900, iv) 1350 s (complete coalescence). Reproduced with permission.^[^ ^80^ ^]^ Copyright 2011, American Chemical Society. f) Thermal condensation synthesis of g‐C_3_N_4_ nanosheets from bulk powder. Reproduced with permission.^[^ ^10^ ^]^ Copyright 2021, Elsevier. g) Atomic structure of bulk BP cleaved into monolayer BP. Reproduced with permission.^[^ ^84^ ^]^ Copyright 2016, Springer. h) AFM image of CVD‐grown BP film of several nanometers in thickness. Reproduced with permission.^[^ ^86^ ^]^ Copyright 2020, Springer.

In 2004, Novoselov et. al. reported the route leading to the preparation of atomically thick single‐layer planar graphene sheets from highly oriented pyrolytic graphite (HOPG) by the scotch tape method.^[^ ^69^ ^]^ The monocrystalline thin film of 2D materials is highly stable in ambient conditions and can show ambipolar behavior. Since that time, 2D materials have attracted a lot of attention from scientists. 2D TMD materials exist in different structural phases, and **Figure** **2**a represents two common structural phases of TMDs characterized by either trigonal prismatic (2H) or octahedral (1T) coordination of metal atoms. A variety of methods, such as arc discharge techniques,^[^ ^70^ ^]^ chemical and physical exfoliation, electrochemical exfoliation,^[^ ^30^ ^]^ and CVD^[^ ^29^, ^30^, ^31^, ^32^ ^]^ growth have been implemented to synthesize the TMDs nanomaterials. The bottom‐up CVD approach is mainly used to deposit the single‐ and few‐layer sheets of 2D TMDs that provide the largest surface area and are highly suitable for sensing applications. These methods to extract high‐purity 2D sheets are unfeasible for large‐scale solution‐based processes. Figure 2b represents the basic working principle of CVD growth of MoS_2_ on the substrate using MoO_3_ and S reaction at elevated temperatures.^[^ ^33^ ^]^ The SEM and optical images of the CVD‐grown monolayer MoS_2_ are shown in Figure 2b. Stacked sheets of 2D materials are more favorable for gas sensing due to various surface defects and functional groups.^[^ ^72^ ^]^ In this period, the development of discrete synthesis techniques resulted in high yield, purity, and economical production of 2D materials, which accelerated the commercialization of practical 2D materials applications. The mechanical exfoliation route proved to be of high quality and valuable for fundamental material properties.^[^ ^29^, ^32^ ^]^ However, significant challenges have been such as small flake size, limited uniformity, and lack of thickness control associated with the mechanical route.^[^ ^73^ ^]^ Gas sensing is thickness dependent, and single‐layer TMDs such as MoS_2_^[^ ^74^, ^75^ ^]^ and WS_2_^[^ ^76^ ^]^ with controlled layer thickness and uniformity were developed by the CVD method. Consequently, solution‐processed methods such as liquid‐phase exfoliation, dip coating, and the hydrothermal method have been widely adopted due to their ease of synthesis and processability.^[^ ^77^, ^78^ ^]^ 2D TMDs synthesized from these methods have been exploited in gas sensing and exhibited extraordinary gas‐sensing features at low‐power consumption to various gases.

MXene 2D sheets are most commonly synthesized from the chemical exfoliation of the A element from the M_n+1_AX_n_ phase by an etching process. The first MXene (Ti_3_C_2_T_x_) was synthesized by Gogotsi and his group members at Drexel University in 2011.^[^ ^36^ ^]^ Figure 2c includes all the steps involved in the 2D MXene synthesis.^[^ ^37^ ^]^ The top‐down synthesis approach of MXene involves a wide variety of etching routes, like hydrofluoric (HF) etching, electrochemical etching, molten salt solution, alkaline medium etching, and so on.^[^ ^35^, ^66^, ^79^ ^]^ HF was first used as an etchant for the exfoliation. Figure 2d shows the SEM micrograph of before and after etching with the MAX phase from HF etchant.^[^ ^79^ ^]^ MXene exfoliation is a bit different from other 2D materials, where mechanical exfoliation is not an effective route to break strong M–X bonds, but conveniently, the chemical route etched out layers of bulk Max phase.^[^ ^36^, ^79^ ^]^ The exfoliated MXene sheets have a huge number of functional sites (‐F, ‐Cl, ‐OH, and ‐O), which are extremely beneficial for gas‐sensing applications. Later, single or few‐layered MXene with a huge surface area can also be obtained by the intercalation and delamination of multilayered stacked MXene sheets.^[^ ^38^, ^39^ ^]^ Moreover, a few proposed agents like DMSO (dimethyl sulfoxide)^[^ ^38^ ^]^ and tetramethylammonium hydroxide (TMAOH)^[^ ^39^ ^]^ successfully produced the single‐layer MXene sheets.

The main synthesis methods of the remaining 2D materials, like nitrides (hBN) and BP, are mechanical exfoliation, CVD, liquid phase exfoliation, and pulsed laser deposition processes.^[^ ^68^, ^80^, ^81^, ^82^ ^]^ After graphene, the mechanical exfoliation route still maintains popularity for the synthesis of monolayer to a few layers of 2D materials.^[^ ^83^ ^]^ Nonetheless, liquid exfoliation appears to be an adverse approach to designing high‐quality 2D films.^[^ ^84^ ^]^ CVD is still a sophisticated route to synthesize high‐quality monolayers of 2D materials. Sutter et. al. reported CVD‐grown large‐area hBN monolayer from borazine on Ru(0001) single crystals and thin films.^[^ ^80^ ^]^ Figure 2e shows microscopic h‐BN domains on Ru(0001) from borazine at a range of lapsed times i) 0, ii) 600, iii) 900, and iv) 1350 s. The g‐C_3_N_4_ nanosheets are mainly synthesized by the thermal condensation of rich nitrogen source precursors such as melamine, dicyandiamide, and urea, to achieve a high specific surface area and more reactive sites in the material.^[^ ^10^, ^48^, ^49^, ^85^ ^]^ Further, bulk g‐C_3_N_4_ powder is thermally treated at high temperatures in an N_2_ atmosphere to inhibit the decomposition of g‐C_3_N_4_ in the air, as shown in Figure 2f.^[^ ^10^ ^]^ Figure 2g shows the schematic representation of bulk BP cleaved into monolayer BP after using the appropriate exfoliation route.^[^ ^84^ ^]^ Xu et. al. achieved a lateral epitaxial synthesis of high‐quality BP films with a modulated thickness from a few to hundreds of nanometers, as shown in Figure 2h.^[^ ^86^ ^]^ Some progress has been made in the existing techniques to improve the quality and stability of the synthesized 2D materials.^[^ ^35^, ^57^, ^66^, ^67^, ^68^, ^82^ ^]^ The gas sensing properties, such as the number of layers, specific surface area, reactive sites, and good adsorption performance of 2D materials, are dependent on the synthesis technique. The selection of an appropriate synthesis route can significantly improve the sensing properties of the pristine material.

The utilization of 2D materials in gas sensing is owing to their superior electrical, mechanical, and chemical properties. Notably, the properties of the above‐discussed 2D materials vary concerning the processing variables applied during the synthesis, atomic thickness, and modification strategies. The beneficial properties of these 2D materials, i.e., TMD, and carbides and nitrides, are the inherent atomically thin thickness, high surface‐to‐volume ratio, direct charge transfer, superior compatibility in ultra‐thin silicon channel technology, modulated bandgap, and high carrier mobility, which help in achieving exceptional gas sensing performances.^[^ ^35^, ^57^, ^66^, ^67^, ^68^, ^82^ ^]^ The bipolar charge carriers in an atom‐thick two‐dimensional structure and tunable charge density in the applied electrical field open big avenues in the modulation of gas‐sensing device performance. A high signal‐to‐noise ratio, which arises from the low intrinsic noise of 2D materials, is useful for signal stability and good chemical sensing performance. Tunable surface work function during gaseous interaction can be attributed to the changes in the surface carrier concentration of the sensing channel, independent of molecular diffusion into the bulk, showing a huge difference in sensing response.^[^ ^87^ ^]^ Defects are crucial in gas sensing, and defective 2D materials such as TMDs show a strong interaction toward gases such as CO, NO, or NO_2_ compared to the pristine material.^[^ ^88^ ^]^ Pure 2D materials are so promising for various applications, but sensing does not need high‐quality sheets because defective sites are favorable for gas adsorption. For this purpose, modified and engineered 2D materials are more advantageous and render functional groups and large amounts of defective sites.^[^ ^35^, ^57^, ^66^, ^67^, ^68^, ^82^ ^]^

## 3 Gas Sensing Mechanism of 2D Nanomaterials

**Figure 3:**
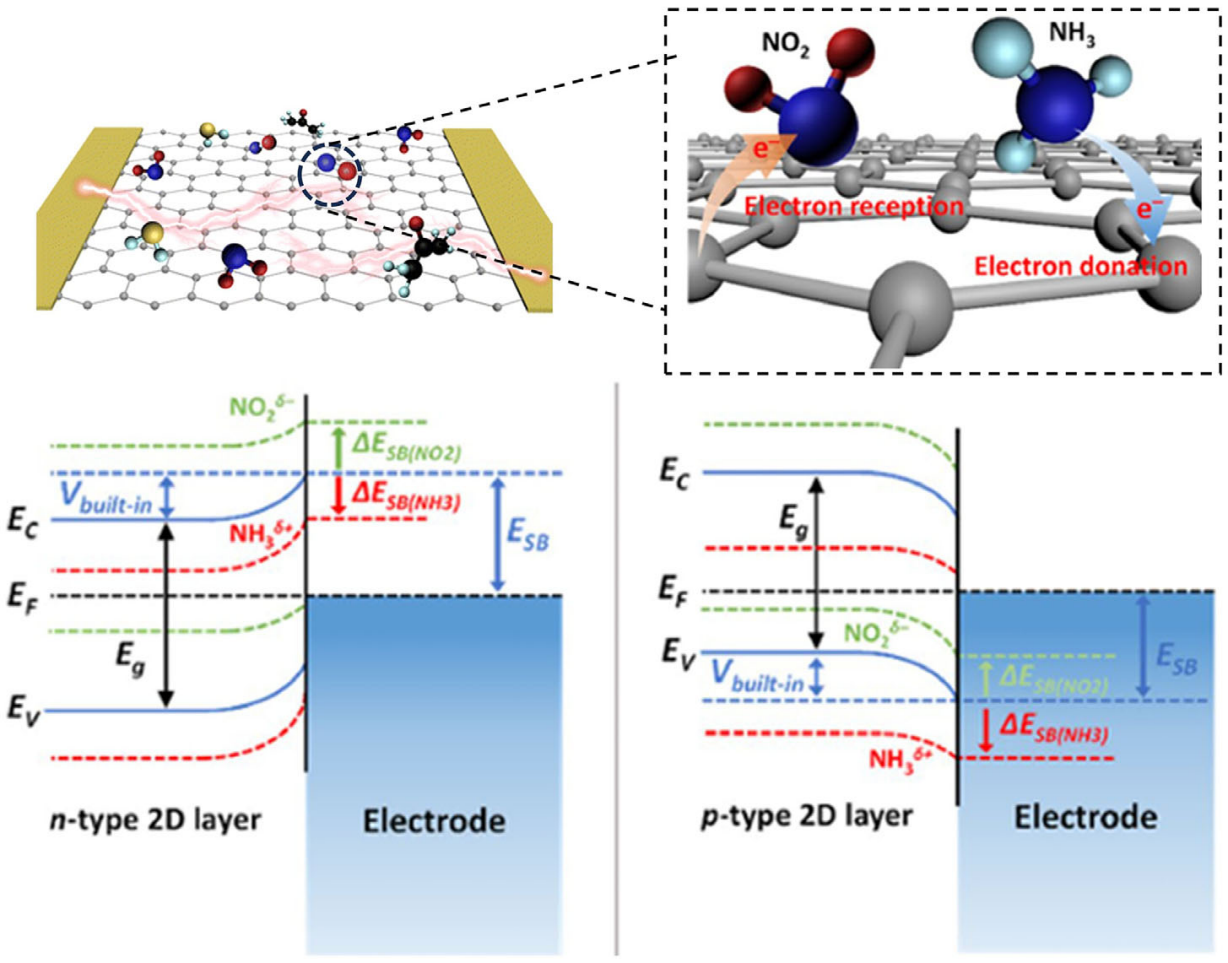
The gas sensing mechanism of 2D nanomaterials via surface charge transfer and Schottky barrier modulation. Reproduced with permission.^[^ ^89^ ^]^ Copyright 2018, Springer.

The sensing mechanism provides the theoretical platform for the design of new sensing technology.^[^ ^9^, ^16^, ^23^, ^25^ ^]^ The basic gas sensing mechanism involves the interaction of the gaseous analytes with the adsorbed oxygen species on the sensing layer, showing a change in the physico–chemical properties of the sensor.^[^ ^9^, ^16^ ^]^ From our perspective, the interaction of gaseous analytes on the sensing layer depends on the catalytic activities and adsorption sites. Consequently, the nanostructures and their modification strategies have a substantial impact on the sensing performance and determine the electronic structure of the semiconducting 2D channel.^[^ ^66^, ^89^, ^90^ ^]^ Computational chemistry is an important tool for identifying the sensing properties through density functional theory (DFT) calculations and molecular dynamics simulations to aid in choosing the appropriate sensing material.^[^ ^21^, ^26^, ^38^ ^]^ To date, scientists have proposed different fundamental gas sensing mechanisms of 2D nanomaterials, but still mostly unknown.^[^ ^66^ ^]^ The most established sensing mechanism of 2D thin materials is based on the physical or chemical adsorption, charge transfer phenomenon, and Schottky barrier modulation.^[^ ^9^, ^16^, ^39^, ^89^ ^]^ The primary mechanism of thin 2D nanomaterials involves the surface oxygen adsorption and desorption model, and is widely studied by researchers, as shown in **Figure** **3**.^[^ ^39^, ^55^ ^]^ In air ambient, oxygen species interact with the surface reactive sites of the material through physical or chemical adsorption and form anionic species such as O^2−^, and O^−^ by capturing the electrons from the conduction band.^[^ ^35^, ^55^ ^]^ The formation of different oxygen anions is directly dependent on the operating temperatures or under light illumination, as shown in Equations (1), (2), (3), (4).^[^ ^26^, ^35^, ^55^, ^91^ ^]^ In p‐type semiconducting material, the capturing of electrons from the conduction band decreases the baseline resistance. When the reducing target gas is introduced into the ambient, oxygen anionic species react with the gas molecules and release the electrons back to the conduction band of the material, showing an increase in resistance.^[^ ^35^, ^39^ ^]^ Take the NH_3_ gas as an example, where NH_3_ interacts with the sensing layer, resulting in more NH_4_^+^ reaction with the oxygen anions and narrowing the width of the hole accumulation region, consequently increasing the resistance of the sensing layer (Equation 5).^[^ ^26^, ^35^, ^65^ ^]^

(1) $$O_{2}\;\left(gas\right)\to O_{2}\left(adsorbed\right)\;$$

(2) $$O_{2}\left(gas\right)+e^{-}\to O_{2}^{-}\left(adsorbed\right)\left(T<100^{\circ }C\right)$$

(3) $$O_{2}^{-}\left(adsorbed\right)+e^{-}\to 2O^{-}\left(adsorbed\right)\left(100^{\circ }C<T<300^{\circ }C\right)$$

(4) $$O^{-}\left(adsorbed\right)+e^{-}\to O^{2-}\left(adsorbed\right)\left(T>300^{\circ }C\right)$$

(5) $$4NH_{3}\left(gas\right)+5O_{2}^{-}\left(adsorbed\right)\to 4NO+6H_{2}O+5e^{-}$$

Charge transfer is the main mechanism concept in 2D thin materials, where charge transfer occurs during the interaction of gas molecules with the surface reactive sites of the sensing channel, as shown in Figure 3.^[^ ^9^, ^25^, ^35^ ^]^ The direction and the amount of charge transfer are directly dependent on the type and chemical structure of the gases, resulting in distinct gas sensing magnitudes. In general, oxidizing gases (NO_2_ and CO) capture the free charge carrier from the sensing surface of 2D materials, while reducing gases (NH_3_, H_2_, and H_2_S) release the electrons to the sensing channel during the interaction.^[^ ^9^, ^16^, ^26^, ^35^, ^65^ ^]^ When the sensor is exposed to the gaseous atmosphere, the target gas acts as a temporary dopant in the sensing layer, contributing to the free charge carriers and changing the resistance value of the sensor.^[^ ^9^, ^26^ ^]^ On the contrary, desorption of the target gas from the sensing layer helps to restore the resistance value to the original state. Mostly, 2D materials show p‐type sensing responses where holes act as the majority carrier, resulting in a positive change in resistance toward the reducing gases.^[^ ^9^, ^16^, ^65^ ^]^ However, the type of conductivity in 2D materials can be easily modulated by surface engineering routes like doping and surface functionalization. Oxidizing gases acquire electrons from the p‐type material and increase the concentration of the majority free charge carriers, resulting in a high conductance value. The opposite phenomenon can be observed in the n‐type sensing materials.

Another important aspect of the sensing mechanism in 2D materials is the Schottky barrier modulation, where the interaction of gas analytes with the 2D sensing channel induces a change in the built‐in potential and Schottky barrier (Figure 3).^[^ ^9^, ^16^, ^23^, ^26^ ^]^ In brief, the pristine p‐type 2D material shows an accumulation of holes when oxidizing NO_2_ gas is adsorbed, resulting in increased conductivity due to the decrease in Schottky barrier and increased built‐in potential.^[^ ^9^, ^16^, ^89^ ^]^ The opposite behavior is detected under reducing NH_3_ gas by decreasing the conductivity of the p‐type sensing layer.^[^ ^26^, ^35^, ^65^ ^]^ In contrast, the n‐type sensing layer showed a shift of the Fermi level toward the valence band under NO_2_ influence. The surface engineering in 2D materials significantly alters the Schottky barrier and influences the sensing characteristics.^[^ ^19^, ^26^ ^]^ For instance, the functionalization of noble metals on 2D sheets resulted in the formation of metal‐semiconductor discrete Schottky junctions (M‐S).^[^ ^16^, ^50^, ^51^, ^64^, ^92^ ^]^ Metal semiconductor junctions such as CdSe‐ZnS QDs,^[^ ^16^ ^]^ Ag/G‐C_3_N_4_,^[^ ^50^ ^]^ ZnO/Ag/g‐C_3_N_4_,^[^ ^51^ ^]^ Au/SnS_2_,^[^ ^64^ ^]^ and Au, Pd, Pt NPs/MoS_2_^[^ ^65^ ^]^ have been reported in the literature for the efficient sensing of oxidizing or reducing gases. The work function difference between 2D materials formed a thicker depletion region and changed the conductivity properties of the material in the air atmosphere.^[^ ^16^, ^50^, ^64^, ^65^ ^]^ The change in conductivity after gas adsorption is significantly higher in metal‐functionalized materials compared to pristine materials due to the effective width of the depletion region and the built‐in potential.^[^ ^51^, ^92^ ^]^ The metal oxide functionalization in 2D materials leads to the formation of p–n or n–n or p–p heterojunctions, leading to modulation in effective charge carriers and stability of the material.^[^ ^9^, ^26^, ^55^, ^93^ ^]^ Zhao et. al. reported the NO_2_ gas sensing mechanism of p–p Mo_2_TiC_2_T_x_/MoS_2_ heterojunction sensor.^[^ ^9^ ^]^ The work function difference leads to a significant electronic charge transfer from Mo_2_TiC_2_Tx to MoS_2_, resulting in a wide depletion region at the heterointerface. The heterojunction modulates the intensity of charge transfer and synergistically enhances the gas sensing characteristics of the heterostructure.

## 4 Surface Engineering Strategies for 2D Nanomaterials

2D materials like TMDs, MXene, BP, and 2D nitrides are famous for their extraordinary gas‐sensing characteristics due to their ability to undergo various physicochemical reactions under various environmental conditions. Their 2D nature makes them ideal candidates for room temperature gas sensing and shows excellent sensing performance for a variety of gases like NH_3_, NO_2_, CO, and so on. However, the performance of these materials is lacking in their pristine state and requires slight modification in their ordinary properties to overcome the limitations of the materials. Thus, researchers are focusing on preserving the intrinsic properties of 2D materials but are also trying to modify the surface properties, which are responsible for the gaseous reactions. Therefore, electrochemical modifications through surface engineering routes are extensively explored in layered materials. Surface engineering of 2D materials allows the tuning of surface and chemical properties and benefits sensors to achieve higher gas selectivity, response, fast response/recovery, and higher environmental and long‐term stability. The characteristics of surface‐engineered 2D materials gas sensors are summarized in **Tables** **1** and **2**.

**Table 1:** Detailed gas sensing performance analysis of surface‐engineered TMDs and MXenes.

2D material	Engineering route	Synthesis procedure	Target gas	Concentration part per million [ppm]	Sensitivity	Op. tem. [°C]	Refs.
***TMDs***							
MoS_2_	Basal plane activation	Hydrothermal	NO_2_	500	213%	RT	[8]
MoS_2_	Sulfur vacancy	Liquid exfoliation	NO_2_	5	330%	100	[58]
SnS_2_	Defects and interlayer	Microwave	NO_2_	2	410%	RT	[59]
WS_2_	S‐defects	Ion intercalation	NH_3_	100	5%	25	[60]
MoS_2_	Mo and S defects	Alcohol‐assisted hydrothermal	humidity	11%	5.4 × 104%	RT	[94]
MoS_2_	Basal plane activation	Hydrothermal	NO_2_	100	1405%	RT	[95]
MoS_2_	Mo and S defects	Hydrothermal	NO_2_	50	38%	100	[96]
MoS_2_	Sulfur vacancy	Hydrothermal	NO_2_	0.2	226%	RT	[98]
MoS_2_	Plane defects	CVD	Naphthalene	–	–	RT	[99]
MoS_2_	Mo and S defects	Ion irradiation	NH_3_	200	340%	RT	[100]
WS_2_	Lattice defects	–	NH_3_	650	–	150	[101]
SnS_2_	Basal plane activation	Plasma treatment	NH_3_	20	5	125	[102]
WS_2_	S‐defects	Liquid exfoliation	H_2_S	–	–	187	[103]
MoS_2_	C‐doped	MOCVD	NO_2_	0.005	1.67	RT	[63]
MoSe_2_	O‐doped	Annealing	C_3_H_9_N	0.1	4	RT	[104]
MoS_2_	N‐doped	Plasma treatment	NO_2_	10	2.31	RT	[105]
MoS_2_	Zn‐doped	Hydrothermal	NH_3_	50	32.4%	200	[106]
MoS_2_	F‐doped	Hydrothermal	NO_2_	100	6.5	100	[107]
SnS_2_	C‐doped	Solvothermal	NO_2_	0.1	146.5%	RT	[108]
MoS_2_	V‐doped	APCVD	NH_3_	100	15.6	RT	[109]
MoSe_2_	N‐doped	Plasma treatment	NH_3_	25	430%	RT	[110]
SnS_2_	Ce‐doped	Solvothermal	NO_2_	0.5	1.67	100	[111]
WS_2_	Co, N‐doped	Pyrolysis	NO_2_	5	48.2%	RT	[112]
MoS_2_	Au‐doped	Exfoliation	NH_3_	500	150%	90	[113]
MoSe_2_	Ag‐doped	Hydrothermal	C_2_H_6_O	1	1.34	RT	[114]
SnS_2_	Co‐doped	Hydrothermal	NO_2_	0.001	89.7%	190	[115]
WS_2_	WO_3_	Thermal treatment	H_2_	1000	∼800%	150	[117]
SnSe_2_	SnO_2_	Thermal treatment	NO_2_	1	5.7	25	[118]
SnS	SnO_x_	Thermal treatment	NO_2_	0.001	171%	RT	[119]
SnSe_2_/SnSe	SnO_2_	Thermal treatment	NO_2_	5	256%	RT	[120]
WS_2_	WO_3_	Chemical treatment	NO_2_	5	16.7	25	[121]
MoS_2_	MoO_3_	Thermal treatment	H_2_	1000	25.5%	100	[122]
WS_2_	WO_3_	Thermal treatment	C_3_H_6_O	100	3.5	150	[123]
SnS_2_	SnO_2_	Thermal treatment	NO_2_	3	15.33	60	[124]
SnS_2_	SnO_2_	Thermal treatment	NH_3_	1000	92 000%	RT	[125]
SnSe_2_	SnO_2_	Thermal treatment	NO_2_	8	5.6	120	[126]
WS_2_	WO_3_	Laser induced	H_2_S	10	3.75	150	[127]
WS_2_	CdSe‐ZnS QDs	Dip coated	NO_2_	1	95.7%	RT+UV	[16]
SnS_2_	Au NPs	In situ reduction	NO_2_	50	∼15	RT+ UV	[64]
MoS_2_	Au, Pd, Pt NPs	Solution method	H_2_, NH_3_	–	1000% (H_2_)	RT	[65]
MoS_2_	Pt NPs	ALD	H_2_	1000	440	250	[128]
MoS_2_	Co NPs	Hydrothermal	NO_2_	100	546.6%	RT	[130]
WSe_2_	Pt NPs	Solution method	NO_2_	5	220%	RT	[131]
WS_2_	PtO and PdO NPs	Aerosol‐assisted CVD	NO_2_	0.8	26.5%	RT	[132]
MoSe_2_	Ag, Pd, Pt NPs	Solution method	NO_2_	32	2.42	110	[133]
MoS_2_	Au NPs	Hydrothermal	NO_2_	500	510%	50+LED	[134]
MoS_2_	Pd, g‐C_3_N_4_ NPs	Hydrothermal	TEA	30	9.85	225	[135]
SnS/WS_2_	Rh NPs	Hydrothermal	NO_2_	64	2.83	157	[136]
MoS_2_	Fe_2_O_3_	RF sputtering	SO_2_	5	32.2%	150	[137]
MoS_2_	PbS QDs	Organohot injection	NO_2_	10	615%	RT	[138]
WS_2_	Au NPs	Solution method	CO	50	∼4	RT	[139]
WS_2_	Pt NPs	ALD	NO_2_	10	842%	RT	[140]
WS_2_	PtS NPs	In situ chemical reduction	NH_3_	10	330%	RT	[141]
MoSe_2_	Pt NPs	Solution method	NO_2_	20	7.29	RT	[142]
SnS_2_	Au NPs	In situ reduction	NO_2_	5	14.2	RT +UV	[143]
WS_2_	Pt/Pd NPs	UV irradiation	C_3_H_6_O	50	∼4	20	[144]
SnSe_2_	Au/Pd NPs	Solution method	NO_2_	8	4.6	130	[145]
SnS/SnS_2_	Ce SAC	Hydrothermal	NO_2_	1	22.1	RT	[146]
MoS_2_	Pt, Co, and Ru	Solution method	C_2_H_6_O	50	–	RT	[147]
***Carbides***							
Mo_2_TiC_2_T_x_	MoS_2_ formation	In situ assembly	NO_2_	1	7.36%	RT	[9]
Mo_2_CT_x_	Sub‐µm MXene	HF + TBAOH etching	Humidity	4000	∼30%	RT	[40]
Nb_2_CT_x_	Exfoliated	HF+ CTAB	NO_2_	1	1.68	RT	[41]
Ti_3_C_2_T_x_	3D framework	electrospinning	CH_3_OH	–	2.2	RT	[61]
V_2_CT_x_	Interlayer expansion	HF + TBAOH etching	H_2_	100	0.0226	RT	[62]
Ti_2_CT_x_	Surface chemistry	HF + LiF/HCl etching	NH_3_	10	∼2%	RT	[151]
Mo_2_CT_x_	porous silicon hybrid	UV + phosphoric acid etching	CO_2_	50	1.14%	30	[153]
Ti_3_C_2_T_x_	Controlled etching	H_2_O + HCl/NaF (ultrasonic)	C_6_H_15_N	10	4%	23	[152]
Ti_3_C_2_T_x_	Termination modified	LiF + HCl	C_2_H_6_O	40	∼8%	RT	[154]
Ti_3_C_2_T_x_	Ti_3_C_2_(OH)_2_	LiF/HCl	C_3_H_6_O	100	0.97%	RT	[155]
Ti_3_C_2_T_x_	Metal‐ion intercalated	KCl, NaCl, CaCl_2_, and MgCl_2_	NH_3_ (Na‐MXene)	100	0.36%	RT	[157]
V_2_CT_x_	Surface chemistry	LiF+HCl etching	Methanol	500	3.41%	RT	[158]
Ti_3_C_2_T_x_	Na^+^ intercalation	LiF+HCl etchant + NaOH	C₂H₆O	0.1%	9.99%	RT	[159]
V_2_CT_x_	Alkalized MXene	HCl/NaF	NO_2_	50	57.6%	25	[161]
Ti_3_C_2_T_x_	Organ like	HF + Na ions	NH_3_	100	28.87%	RT	[162]
Mo_2_CT_x_	Thickness variation	TMAOH intercalation	VOCs (thin film)	5	0.615	RT	[164]
Ti_3_C_2_T_x_	N‐doped	Hydrothermal	NH_3_	0.2	7.3%	20	[165]
Ti_3_C_2_T_x_	N‐doped	Solvothermal reaction	CO_2_	8	∼1%	20	[166]
Ti_3_C_2_T_x_	S‐doped	Thermally treated	C_6_H_5_CH_3_	1	∼214%	RT	[167]
Ti_3_C_2_T_x_	TiO_2_	Oxygen plasma	C₂H₆O	100	22.47%	RT	[16]
Ti_3_C_2_T_x_	TiO_2_	In situ growth	NH_3_	30	40.6	RT	[26]
V_2_CT_x_	V_2_O_5_	Calcination	C_3_H_6_O	15	11.9%	RT	[169]
Ti_3_C_2_T_x_	TiO_2_	Annealing	C₂H₆O	250	–	20	[170]
Ti_3_C_2_	TiO_2_	Hydrothermal	NO_2_	5	∼1.13	RT	[172]
Ti_3_C_2_T_x_	TiO_2_	Hydrothermal	C_6_H_12_O	10	3.4%	RT	[173]
Ti_3_C_2_T_x_	TiO_2_	Solution mixing	NH_3_	10	3.1%	RT	[39]
Ti_3_C_2_T_x_	FOTS	Solution method	C_2_H_5_OH	120	14%	RT	[174]
Nb_2_CT_x_	(–NH_2_)	Hydrolysis reaction	NO_2_	25	31.52%	25	[175]
Ti_3_C_2_	Ag NPs	Utrasonication	Humidity	–	106.800%	RT	[176]
Ti_3_C_2_T_x_	Au‐ and Pt NPs	Solution method	NH_3_ (Au‐MXene)	100	∼16%	RT	[177]
Ti_3_C_2_T_x_	QDs	Solution method	NO_2_	0.5	60.38%,	RT	[178]
Ti_3_C_2_T_x_	Gelatin ink	Facile spray coating	NH_3_	50	7%	RT	[179]
Ti_3_C_2_T_x_	Pd nanoclusters	Alcohol reduction method	H_2_	4%	23%	RT	[180]
Ti_3_C_2_T_x_	Fe_2_(MoO_4_)_3_	Hydrothermal	C_4_H_9_OH	100	43.1	120	[181]
Ti_3_C_2_T_x_	TiOF_2_ nanospheres	Hydrothermal hydrolysis	Humidity	11% RH	–	RT	[182]
Ti_3_C_2_T_x_	Pt NPs	Solution method	NH_3_	10	22.7	RT	[183]
Ti_3_C_2_T_x_	Au NPs	Solution method	NH_3_	100	15.1%	RT	[184]
Ti_3_C_2_T_x_	V_2_O_5_ NPs	Hydrothermal	NH_3_	10	12.12	RT	[185]
Ti_3_C_2_T_x_	Pt SAC	Solution method	C_6_H_15_N	10	∼6.4%	RT	[188]
TiC_0.5_N_0.5_	Ni SAC	In situ doping	NO_2_	0.01	5%	RT	[190]
Ti_3_C_2_T_x_	Ag SAC	Solution method	NH_3_	10	4.35%	RT	[191]

**Table 2:** Detailed information on gas sensors based on surface‐engineered 2D nitrides and black phosphorus.

2D material	Engineering route	Synthesis procedure	Target gas	Concentration [ppm]	Sensitivity	Op. tem. [°C]	Refs.
***Nitrides***							
BN	Porous	Thermal treatment	H_2_S	89.3 µg mL^− 1^	–	245	[42]
BN	Memristor	–	NO	5	18%	RT	[43]
BN	Memristor	–	NO	–	8922	RT	[44]
BN	Fluorinated	Hydrothermal reaction	CO	–	–		[45]
BN	C‐doped	Pulse laser deposition	CH_4_	100	7.8	150	[22]
BN	Pyrene	Liquid‐phase exfoliation	Humidity	68% RH	–	20	[46]
BN	polyethylenimine layer	–	CO_2_	–	–		[213]
BN	BaF_2_	–	NH_3_	2.4 × 10^−2^	1	RT	[214]
BN	NiO NPs	Solvothermal method	C_6_H_15_N	500	23.75	300	[215]
g‐C_3_N_4_	Exfoliated sheets	Thermal oxidation	CO_2_	200	92%	300	[47]
g‐C_3_N_4_	Defective sheets	Ultrasonic dispersion	NO_2_	100	3.1 kHz	RT	[48]
g‐C_3_N_4_	Mesoporous	Ultrasonic dispersion	C_2_H_6_O	50	182.4	29.8	[49]
g‐C_3_N_4_	Surface defects	Ultrasonic dispersion	NO_2_	5	1470	RT	[196]
g‐C_3_N_4_	Exfoliated sheets	Thermal polycondensation	CO_2_	–	–	40	[197]
g‐C_3_N_4_	Fe/Mn	Evaporator	Xylene	100	25.4	370	[204]
g‐C_3_N_4_	C‐doped	Thermal polymerization	NO_2_	50	71.36%	200	[205]
g‐C_3_N_4_	S‐doped	Sintering	NO_2_	100	37.7%	RT	[206]
g‐C_3_N_4_	Nb‐doped	Pyrolysis	NH_3_	50	76%	RT	[208]
g‐C_3_N_4_	Sb‐doped	Hydrothermal	H_2_S	2	20.1	−20	[210]
g‐C_3_N_4_	Polyoxometalates	Electrospinning	C_3_H_6_O	100	10.53	330	[211]
g‐C_3_N_4_	Au NPs	Solution method	C_6_H_15_N	50	79	175	[23]
g‐C_3_N_4_	Ag NPs	Solution method	C_6_H_5_CH_3_	50	61.3%	200	[50]
g‐C_3_N_4_	Ag NPs	Homogenous mixing	NO_2_	5	15.3	RT	[51]
g‐C_3_N_4_	Pt NPs	Hydrothermal	H_2_	10 000	51	RT	[216]
g‐C_3_N_4_	CuO	Hydrothermal	DEA	–	–	RT	[217]
g‐C_3_N_4_	Au NPs	Solution method	CH_4_O	140	72.6%	65	[221]
g‐C_3_N_4_	Ag NPs	Solution method	C_6_H_5_CH_3_	50	42.97	100	[222]
g‐C_3_N_4_	Eu SA	Pyrolysis	VOC	–	–	RT	[223]
***BP***							
BP	Stacked flakes	Liquid exfoliation	Humidity	85% RH	–	RT	[20]
BP	Edge modification	Solution method	NO_2_	1	2358%	RT	[21]
BP	Monolayer	Scotch tape	NO_2_	0.005	–	RT	[33]
BP	Suspended	Mechanical exfoliation	NO_2_	200	23%	RT	[52]
BP	Defect enriched	Liquid exfoliation	NO_2_	0.1	88%	RT	[54]
BP	Defect enriched	Liquid exfoliation	NH_3_	100	121%	RT	[229]
BP	Microribbion	Thermal treatment	NO_2_	0.0004	8%	RT	[231]
BP	Layer dependent	Scotch tape	NO_2_	0.02	190%	27	[232]
BP	Few‐layered	Vapor transport growth	CH_4_O	1140	–	RT	[233]
BP	Zn‐doped	Hydrothermal	C_3_H_6_O	0.5	5.32	160	[239]
BP	Pt NPs	Solution method	NO_2_	32	1.95	50	[19]
BP	Co_3_O_4_ NPs	Hydrothermal	NO_x_	100	8.38	RT	[55]
BP	Ni NPs	Solution method	NO_2_	0.1	100%	RT	[56]
BP	benzyl viologen	Solution method	Humidity	20% RH	53.4%	RT	[240]
BP	Au, Pt NPs	In situ reduction	H_2_	1%	500%	RT	[242]
BP	Ag NPs	In situ reduction	NO_2_	0.1	39.9%	RT	[243]
BP	Dopamine	Self‐polymerization	NO_2_	1	4000%	RT	[244]
BP	TiO_2_ NPs	Hydrothermal	NH_3_	5	19.7	RT	[245]
BP	SnO QDs	–	NH_3_	10	3.3	25	[246]
BP	SnO NPs	Hydrothermal	H_2_S	5	233.8	130	[247]
BP	PIE/PEG	One‐pot method	CO_2_	200	5.8%	25	[248]

### 4.1 Transition Metal Dichalcogenides (TMDs)

TMDs are famous 2D materials involved in various applications and have gained wide attention from researchers in the gas sensing field. After graphene and its derivatives, TMDs are the most significant and preferred material due to their chemical, electrical, and surface properties. The properties of TMDs, such as low bandgap, good biocompatibility, high charge carrier mobility, strong immobilization ability, and tunable bandgap, give a huge rise to the material in the gas sensing field. However, TMD materials usage in gas sensing still exhibits challenges such as poor response, lower signal stability, long response time, and lower environmental and humidity stability in their pristine form. The challenges remain for the researchers to make it more applicable by just modulating the surface properties. Several cutting‐edge studies have been reported where researchers achieved excellent gas sensing characteristics from TMDs by modulating their properties through surface engineering routes, as summarized in Table 1.

#### 4.1.1 Active Site Engineering

TMDs, as a layered material, tend to exhibit defect modulation. Modulation in structural disorders or chalcogenide defects can significantly affect their physical and chemical properties. Morphological orientation of TMDs generally relies on the aggregation of sheets, resulting in flowered morphology.^[^ ^59^, ^94^, ^95^, ^96^ ^]^ The phrase “defect engineering” refers to the idea that materials can be engineered beyond well‐known concepts like doping or alloying and enable advanced properties.^[^ ^17^, ^97^ ^]^ It was evident that vertically oriented TMDs exhibited significantly higher edge functionality and chemical catalytic activity compared to planar‐oriented sensor devices.^[^ ^96^ ^]^ TMD materials have a widely observed defective structure regarding vacancies, adatoms, edges, basal plane grain boundaries, and substitutional atoms.^[^ ^58^, ^95^, ^98^ ^]^ These defects induce significant changes in the electronic, catalytic, and chemical properties of the TMDs.^[^ ^8^, ^59^, ^98^ ^]^ Sulfur or transition metal Vacancy enrichment leads to improved charge generation and transfer in the sensing channel, resulting in better‐sensing properties than pristine materials.^[^ ^58^, ^59^, ^98^ ^]^ Additionally, surface defects or vacancies in pure TMDs significantly increase the chemisorption sites for gas molecules.^[^ ^58^ ^]^ Different morphologies, layer separation, and defect generation in pure TMD material can be achieved by changing the synthesis conditions, such as precursor ratio,^[^ ^99^ ^]^ solvents,^[^ ^59^, ^94^ ^]^ and heat treatment.^[^ ^17^, ^58^ ^]^ Atomic vacancy defects in TMDs through proton irradiation in‐vacuo annealing and ion beam exposure are the most sophisticated techniques and provide a defect precision of up to 10 nm.^[^ ^18^, ^97^, ^100^ ^]^ Mitterreiter et. al. studied the effect of annealing temperature on the as‐exfoliated single‐layer MoS_2_ on hBN (green) supported on a Si/SiO_2_ substrate. Heating at mild annealing temperatures (T < 500 K, orange) removes the adsorbates from the surface, whereas high annealing temperatures (T > 500 K, red) create sulfur vacancies by thermal desorption. They claimed that defect emission intensity can be tuned by modulating annealing temperature and is a potential way for tailored defect engineering in TMDs.^[^ ^17^ ^]^ In the fully hBN‐encapsulated MoS_2_ layer, vacancy generation was achieved by He‐ion bombardment.^[^ ^17^ ^]^ Rao et. al examined the direction of charge transfer and the lattice defect generation kinetics in WS_2_ monolayer using in situ photoluminescence (PL) and resonance Raman spectroscopy.^[^ ^101^ ^]^ They concluded that shifts in the PL emission energies are correlated with the defect densities, such as sulfur vacancies, and an increase in the defects in the presence of NO_2_ and NH_3,_ showing a role of gases in the formation of lattice defects.^[^ ^101^ ^]^ Edge enrichment or highly oxygen‐functionalization offers massive unsaturated and dangling bonds on the sensing layer compared to a pure 2D material.^[^ ^96^ ^]^ In contrast, single‐layer MoS_2_ with modulated defect density shows better device performance and sensitivity.^[^ ^96^ ^]^ Inactive basal planes hinder the gas sensing activity of the 2D materials, and activation of these planes by any technique significantly alters the gas sensing output.^[^ ^95^, ^102^ ^]^ Sulfur defects assisted activation of the basal plane in MoS_2_ by Zn doping,^[^ ^95^ ^]^ and SnS_2_ by plasma treatment^[^ ^8^ ^]^ was done to dominate the physi‐ and chemisorption of NO_2_ and NH_3_ gases, respectively. Defect engineering is a powerful approach in sensing platforms to achieve higher selectivity and sensitivity toward a target analyte.^[^ ^8^ ^]^ Interestingly, the affinity of the analyte molecules strongly depends on the basal plane and structural defects in the lattice of the TMDs material.^[^ ^8^, ^60^ ^]^ Urbanos et. al. exploited the same concept and designed a MoS_2_ field effect transistor for sensing polycyclic aromatic hydrocarbons (PAHs), leveraging the attraction of PAHs toward the MoS_2_ basal plane and crystal defects. Naphthalene exposure showed a noticeable reduction in defectiveness and p‐type doping in MoS_2_.^[^ ^8^ ^]^ The magnitude was determined by the correlation between ionization energy, where naphthalene with the highest ionization energy showed the strongest output. Defect modulation in WS_2_ nanosheets was done by tuning in sulfur content to achieve high sensitivity and selectivity toward the target gases.^[^ ^60^, ^104^ ^]^ Moreover, defect modulation is a more attractive and convenient approach for tuning the electronic and surface properties to achieve improved sensing properties than pristine 2D TMD materials.^[^ ^100^, ^101^, ^102^, ^103^, ^104^ ^]^ Different research groups have successfully examined the enhanced NH_3_, NO_2_ gas sensing by defect‐engineered TMDs such as MoS_2_,^[^ ^8^, ^58^, ^94^, ^95^, ^98^, ^99^, ^100^ ^]^ WS_2_,^[^ ^60^, ^101^, ^103^ ^]^ and SnS_2_.^[^ ^59^, ^102^ ^]^

#### 4.1.2 Doping Engineering

**Figure 4:**
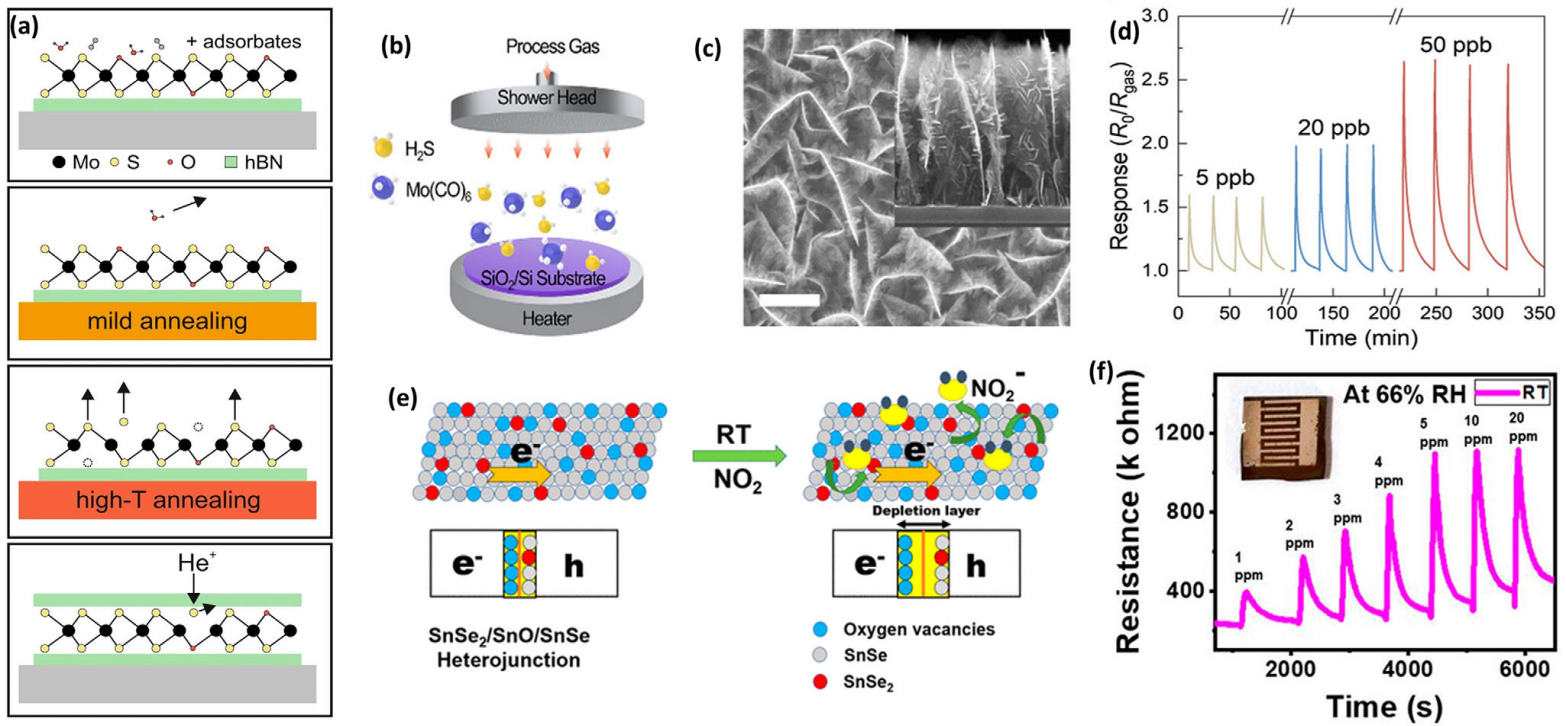
Defect and doping engineering in TMDs‐based gas sensors. a) Schematic of as‐exfoliated single‐MoS_2_, heating of substrate at mild and high different temperatures, and lastly vacancy generation through He‐ion (He^+^) bombardment supported by hBN (green) on a Si/SiO_2_ substrate (grey). Black (yellow) dots represent molybdenum (sulfur) atoms. Red dots denote oxygen atoms. Reproduced with permission.^[^ ^17^ ^]^ Copyright 2021, Springer. b) Schematic setup of metal‐organic CVD (MOCVD)‐based growth of C‐doped MoS_2_ nanobranches. c) SEM image of C‐MoS_2_ film grown for 240 min (height: 900 nm). d) Repeatability for lower NO_2_ concentrations of 5, 20, and 50 part per billion (ppb). Reproduced with permission.^[^ ^63^ ^]^ Copyright 2023, Wiley‐VCH. e) Diagram view of n−p−n transition in the SnSe_2_/SnO/ SnSe heterojunction mechanism at room temperature. f) Transient resistance curve of different NO_2_ concentrations at RT and 66% RH. Reproduced with permission.^[^ ^120^ ^]^ Copyright 2022, American Chemical Society.

Microstructural modifications using elemental doping have been well‐studied in TMD materials for various applications. Meanwhile, theoretical and experimental evidence proves that heteroatom doping or second‐phase incorporation is an efficient route to regulate the electronic structure, work function, and finally the chemical reactivity of the TMDs.^[^ ^104^, ^105^ ^]^ It is inferred that the addition of doping atoms like O,^[^ ^104^ ^]^ Zn,^[^ ^106^ ^]^ F,^[^ ^107^ ^]^ C,^[^ ^63^, ^108^ ^]^ V,^[^ ^109^ ^]^ and N,^[^ ^105^, ^110^ ^]^ into the crystal structure of the TMDs substantially influences the gas‐sensing properties. Doping of oxygen atoms is convenient, and only heating of pristine material at a mild temperature leads to a change in electronic structure and free charge carrier concentration.^[^ ^106^ ^]^ The size and electronegativity of the dopant atoms play crucial roles in the tailoring of interlayer attraction and lattice defects, and result in a huge accumulation of ionized oxygen species for the anchoring of gas molecules.^[^ ^105^, ^106^, ^111^ ^]^ Note that dopant size and amount of doping significantly modulate the electronic properties and interlayer spacing, which greatly affect the sensing parameters like sensitivity and selectivity of the pristine material.^[^ ^107^, ^111^ ^]^ High metallic conductivity in the material leads to a good signal‐to‐noise ratio, and signal stability in the gas sensor performance can be achieved by doping‐induced reduction of the Fermi level consequences in a smaller bandgap and high conductivity.^[^ ^105^ ^]^ Doping is also an adequate method for tuning the surface, chemical, and electrical properties without changing the microstructure of TMD materials.^[^ ^105^, ^106^, ^107^ ^]^ The doping in hierarchical nano branches enables more surface area and a large number of edge sites for the predominant gaseous interaction in TMDs.^[^ ^112^ ^]^ Vertically aligned TMDs exhibit higher adsorption energy toward the NO_2_ gas and unveil greater sensing capabilities than the horizontally aligned material.^[^ ^63^, ^112^ ^]^ Song et. al. reported large‐area carbon‐incorporating hierarchical MoS_2_ nano branches synthesized from MOCVD for efficient NO_2_ sensing as shown in **Figure** **4**b,c.^[^ ^63^ ^]^ C‐doped MoS_2_ nanobranches showed (part per trillion) ppt level NO_2_ detection with excellent repeatability at room temperature (Figure 4d). Interestingly, in this study, carbon is not only a dopant but also a seed layer for the growth of MoS_2_ branches and actively participates in superior electron transfer and high NO_2_ adsorption during the sensing process. In 2D materials, sometimes the sheet thickness is in a few‐nanometer range, and at that state, both surface doping and bulk doping are comparable and precisely controlled at the nanoscale.^[^ ^110^ ^]^ With precise dimensions of the material, the valence band is closer to the oxidation or reduction potential of the gases, which is favorable for the selective sensing of gases dependent on their electron‐accepting and donating nature.^[^ ^110^, ^112^ ^]^ The sheet dimensionality of TMDs was also tuned by using an attractive way to achieve maximum sensing output.^[^ ^112^ ^]^ Koo et. al. designed a system where few‐layered WS_2_ nanoplates (a lateral dimension of ≈10 nm) were confined in Co, N‐doped hollow carbon nanocages for highly sensitive NO_2_ gas sensing.^[^ ^112^ ^]^ During the pyrolysis process growth of WS_2_ is effectively suppressed, creating few‐layered WS_2_ nanoplates. The astonishing sensing results (48.2% response to 5 part per million (ppm) NO_2_) were attributed to the high gas permeability and reactivity in edge‐abundant confined WS_2_ in porous carbon cages assisted with MOF templates.^[^ ^112^ ^]^ Noble metals loading is also an effective strategy for enhanced gas sensing performance, but poor uniformity and overloading of metals drastically restrict the performance of gas sensors.^[^ ^113^, ^114^ ^]^ In note, noble metals like Au,^[^ ^113^ ^]^ and Ag,^[^ ^114^ ^]^ doping in pure TMDs like MoS_2_ and MoSe_2_ can overcome the limitation of noble metal loading and show excellent gas sensing behavior. Burman et. al. reported a comparative analysis where the Au‐doped MoS_2_ sensor exhibited a large response against NH_3_ compared to the Au nanoparticles decorated MoS_2_ sensor.^[^ ^113^ ^]^ The substantial doping of metals at the vacancy sites leads to lattice strains and promotes the inherently low conductivity of TMDs.^[^ ^113^ ^]^ The poor conductivity and the low electron concentration can be overcome by doping some heteroatoms in SnS_2_ sensors, which promotes the gas sensing reaction.^[^ ^108^, ^115^ ^]^ Moreover, doping is the most influential surface engineering technique in TMD‐based gas sensors, showing excellent sensitivity, selectivity, and the lowest detection limit toward the target gas at room temperature.

#### 4.1.3 Surface Oxidation

TMD materials are more prone to oxidation treatment as the oxidation process forms a dense capping layer on the surface of TMDs and makes them highly stable in harsh atmospheres.^[^ ^116^, ^117^ ^]^ The oxidation process is mainly attributed to forming oxide counterparts on the surface of the respective TMD material.^[^ ^116^, ^117^, ^118^, ^119^, ^120^ ^]^ For instance, the oxidation process of MoS_2_ involves the substitution of sulfur atoms with oxygen, and high oxygen content on the surface leads to the lattice distortion of MoS_2_ and creates a MoO_3_ phase on the surface.^[^ ^116^ ^]^ Different routes like oxygen plasma treatment,^[^ ^116^ ^]^ chemical treatment,^[^ ^121^ ^]^ and thermal oxidation^[^ ^117^, ^120^, ^122^ ^]^ have been employed for the partial oxidation in TMDs. Controlled or partial oxidation in 2D TMDs offers unique properties of their respective oxides, such as better adsorption, catalytic surface, and more device durability in gas sensing applications.^[^ ^117^ ^]^ In a heterojunction, smooth charge transfer between different materials is extremely desirable. Heterostructured nanomaterials synthesized from conventional routes are unstable and affect the gas‐sensing properties.^[^ ^28^, ^97^ ^]^ Contrarily, the in‐situ oxidation method facilitates smooth charge transfer between the participating phases in the heterojunction by developing the second phase from the original semiconducting material.^[^ ^118^, ^123^, ^124^ ^]^ Several novel oxide heterojunctions of TMDs, such as WO_3_/WS_2_,^[^ ^117^, ^121^, ^123^ ^]^ MoO_3_/MoS_2_,^[^ ^116^, ^122^ ^]^ SnO_2_/SnS_2_,^[^ ^124^, ^125^ ^]^ SnO_2_/SnSe_2_,^[^ ^118^, ^126^ ^]^ SnO_x_/SnS,^[^ ^119^ ^]^ SnSe_2_/SnO/SnSe^[^ ^120^ ^]^ and WS_2_–WO_3_/CuO^[^ ^127^ ^]^ have been developed from in situ oxidation to promote the gas sensing properties. Oxidation treatment of TMDs changes the structural properties, tunes the morphology, and effectively prevents the aggregation of two‐dimensional materials, thus enhancing the specific surface area and transfer of free carriers.^[^ ^117^, ^118^, ^125^ ^]^ Additionally, the gas sensing characteristics of materials also improved due to the formation of discrete Schottky junctions like n–n,^[^ ^124^ ^]^ and p–n^[^ ^119^, ^121^, ^123^ ^]^ between the semiconductor and counter oxides. Briefly, a huge, uninterrupted electron transfer occurs at the interface of the two‐phase contact that substantially enhances the gas sensitivity.^[^ ^118^, ^124^ ^]^ Till now, precise oxidation‐controlled TMD materials have been employed for the detection of humidity,^[^ ^116^ ^]^ and noxious gases, such as H_2_,^[^ ^117^, ^122^ ^]^ NO_2_,^[^ ^16^, ^118^, ^119^, ^120^, ^121^, ^124^ ^]^ NH_3_,^[^ ^125^ ^]^ and acetone.^[^ ^123^ ^]^ Unfortunately, the oxidation process leads to oxide formation in TMDs that sometimes hampers their low‐ or room‐temperature gas‐sensing properties.^[^ ^117^, ^123^ ^]^ Rani et. al. designed a ternary heterostructure of SnSe_2_/SnO/SnSe by thermal evaporation and observed the highly selective NO_2_ sensing properties at room temperature (Figure 4e,f).^[^ ^120^ ^]^ The significant response of 256% for 5 ppm of NO_2_ was attributed to temperature‐dependent n–p–n switching between the heterointerface, fast physisorption, and charge transfer (Figure 4e). Theoretical studies found that oxidation engineering in TMDs is well suited for NO_2_ detection due to higher S and Se vacancies after the oxide formation on the surface, which is well proven from the experimental results of the different research groups worldwide.^[^ ^118^, ^119^, ^120^, ^121^, ^124^ ^]^

#### 4.1.4 Surface Functionalization

**Figure 5:**
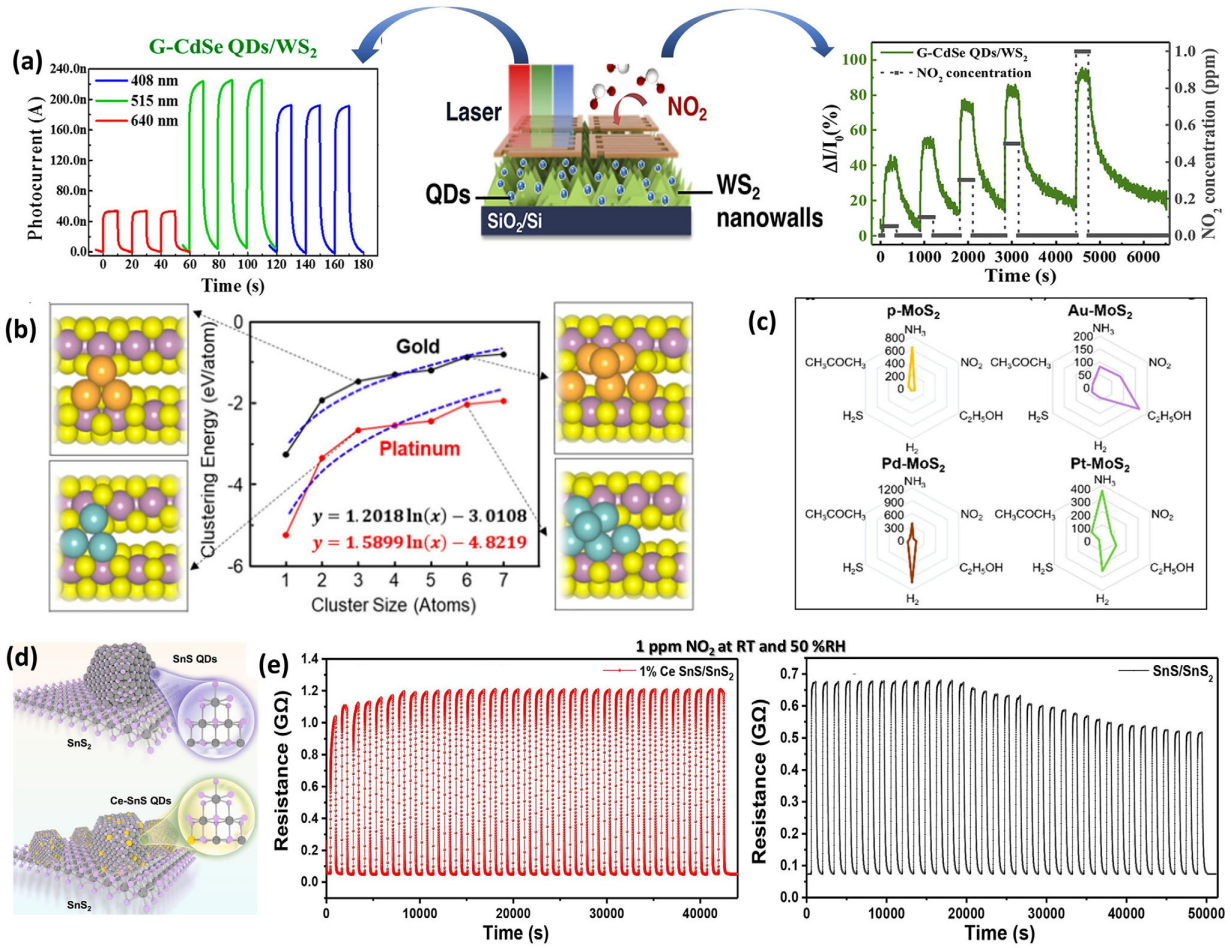
Surface functionalization and single‐atom engineering in TMDs‐based gas sensors. a) core shell CdSe‐ZnS quantum dots−3D WS_2_ nanowall hybrid nanostructures for bifunctional NO_2_ and photo sensing applications. Reproduced with permission.^[^ ^16^ ^]^ Copyright 2020, American Chemical Society. b) Different metal atom clustering energies at the MoS_2_ edge region. c) Radar plot of the response of p‐MoS_2_, Pd‐MoS_2_, Au‐MoS_2_, and Pt‐MoS_2_ for different types of gases. Reproduced with permission.^[^ ^65^ ^]^ Copyright 2023, American Chemical Society. d) Schematic illustration of before and after the addition of Ce SAC in SnS/SnS_2_ nanostructure. e) Stability comparison of 1% Ce‐SnS/SnS_2_ and SnS/SnS_2_ sensor to 1 ppm NO_2_ at RT and 50% RH. Reproduced with permission.^[^ ^146^ ^]^ Copyright 2023, Elsevier.

The surface functionalization route is probed in detail due to its catalytic effect on the pristine sensing layer and significantly enhanced gas sensing performance. Surface functionalization by nanoparticles^[^ ^128^, ^129^, ^130^, ^131^, ^132^, ^133^, ^134^, ^135^, ^136^, ^137^ ^]^ and quantum dots^[^ ^16^, ^138^ ^]^ is more favorable due to their smaller size, fast electron transfer kinetics, and high surface reaction kinetics compared to other proposed morphologies in surface functionalization. Incorporation of nanoparticles tends to form clusters, and the uncontrolled addition can hinder the gaseous interaction on the sensing layer.^[^ ^128^, ^129^, ^139^ ^]^ Generally, the chemical route is widely explored for nanoparticle functionalization, but is limited due to cluster formation or aggregation and nonuniformity of the particles on the sensing channel.^[^ ^139^ ^]^ However, sophisticated techniques like atomic layer deposition (ALD) assisted in the defect‐selective association of metal nanoparticles and yielded highly effective gas sensing capabilities even at room temperature.^[^ ^128^, ^140^ ^]^ Quantum dot sensitization involves the addition of QD receptors to the high‐energy binding sites.^[^ ^16^ ^]^ These quantum dots have abundant surface defects, and the quantum effect is capable of the most active interaction with the target gas analytes compared to the pure semiconducting TMD layer.^[^ ^16^, ^138^ ^]^ Tang et. al. fabricated a colloidal CdS/ZnS or CdSe/ZnS core−shell QD−3D WS_2_ nanowalls hybrid device for bifunctional sensing performance.^[^ ^16^ ^]^ The device efficiently worked as a photodetector, and CdSe QDs/WS_2_ under 515 nm showed the highest photocurrents, approximately ∼4.7 times higher than the pristine material (**Figure** **5**a). Additionally, the CdSe QDs/ WS_2_ hybrid device showed an excellent response of 95.7% in a response time of 26.8 s for 1 ppm of NO_2_ gas with the lowest detection limit of 50 part per billion (ppb) at RT. The sensor shared the advantages of a high aspect ratio of nanowalls, nanosized QDs, and plenty of p–n Schottky junctions between participating materials, promoting superior gas sensing.^[^ ^16^ ^]^ Notably, noble metals (Au,^[^ ^131^, ^139^, ^141^ ^]^ Pt,^[^ ^128^, ^131^, ^132^, ^140^ ^]^ and Pd,^[^ ^129^, ^132^ ^]^ and Rh,^[^ ^136^ ^]^ etc.) addition on the surface of TMD materials is the most viable way to achieve the best gas sensing efficiency. Apart from noble metals, other metals such as Co,^[^ ^130^ ^]^ PtS,^[^ ^141^ ^]^ and PbS^[^ ^138^ ^]^ were also incorporated in pure TMDs for better sensing properties. Most metal nanoparticles participate in sensing in three possible ways: electronic sensitization, spillover effect, and catalytic effect.^[^ ^139^ ^]^ For instance, Au nanoparticle electronic sensitization on the WS_2_ channel layer, where the work function difference of materials leads to the formation of discrete metal‐semiconductor Schottky junctions.^[^ ^139^ ^]^ This junction creates a depletion layer on the unaltered WS_2_, enhancing the sensing response.^[^ ^131^, ^139^ ^]^ Second, the spillover effect strongly participates in the noble metal catalytic activity on the sensing surface, where nanoparticles help in the dissociation and interaction of gases with the sensing channel.^[^ ^140^ ^]^ The catalytic effect of nanoparticles benefits in achieving the selectivity for a particular gas.^[^ ^65^ ^]^ Extensive investigation on the surface functionalization of TMDs material predicted that desirable selectivity, sensitivity, and room temperature sensing of NH_3_,^[^ ^65^ ^]^ CO,^[^ ^139^ ^]^ and NO_2_,^[^ ^142^ ^]^ gases could be achieved by tuning the concentration of noble metals on the sensing channel. Kim et al.^[^ ^65^ ^]^ The detailed study was focused on Au, Pd, and Pt noble metals NP decoration on the 2D MoS_2_ to showcase the selectivity in chemical sensor arrays. They elaborately control the selectivity of MoS_2_ toward NH_3_, H_2,_ and C_2_H_5_OH gases by manipulating the noble metals Au, Pd, and Pt. Understanding H_2_ sensing is quite easy, where the highly oxidized surface of noble metals is extremely favorable for the adsorption of electron‐donating H_2_ analytes. However, the explanation of NH_3_ selective sensing is quite complicated because noble metal particles block the binding site of NH_3_ on MoS_2_, consequently decreasing sensitivity (Figure 5b).^[^ ^65^ ^]^ Here, the coverage of nanoparticles on MoS_2_ will play a pivotal role, and Au nanoparticles have the highest coverage on the MoS_2_ edge, resulting in the lowest NH_3_ sensing. However, the pure MoS_2_ sensor was found highly selective toward NH_3_ gas compared to other metal nanoparticles functionalized MoS_2_ sensors, as shown in Figure 5c. Interestingly, metal nanoparticles efficiently act as an active center for visible light and boost the visible light adsorption in the pristine material.^[^ ^64^ ^]^ Duan et. al. reported a Rh metal loaded SnS/WS_2_ ternary heterostructure for the detection of NO_2_ gas with improved baseline drift and sensing characteristics.^[^ ^136^ ^]^ The density functional theory also proved that Rh nanoparticles are better synergistic catalysts for NO_2_ detection, and a highest sensitivity of 2.83 for 64 ppm NO_2_ with a fast response/recovery time of 6/33 s at 157 °C was observed. Light‐activated gas‐sensing can be successfully assisted by the localized surface plasmon resonance of Au nanoparticles, which encourages electron rearrangement, facilitates charge transfer, and enhances gas molecule adsorption.^[^ ^64^, ^143^ ^]^ Apart from mono metals, bimetallic nanoparticles such as Pd/Pt,^[^ ^144^ ^]^ and Au/Pt,^[^ ^145^ ^]^ sensitize the TMDs effectively for gas sensing. Researchers claimed that bimetals can maximize the catalytic performance on the sensing surface by forming new energy levels and leading to more gaseous interaction.^[^ ^145^ ^]^

#### 4.1.5 Single Atom Catalyst

Recently, single‐atom catalysts (SACs) have drawn huge recognition due to their distinctive activities of atomically distributed metal sites, almost similar chemical and electrical structures, customizable coordination environments, and maximal atom utilization.^[^ ^146^, ^147^, ^148^ ^]^ Single‐atom entities have demonstrated success in catalytic activities and have special promise in chemical identification; yet, their use in chemical sensors and electronic devices is still in its infancy stage.^[^ ^146^, ^147^ ^]^ Surface catalytic enhancement by SAC in TMDs is not widespread, and few research groups are working on the SAC surface engineering route for their sensors.^[^ ^146^, ^147^ ^]^ Single metal atom functionalization also exhibits greater potential and shows enhanced catalytic efficiency. Not only catalysis, but SACs on the TMD channel layer can also play a vital role in the passivation of materials from harsh environments.^[^ ^146^ ^]^ Few metal SACs like Pt, Co, Ru, and Ce have been added to MoS_2_ nanosheets for the efficient detection of VOCs at room temperature.^[^ ^147^ ^]^ Li et. al. provided a new insight by fabricating a Ce single atom targeted SnS/SnS_2_ heterostructure to achieve better sensing characteristics as shown in Figure 5d.^[^ ^146^ ^]^ The sensor displayed high sensitivity (22.1–1 ppm) and low power consumption for NO_2_ sensing at room temperature. The Ce SA creates a passivation layer on the SnS/SnS_2_ heterostructure, effectively minimizing oxidation of SnS quantum dots and resulting in excellent repeatability and long‐term stability (Figure 5e). The research aimed to control the size of SnS quantum dots by the incorporation of single Ce metal atoms. They found that by varying the amount of Ce atoms, the size of SnS quantum dots can be tuned on the surface of the SnS_2_ channel layer. Additionally, Ce SA combines with SnS and forms a Sn‐S‐Ce bond, which effectively diminishes the oxidation of SnS quantum dots and preserves the catalytic activities.^[^ ^146^ ^]^

### 4.2 MXenes

**Figure 6:**
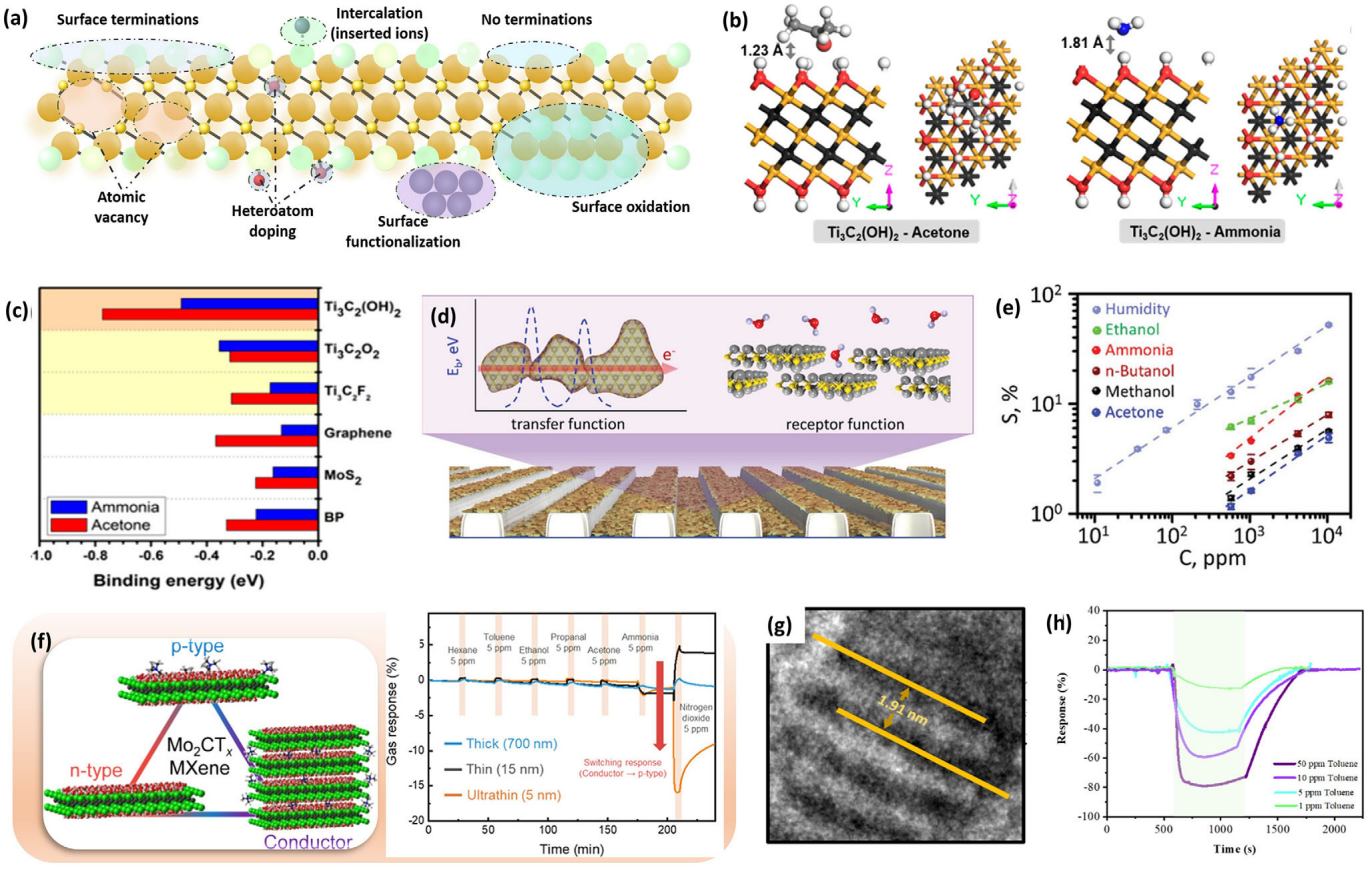
Examples of surface engineering strategies in MXene‐based gas sensors. a) Schematic representation of surface engineering strategies in MXene. Reproduced with permission.^[^ ^66^ ^]^ Copyright 2024, Elsevier. b) Minimum energy representation of acetone and ammonia gas (Lateral and top views). c) Calculated minimum binding energies of acetone and ammonia for different 2D materials. Reproduced with permission.^[^ ^155^ ^]^ Copyright 2018, American Chemical Society. d) The concept of the effect measured through multiple chip electrode configuration: the transfer and the receptor functions are driven by barrier‐controlled electron transport, and the analyte's adsorption over the crystal surface, respectively. e) Concentration versus response curve of H_2_O and other analytes. Reproduced with permission.^[^ ^40^ ^]^ Copyright 2021, Wiley‐VCH. (f) schematic view of the conductivity transition and thickness‐dependent change in gas responses between n‐type, p‐type, and conductor‐type Mo_2_CT_x_ MXene. Reproduced with permission.^[^ ^164^ ^]^ Copyright 2022, American Chemical Society.g) HRTEM showing the interlayer spacing of sulfur‐doped Ti_3_C_2_T_x_ MXene. h) The response curve of sulfur‐doped Ti_3_C_2_T_x_ MXene toward different concentrations of toluene vapor. Reproduced with permission.^[^ ^167^ ^]^ Copyright 2020, American Chemical Society.

MXene is marked as a next‐generation gas sensing material due to its astonishing surface, chemical, physical, and electronic properties.^[^ ^9^, ^40^, ^61^, ^62^ ^]^ It's a non‐deniable fact that MXene will overpower conventional sensing materials like metal oxides and other 2D materials like graphene and its derivatives shortly.^[^ ^35^ ^]^ The advancement of MXene‐based sensors leads to mixing MXene with other foreign sensing materials or modulating the surface properties of MXene by different engineering routes.^[^ ^9^, ^35^, ^61^ ^]^ A substantial amount of research has been done on the surface treatment of pure MXenes to preserve their intrinsic electronic properties and make them more appropriate for various gas‐sensing environments.^[^ ^35^, ^36^, ^66^ ^]^ In the case of 2D MXenes, a variety of surface properties modulation routes include active site modulation, intercalation and delamination of ions, heteroatom doping, surface partial oxidation/reduction, single atom catalyst modulation, and surface functionalization by various nanoparticles. Surface engineering enables the tuning of surface functionalities of MXene, offering opportunities to develop more accurate gas sensing solutions.^[^ ^9^, ^35^ ^]^ This part of the review article will discuss various surface engineering routes of MXene for advancements in gas sensors, as shown in **Figure** **6**a and Table 1.

#### 4.2.1 Active Site Engineering

Active site engineering involves the modulation of vacancies and reactive sites on the surface of MXene sheets. Defect modulation is coined to be strongly recommended due to a drastic change in metallic and surface properties in the MXene material.^[^ ^9^, ^61^, ^62^ ^]^ The metallic conducting of the MAX phase can be tuned by using suitable chemical etchants.^[^ ^9^, ^149^ ^]^ The etching results in the addition of different structural defects and functional groups on the surface of MXene and shows optimum semiconducting characteristics.^[^ ^9^, ^61^, ^149^, ^150^ ^]^ The abundance of these structural defects can cause uncontrolled oxidation in MXene, resulting in poor reliability in performance.^[^ ^149^ ^]^ The treatment with suitable etchants somehow alters the atomic defect density in MXene sheets and changes their properties significantly.^[^ ^9^, ^151^ ^]^ To make MXene more activated for sensing etching is not limited to the chemical etchant (HF,^[^ ^9^, ^40^, ^62^ ^]^ LiF‐HCl,^[^ ^151^, ^152^ ^]^ NaF‐HCl,^[^ ^152^ ^]^) but also a few different techniques like ultrasonic waves,^[^ ^152^ ^]^ UV assisted,^[^ ^153^ ^]^ and surfactants,^[^ ^154^ ^]^ have been explored. In the pristine state, most of the MXenes are metallic conductive, and their properties can be tuned by changing the proportion of surface terminations.^[^ ^9^, ^41^ ^]^ Metallic MXenes result in low noise signals but are compromised in sensitivity toward the gas. A strong metallic conductivity but still abundant functional groups for detecting the signal was the key point in the newly introduced Nb_2_CT_x_ MXene etched with cationic surfactant like cetyltrimethylammonium bromide (CTAB).^[^ ^41^ ^]^ For instance, the Ti_3_AlC_2_ MAX phase after etching forms hydrophilic Ti_3_C_2_T_x_ nanosheets, where T_x_ stands for the surface terminations such as –OH, –O, or –F groups, which significantly affect surface electrochemical properties.^[^ ^61^, ^149^ ^]^ The edge enrichment with these groups modifies the gas adsorption or desorption capacity, electronic work function, and charge transfer tendency, offering its wide employment in gas sensors.^[^ ^9^, ^61^, ^66^ ^]^ Zhao et al. endowed their work by proposing rich terminal groups edge enrichment in high‐active double transition‐metal titanium molybdenum carbide (Mo_2_TiC_2_T_x_) based portable, wireless NO_2_ sensor for gas leakage searching at room temperature.^[^ ^9^ ^]^ Etched MXenes are highly reactive and show strong gas adsorption strength but poor selectivity toward a specific gas, making them less reliable for target‐selective gas sensing.^[^ ^9^, ^61^ ^]^ DFT simulations predicted that oxygen‐modulated active sites of MXenes are more compatible with the selective detection of a particular gas and narrow down the choice of MXene for selective gas sensors.^[^ ^150^ ^]^ Different functional groups on MXene behave differently in gas sensing, such as hydroxyl (–OH) terminated MXene has stronger adsorption compared to oxygen (–O) terminated MXene.^[^ ^62^, ^155^ ^]^ The type of etchant highly contributing factor, and the surface chemistry of MXene can be easily tuned with the same chemical composition.^[^ ^151^, ^152^ ^]^ The small adsorption distance between Ti_3_C_2_(OH)_2_ and gas analytes supports the higher interaction and more gaseous response during the sensing study (Figure 6b).^[^ ^155^ ^]^ Ti_3_C_2_(OH)_2_ showed the highest binding energy and short equilibrium distance for acetone and ammonia compared to other Ti_3_C_2_O_2_, Ti_3_C_2_F_2_, and 2D materials (Figure 6c).^[^ ^155^ ^]^ The high aspect ratio of MXene helps in achieving the low‐noise resistance signal and outcome in target analyte detection at the lowest concentration possible. Pazniak et al. proved this by detecting the lowest value of H_2_O reported until now.^[^ ^40^ ^]^ They implemented a reversible detection technique and tested down to 10 ppm of humidity with the lowest signal‐to‐noise ratio compared to humid vapor (Figure 6d,e). The sensor showed the highest response for the water vapor compared to the VOCs in different concentration ranges, proving the higher affinity toward the H_2_O analytes (Figure 6e). The variation in substrate (glass, crystalline, or porous silicon) can also change the defect distribution in MXene and influence its sensing characteristics.^[^ ^153^ ^]^

#### 4.2.2 Intercalation/Delamination

Surface intercalation or delamination is a rapid attempt to enhance surface functionality and gaseous reactivity in pristine MXenes.^[^ ^24^, ^35^ ^]^ Intercalation is explained by the exfoliation of 2D MXene layers and the addition of intercalants.^[^ ^24^, ^156^ ^]^ Research groups are engrossed in the design of extremely active MXene‐based sensors using various ion intercalation concepts. Intercalation of ions like K, Mg, Li, Ca, Cl, and Na was done using different salts KCl, NaOH, NaCl, CaCl_2_, and MgCl_2_, after the etching process.^[^ ^157^, ^158^, ^159^, ^160^, ^161^ ^]^ Ionic intercalation offers a workaround for modulating the conductivity by augmenting the MXene carrier density.^[^ ^25^, ^159^, ^160^ ^]^ The intercalation mechanism for MXene is quite different from other layered 2D materials like graphene, TMDs, and so on.^[^ ^24^, ^156^ ^]^ Multiple planes of MXene with separable interlayers and the rigidity of these layers result in the random placement of guest molecules in the interlayer spacing.^[^ ^159^, ^161^, ^162^ ^]^ The penetration of intercalants in the MXene layer changes its bonding, and hybridization of orbitals and energy levels consequently modulates the electrical, thermal, and optical properties.^[^ ^163^, ^164^ ^]^ Theoretical and experimental studies proved that intercalation can induce the transition between metallic and semiconducting behavior by modulating the interlayer spacing of MXenes.^[^ ^163^, ^164^ ^]^ Choi et. al. showed an n−p‐conductor three‐phase transition by modulating the organic intercalants and film thickness in the Mo_2_CT_x_ MXene gas sensor as shown in Figure 6f.^[^ ^164^ ^]^ The researchers found that 5‐nm‐thick Mo_2_CT_x_ MXene intercalated with tetramethylammonium hydroxide (TMAOH) showed a p‐type response, while without intercalation, MXene showed a clear n‐type response.

Additionally, MXene with a thickness of 700 nm exhibited a conductor‐type response toward the NO_2_ gas analytes (Figure 6f). The response value was also thickness‐dependent and varied in Mo_2_CT_x_ MXene sensors (Figure 6f). Ion‐intercalated MXenes were effectively utilized to detect gases like ammonia, trimethylamine, ethanol, CO_2_, formaldehyde, methane, and so on.^[^ ^25^, ^157^, ^158^, ^159^ ^]^ Not only gases and vapor, Muckely et al. performed a detailed structural, electrical, and gravimetric study on water vapor interaction with ion‐intercalated MXenes for multimodal humidity sensors.^[^ ^25^ ^]^ K and Mg intercalants between MXene layers significantly increase the spacing between MXene layers with a tiny quantity of hydration. Nonetheless, K^+^ and Mg^2+^ ions directed to the insertion of 2 and 5 H_2_O molecules per ion, respectively, lead to an increase in c‐lattice parameters and show thresholds of ∼0.8% RH for relative humidity (RH) detection.^[^ ^25^ ^]^

#### 4.2.3 Doping Engineering

Doping in 2D nanomaterials is an intriguing area of research, and doped MXene emerged as a promising candidate for gas sensing applications.^[^ ^165^, ^166^, ^167^ ^]^ However, doped MXene‐based sensors are still in their infancy and need more attention from researchers. Doping in MXene can be done in three possible ways where substitution of an atom from the parent MXene, dopant atoms at the interstitial sites of the MXene lattice, and lastly, covering of the active sites of the MXene layer with the dopants (surface adsorption) covers all doping possibilities in MXene.^[^ ^66^ ^]^ All three routes significantly modulate the bandgap, lattice structure, and interlayer spacing of MXene. Heteroatoms, mainly nitrogen, sulfur‐doped MXene sensors, showed stronger molecular adsorption than undoped MXene.^[^ ^165^, ^166^, ^167^ ^]^ For instance, sulfur has high electronegativity, which can cause a decrease in Ti electron density and form strong bonds (S─Ti─C) on the interface in pure Ti_3_C_2_T_x_ MXene.^[^ ^167^ ^]^ This process promotes the creation of a wider electron‐depleted space‐charge layer, as shown in the HRTEM image in Figure 6g. The sensor also showed a significant change in device current after exposure to different concentrations of toluene gas compared to undoped Ti_3_C_2_T_x_ at room temperature (Figure 6h). N as dopant is more popular than other dopants in MXene‐based gas sensors because it provides more conductivity, carrier scattering, and charge doping after gas adsorption.^[^ ^165^, ^166^, ^167^ ^]^ Metallic MXene with no signals showed an inspiring response to a low concentration of NH_3_ after the N‐doping, confirming the positive influence of doping on the MXene.^[^ ^165^ ^]^

#### 4.2.4 Surface Oxidation

**Figure 7:**
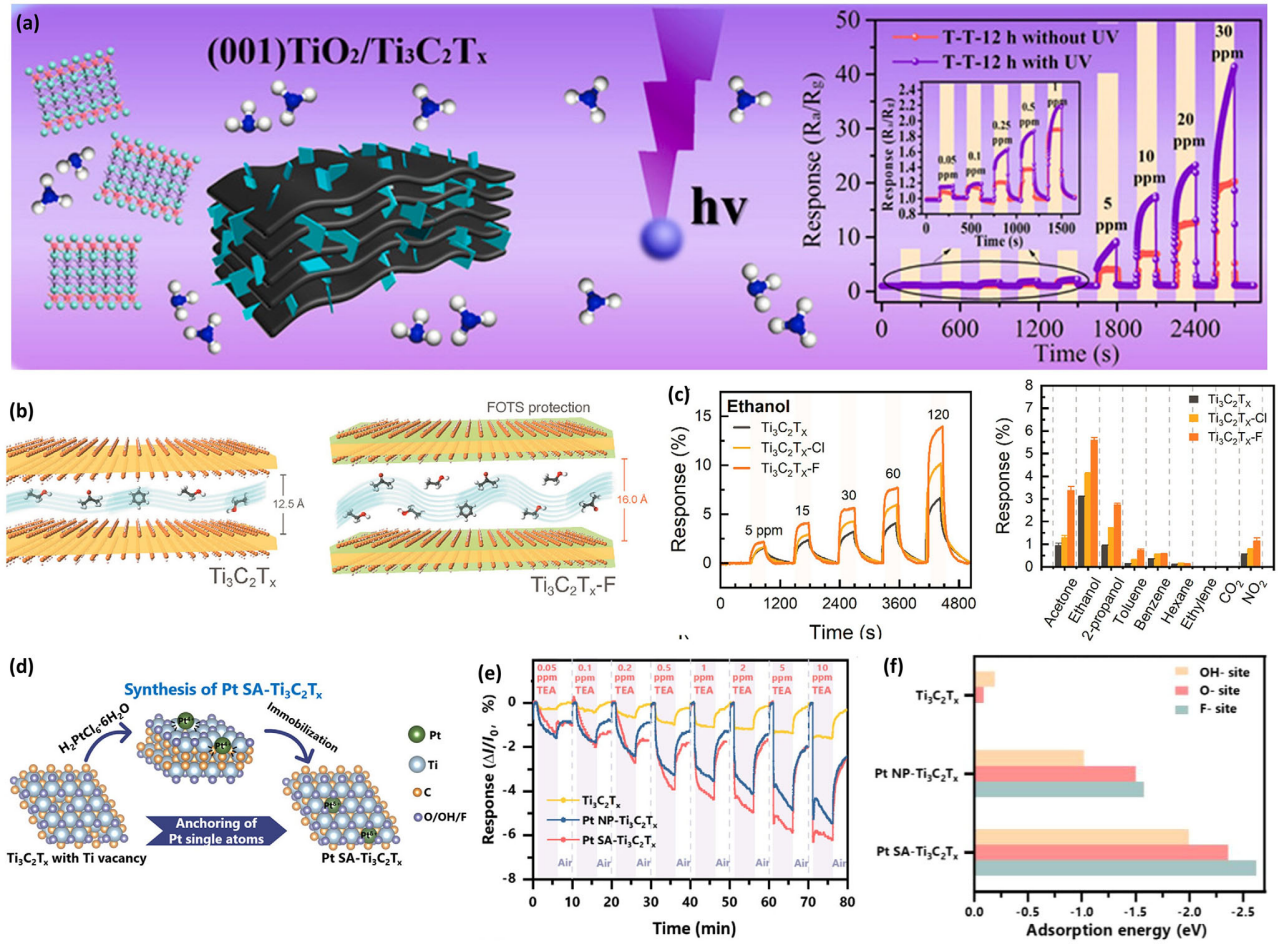
Surface engineering in MXene‐based gas sensors. a) Schematic of (001) TiO_2_/Ti_3_C_2_T_x_ based sensor and NH_3_ sensing response curve of the composite with and without UV light illumination and employment of the sensor for the food spoilage application visible in the block diagram of the integrated circuit alarm system to monitor the fish current status in different forms like “no decay,” “start to decay,” and “decay.” Reproduced with permission.^[^ ^26^ ^]^ Copyright 2022, Elsevier. b) Schematics of increased interlayer spacing of Ti_3_C_2_T_x_ MXene after functionalization (Ti_3_C_2_T_x_‐F) upon exposure to VOCs. c) Dynamic response curves toward varying ethanol concentration from 5 to 120 ppm and selectivity study of the Ti_3_C_2_T_x_, Ti_3_C_2_T_x_‐Cl, and Ti_3_C_2_T_x_‐F sensors. Reproduced with permission.^[^ ^174^ ^]^ Copyright 2020, American Chemical Society. d) Schematic representation of the synthesis process of Pt SAC‐Ti_3_C_2_T_x_ MXene. e) Response behavior of different MXene functionalized sensors toward a range of TEA concentrations. f) DFT models and calculated adsorption energy of TEA molecules on different edge sites (OH−, O−, and F−) on Pt SAC‐Ti_3_C_2_T_x_, Pt NP‐Ti_3_C_2_T_x_, and pristine Ti_3_C_2_T_x_. Reproduced with permission.^[^ ^188^ ^]^ Copyright 2022, American Chemical Society.

The stability of MXene is always a concern because it gets even degraded in normal ambient conditions. The quick oxidation of MXene at room temperature in an aqueous solution raises a concern about stability in MXene‐based devices for different applications.^[^ ^35^ ^]^ In the case of Ti‐based MXenes like Ti_3_C_2_T_x_, and Ti_3_C_2_ oxidation results in the formation of TiO_2_ and hampers structural, chemical, and electronic properties.^[^ ^168^, ^169^, ^170^ ^]^ MXenes have poor long‐term oxidation stability in liquid compared to the freestanding MXenes. MXene gets oxidized fastest in aqueous solution, even at room temperature.^[^ ^35^ ^]^ Although the unique 2D properties of MXenes withstand after heating at a higher temperature of 600 °C.^[^ ^171^ ^]^ Oxidation degrades the long‐term stability of MXene ‐based devices and to overcome this issue, researchers have proposed many mitigation strategies such as; controlling the defects to alter the degree of oxidation,^[^ ^149^ ^]^ thermal treatment in inert atmosphere to modulate the surface groups^[^ ^171^ ^]^ and thermal stability, storage conditions can inhibit the fast oxidation of MXenes^[^ ^35^ ^]^ and introduction of antioxidant agents or a protective layer^[^ ^93^ ^]^ showed promising results in reported works. These techniques envisage that MXenes with fewer defects and non‐aqueous devices are less prone to self‐oxidation and retain their electronic properties.^[^ ^35^, ^149^, ^171^ ^]^ Unwanted oxidation degrades the performance of MXene devices, but the controlled oxidation is harnessed to create high performance MXene‐based gas sensor.^[^ ^168^, ^169^ ^]^ In MXene, partial and controlled oxidation in titanium carbide^[^ ^170^ ^]^ and vanadium carbide directly form oxide counterparts^[^ ^169^ ^]^ and can enhance the gas sensing performance.^[^ ^171^ ^]^ The controlled thermal oxidation of titanium carbide at a certain temperature improved the gas sensing performance.^[^ ^170^, ^171^, ^172^ ^]^ Authors claimed that titanium carbide MXene easily formed stable titanium oxide after the thermal treatment, and controlled oxide formation actively participates in astonishing gas sensing properties.^[^ ^170^, ^171^, ^172^ ^]^ The synthesis route of oxide in MXene also plays a key role in the morphology tuning. For instance, physically oxidized MXene^[^ ^170^, ^171^ ^]^ does not show any significant change in sheet morphology, whereas the chemical oxidation^[^ ^26^, ^172^, ^173^ ^]^ results in the growth of oxide nanoparticles throughout the MXene sheets and modulates the resulting morphologies. These small oxide nanoparticles also provide a more specific surface area for gaseous interaction and improve the surface properties.^[^ ^26^, ^170^, ^171^, ^172^ ^]^ Zhang et. al. reported significantly visible morphological changes in hydrothermally treated MXene due to the Ti_3_C_2_ transformation in Ti^+3^ ions and later accumulation in TiO_2_ at the defective sites (**Figure** **7**a).^[^ ^26^ ^]^ In this study, TiO_2_ with a highly active (001) crystal plane showed an efficient photogeneration under UV illumination and showed a 34 times higher response for ammonia (30 ppm) than that of Ti_3_C_2_T_x_ MXene (Figure 7a). Lastly, an integrated circuit alarm system including near‐field communication and a microcontroller was designed to detect food spoilage detection where the decay process of fresh pork, fish, and shrimp was optimized in different states (Figure 7a). The partial oxidation contributes to sensing by increasing the number of defects or oxygen functional groups and interfacial properties of discrete metal/semiconductor Ti_3_C_2_T_x_/TiO_2_ junctions.^[^ ^39^, ^170^, ^172^ ^]^ The oxide establishment in MXene facilitates high charge carrier transport at the interface of the heterojunction and results in more gaseous analyte interaction than the pristine MXene.^[^ ^169^, ^172^ ^]^ Remarkably, it was evident that partially oxidized MXene has more capabilities in VOC sensing and showed selective and highly sensitive behavior to different VOCs like: acetone,^[^ ^169^ ^]^ ethanol,^[^ ^170^ ^]^ and hexanal^[^ ^173^ ^]^ as compared to pure MXene. Moreover, the high thermal activation energy of oxides leads to a drawback in MXene‐based gas sensors by working at high operating temperatures.^[^ ^170^, ^171^, ^172^ ^]^

#### 4.2.5 Surface Functionalization

Surface functionalization of materials is always a better approach to achieve enhanced gas sensing performance. In MXene, surface functionalization with different metals or materials is widely explored compared to classical doping engineering.^[^ ^174^, ^175^, ^176^, ^177^, ^178^, ^179^, ^180^, ^181^, ^182^, ^183^, ^184^, ^185^, ^186^, ^187^ ^]^ Abundant surface terminations in MXenes make the process straightforward, and surface functionalization opens an opportunity to introduce the desired surface terminations in the material to control their adsorption affinity.^[^ ^35^ ^]^ MXene functionalization with FOTS,^[^ ^174^ ^]^ ‐NH_2_,^[^ ^175^ ^]^ Ag,^[^ ^176^ ^]^ Au/Pt,^[^ ^177^ ^]^ PbS,^[^ ^178^ ^]^ gelatin,^[^ ^179^ ^]^ Pd,^[^ ^180^ ^]^ (Fe_2_(MoO_4_)_3_),^[^ ^181^ ^]^ titanium oxyfluoride (TiOF_2_),^[^ ^182^ ^]^ Pt@SnS_2_,^[^ ^183^ ^]^ Au,^[^ ^184^ ^]^ V_2_O_5_,^[^ ^185^ ^]^ Pt,^[^ ^186^ ^]^ and CuZnS^[^ ^187^ ^]^ nanoparticles effectively manipulate the gas sensing properties. Not only surface termination but surface functionalization effectively changes the interlayer spacing of 2D sheets, consequently increasing the specific surface area. Chen et. al. gathered great attention by introducing a self‐assembled monolayer (SAM) of fluoroalkylsilane (FOTS) on the MXene sheets to mitigate the inherent oxidative instability of MXene.^[^ ^174^ ^]^ Moreover, FOTS molecules functionalized MXene showed a higher specific surface area (32.9 m^2^ g^−1^) caused by increased interlayer spacing from 12.5 to 16 A° as shown in Figure 7b. Compared to pristine, the SAM‐functionalized Ti_3_C_2_T_x_ MXenes exhibited high adsorption and diffusion of oxygen‐containing volatile organic compounds (ethanol) and showed the highest response for different concentrations at room temperature, as shown in Figure 7c. The FOTS functionalized MXene also showed selectivity toward the oxygen‐containing volatile organic compounds (ethanol, acetone) compared to other interfering gases (Figure 7c). Noble metals such as Ag nanoparticles,^[^ ^176^ ^]^ Pd colloidal nanoclusters,^[^ ^180^ ^]^ and Au, Pt nanoparticles,^[^ ^177^, ^183^, ^186^ ^]^ functionalized MXene possess high conductivity and catalytic properties, which assist in more sensitive gas detection. Noble metals alter the amount of free charge carriers in MXene, which alters the fermi level position, and a significant change in sensor performance can be achieved.^[^ ^177^, ^180^ ^]^ Ti_3_C_2_/Ag nanoparticles with optimum Ag concentration displayed 15 times higher sensitivity toward the humidity than the pure Ti_3_C_2,_ but after a certain Ag concentration, a drop in performance was reported.^[^ ^176^ ^]^ In some studies, researchers tried to enhance sensing performance by the sensitization of PbS quantum dots,^[^ ^178^ ^]^ amine functionalization,^[^ ^175^ ^]^ MXene/gelatin ink,^[^ ^179^ ^]^ iron molybdate (Fe_2_(MoO_4_)_3_),^[^ ^181^ ^]^ and TiOF_2_,^[^ ^182^ ^]^ with MXene for room temperature sensing of different gases. In an interesting study, a Ti_3_C_2_T_x_ MXene/gelatin ink was synthesized for patterning electrodes on a paper substrate.^[^ ^179^ ^]^ Wang et. al. designed a 3D MXene‐origami to successfully recognize the direction and height distribution of NH_3_ which resulted in a good response of 7% to 50 ppm of NH_3_.^[^ ^179^ ^]^ 3D mesostructured MXene origami biodegradable sensor demonstrated the capability to identify the direction and height distribution of hazardous gases. The surface functionalization was performed to achieve a higher bandgap, and affinity for easy gaseous adsorption, stability of the MXene film, and catalytic reactions by stabilizing the surface terminal groups in MXene sensors.

#### 4.2.6 Single‐Atom Catalyst

MXene materials support single‐atom catalyst functionalization due to the presence of huge cationic vacancies.^[^ ^66^, ^188^ ^]^ These vacancies support the anchoring of SAC on the MXene surface. For instance, Pt SACs addition on the ultrathin Ti_3_C_2_T_x_ nanosheets by a novel self‐reduction immobilization process was done to produce Pt SA‐Ti_3_C_2_T_x_ as shown in Figure 7d–f.^[^ ^188^ ^]^ Pt SACs were immobilized on the defective sites, and strong bonds formed between neighboring C atoms (Pt─C bond). The conductive channel was used to fabricate a field effect transistor (FET) sensor, which showed a huge sensitivity for the ppb concentration of TEA (triethylamine) at room temperature (Figure 7e). With the unique and single‐atom structures of Pt metal, strong adsorption of TEA was facilitated in Pt SA‐Ti_3_C_2_T_x_ than in pristine Ti_3_C_2_T_x_ MXene. The DFT analysis predicted the higher adsorption energy of the SAC functionalized sensor than that Pt nanoparticles functionalized sensor, showing a big opportunity for SAC‐engineered gas sensors to achieve desirable sensing outcomes (Figure 7f). The catalytic activity of single atoms provides rich active sites for faster dissociation and reduces the oxidation activation energy for the target gas.^[^ ^188^, ^189^ ^]^ In another report, Chen and his coworkers performed in situ anchoring of Ni single atoms in the lattice of MXene analogue (TiC_0.5_N_0.5_) to achieve larger charge transfer from Ni atoms to adjacent Ti atoms.^[^ ^190^ ^]^ The replacement of Ti sites with Ni single atoms causes a significant increase in electron density for NO_2_ absorption. The sensor with abundant defective sites showed an excellent response and an ultra‐low detection limit of 10 ppb for NO_2_ gas at room temperature. Furthermore, humidity influence on MXene sensors is well known, and researchers have tried to overcome the humidity interference on the sensing performance of MXene by incorporating the Ag atomic clusters on a single layer and multilayered Ti_3_C_2_T_x_ sheets.^[^ ^191^ ^]^ The Ag‐MXene and multilayered MXene sensors were more sensitive to NH_3,_ and the Ag‐MXene sensor performed best at 40% RH and exhibited the best humidity‐resistant nature. DFT study revealed that Ag‐Ti_3_C2T_x_ heterojunction can also increase the adsorption energy without humidity, explaining the improved humidity resistance for AgT. Moreover, the effective loading of SACs on MXene is a big concern and needs attention in the future.

### 4.3 Nitride (hBN, g‐C_3_N_4_)

2D nitride layered materials such as hexagonal boron nitride (hBN) and g‐C_3_N4 have established their firm presence in gas sensing technology.^[^ ^42^, ^43^, ^44^, ^45^, ^46^, ^47^, ^48^, ^49^ ^]^ Their advanced properties make these materials adaptable in various fields to showcase their extraordinary and reliable performance. The poor signal stability and sensing response toward gas analytes restrict their wide employment in real‐time gas sensors.^[^ ^42^, ^43^, ^48^, ^49^ ^]^ Various surface engineering routes have been explored to enhance the applicability of hBN and g‐C_3_N_4_ two‐dimensional materials in gas sensing applications (Table 2).

#### 4.3.1 Active Site Engineering

**Figure 8:**
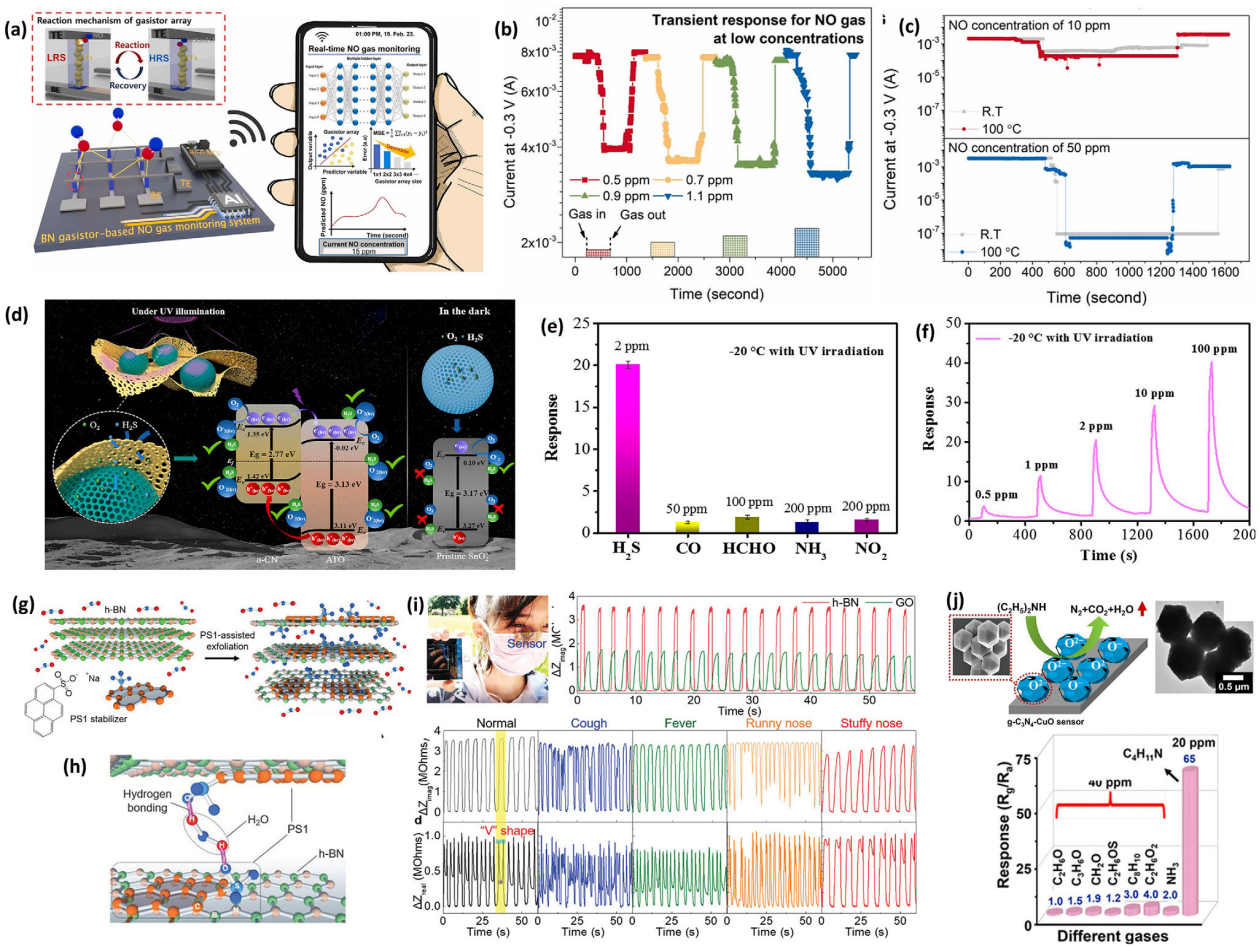
The examples of surface engineering in 2D nitride gas sensors. a) Schematic view of an artificial BN‐based gasistor array for a real‐time NO gas monitoring system. b) Transient response characteristics of NO gas sensor at a range of low concentrations. c) Transient response behavior depending on temperature. Reproduced with permission.^[^ ^44^ ^]^ Copyright 2023, Elsevier. d) Illustration of the gas sensing process of pristine SnO_2_ under dark and ATO@n‐CN sensors under UV illumination at subzero temperature. e) The H_2_S gas selective behavior. f) Transient curve of ATO@4n‐CN gas sensor at −20 °C with UV irradiation. Reproduced with permission.^[^ ^210^ ^]^ Copyright 2023, American Chemical Society. g) Outline of pyrene (PS1) assisted liquid phase exfoliation of h‐BN. h) Sensing mechanism of the h‐BN‐based humidity sensor, which is showing an interaction between PS1 functionalized hBN sheets and H_2_O molecules. i) Real‐time monitoring of human breath by wireless humidity sensor integrated onto a mask, a comparison between hBN and graphene sensor and changes in the Imaginary and real parts of the impedance from a volunteer having common symptoms of flu, such as coughing, fever, and runny and stuffy nose. Reproduced with permission.^[^ ^46^ ^]^ Copyright 2024, Wiley‐VCH. j) Schematic view of the construction of g‐C_3_N_4_‐CuO heterostructure and the selective sensing of diethylamine (DEA) gas at room temperature (RT). Reproduced with permission.^[^ ^217^ ^]^ Copyright 2024, American Chemical Society.

Pristine hBN is not so well known as an effective gas sensing material, but the last decade portrayed hBN as an excellent substrate material for supporting several 2D materials like graphene.^[^ ^192^ ^]^ Boron nitride as a substrate improves the stability, roughness, and intrinsic local electronic properties of 2D material compared to other substrates like SiO_2_.^[^ ^192^ ^]^ The excellent adhesion properties of the material could be due to the low surface roughness of the hBN substrate. High‐performance and crack‐free materials can be synthesized on the hBN as a sacrificial layer and provide a pathway to enhance sensor performance. hBN offers the synthesis and smooth transfer of materials on the foreign substrate without degrading the sensing performance.^[^ ^193^ ^]^ This hBN transfer technique showed a doubling of the sensitivity to NO_2_ gas and a response time that is six times higher than the transfer from other substrates. hBN is also an excellent substrate for controlling the electronic properties of graphene in molecular self‐assembly by engineered potential landscapes.^[^ ^194^ ^]^ In a recent study, Cadore et al. showcased the extraordinary NO_X_ sensing properties of MoS_2_ on an hBN substrate.^[^ ^195^ ^]^ The transistor device displayed the lowest detection limit of 6 ppb at room temperature, which could be attributed to the lesser number of interfacial traps and disorders in the MoS_2_ on the hBN substrate sensor.^[^ ^195^ ^]^ Many researchers have also marked an impact in hBN‐engineered sensors, where they fabricated metal‐free cataluminescence (CTL) based hBN gas sensors.^[^ ^42^, ^45^ ^]^ Metal‐free materials offer low cost, environmental friendliness, and stability, which have attracted huge attention recently. Chae et. al. designed a high‐accuracy hBN gasistor sensor array for real‐time monitoring of NO gas at a low power of 3.7 mW with a gas analysis using a deep‐learning function (**Figure** **8**a).^[^ ^44^ ^]^ The proposed sensor array showed excellent NO sensing behavior with a high response and a lower detection limit of 0.5 ppm at RT (Figure 8b). The sensor showed a faster response at 100 °C compared to the room temperature because reactions at 100 °C are faster than at RT (Figure 8c).

On the other hand, bulk g‐C_3_N_4_ was converted into two‐dimensional structures to achieve better gas‐sensing activity, but the material still lacks sensing performance.^[^ ^67^ ^]^ The researchers have made a lot of efforts to tune the gas‐sensing properties of 2D g‐C_3_N_4_ by modulating the defect density in the material.^[^ ^47^, ^196^, ^197^, ^198^ ^]^ In general, plenty of N atoms substitute the C atoms of the g‐C_3_N_4_ framework, which permits the material to construct numerous defective sites. During defect generation, N coordination sites participate with the foreign element, resulting in modulated defect density in the material.^[^ ^67^, ^196^ ^]^ Additionally, an increase in interlayer spacing can be observed between the layers of g‐C_3_N_4_ due to the weak van der Waals forces in the graphitic structure of the material, leading to more free‐charge carrier transfer into the material.^[^ ^196^ ^]^ It is proven that highly exfoliated (monolayer) gCN nanosheets have high surface reactivity and huge affinity toward gases with low adsorption energy compared to bulk g‐C_3_N_4_.^[^ ^47^, ^197^ ^]^ Thermal exfoliation of g‐C_3_N_4_ in a specific atmosphere (e.g., O_2_) can create vacancies and result in a high surface area, amino functional group, and inherent porous structure for excess gaseous analyte interaction.^[^ ^47^, ^48^, ^49^ ^]^ g‐C_3_N_4_ offers the possibility to tune the electrical, chemical, and structural (nanosheets,^[^ ^47^, ^48^, ^49^ ^]^ nano‐ribbons^[^ ^198^ ^]^) properties by ultrasonic dispersion. Few of the research groups pay much attention to developing g‐C_3_N_4_ nanostructures‐based surface acoustic wave (SAW) sensors for the sub‐ppb level NO_2_ detection at room temperature.^[^ ^48^, ^198^ ^]^ Pasupuleti et. al. concluded that the high negative frequency shift value of the g‐C_3_N_4_ SAW sensor toward the NO_2_ gas was mainly ascribed to the high mass loading effect induced by the numerous surface defects, and enhanced charge transfer between the sensing surface and NO_2_ molecules.^[^ ^48^ ^]^ Besides, the g‐C_3_N_4_/LGS SAW sensor showed a low detection limit (158 ppb), and selectivity to NO_2_ gas under various relative humidity (RH) conditions (20%–80%) at RT. Design of a 3D hydrogel from 2D g‐C_3_N_4_ sheets using a suitable ionic agent can further enhance the reactive sites of the sensing material.^[^ ^199^ ^]^ Yan et. al. reported a 3D stacking of g‐C_3_N_4_ layered material from bulk 2D material using a simple hydrothermal process, assisted by ionic liquids (ILs).^[^ ^199^ ^]^ The resulting 3D g‐C_3_N_4_ hydrogel gas sensor reported excellent sensitivity (R_a_/R_g_ = 9.89 for 50 ppm H_2_S) and fastest response (less than 10 s) demonstrating an alternative option for enabling gas sensing at low power consumption.

#### 4.3.2 Doping Engineering

Pristine hBN, as a wider bandgap material, shows no significant change in the bias voltage due to its dielectric property and poor surface reactivity, but doping in the material can change the electronic behavior.^[^ ^82^ ^]^ Reports suggested that Mg‐doped hBN layers, where Mg atoms replace the B atom, illustrate better current conduction and modulated electrical conductivity.^[^ ^200^ ^]^ Sajjad et. al. experimentally reported a high‐performance doped Wafer‐scale BN nanosheet gas sensor.^[^ ^22^ ^]^ The nanosheets were doped with carbon elements for the highly selective methane gas sensor. The carbon (C) addition with B and N atoms could lead to the formation of different interlayered crystalline structures in hBN and improve the electronic and semiconducting properties. Relatively, the sensor was poorly sensitive to O_2_ compared to the methane. However, doped hBN has not been employed substantially for gas sensors, and theoretical studies have been carried out to check the applicability of doped nanomaterials in gas sensors.^[^ ^201^, ^202^, ^203^ ^]^ Researchers found that doping causes a structural deformation in the boron nitride.^[^ ^202^, ^203^ ^]^ Different dopants such as Co, Mn, O, and Ti have been incorporated in boron nitride to estimate the sensing characteristics. Further research is needed to determine the ideal doping concentrations for BN‐based gas sensor devices.^[^ ^201^, ^202^, ^203^ ^]^

Doping of foreign elements with a suitable ion radius in g‐C_3_N_4_ is a prevalent strategy to improve the gas sensing performance by modulating the carrier density and band structure.^[^ ^204^, ^205^, ^206^, ^207^, ^208^, ^209^, ^210^, ^211^ ^]^ Some metal or nonmetal doping has been introduced in graphitic carbon nitride to enhance the sensing performance. Interestingly, g‐C_3_N_4_ is a photoactive material, and doping of this material with Fe/Mn,^[^ ^204^ ^]^ carbon,^[^ ^205^ ^]^ sulfur,^[^ ^206^, ^207^ ^]^ and Sb^[^ ^210^ ^]^ can enhance visible light absorption and boost the capability of charge separation. Researchers have proven that g‐C_3_N_4_ shows enhanced gas‐sensing characteristics under visible‐light radiation, and the performance can be further improved by doping with suitable atoms.^[^ ^206^, ^207^, ^210^ ^]^ Pi et. al. reported a UV‐driven in situ H_2_S sensing technique at −20 °C using Sb doped SnO_2_/g‐C_3_N_4_ sensing channel (Figure 8d–f).^[^ ^210^ ^]^ The sensor showed a fast response in 14 s with a response value of 20.1 toward 2 ppm H_2_S at −20 °C, realizing a highly selective H_2_S gas sensor at subzero temperature for the first time (Figure 8e,f). A possible mechanism predicted that Sb doping and UV light combination significantly raise the free carrier concentration, mobility, and interaction between the gas and sensing channel. g‐C_3_N_4_ as an excellent photosensitizer effectively facilitates the separation and transport of charge carriers under light illumination, and SnO_2_ is proven to be the best host material to incorporate Sb dopants to create an effective donor level (Figure 8d). Sulfur dopants destroyed the interlayer structure of g‐C_3_N_4_ and caused lattice distortion, which results in the enhancement of defect states and active sites for the physicochemical reaction.^[^ ^206^, ^207^ ^]^ Gao et. al. introduced polyoxometalates (POMs) kind of inorganic anion metal‐oxide heterogenous dopant, to overcome the slow response recovery times and low response values drawbacks of g‐C_3_N_4_ nanostructure.^[^ ^211^ ^]^ These POMs worked as electron acceptors and separated them from holes, leading to a decrease in electron‐hole recombination rate and a boost in acetone sensing performance. Nb,^[^ ^208^ ^]^ B,^[^ ^209^ ^]^ and other different dopants are also incorporated with the g‐C_3_N_4_ and showed a worthwhile influence on the sensing characteristics of NH_3_ and NO_2_ gases, respectively. The addition of dopants not only shifts the band gap but also tunes the conductivity (n‐type to p‐type) of the g‐C_3_N_4_ material.^[^ ^209^ ^]^ Incorporation of atomically similar dopants like B in g‐C_3_N_4_ can easily replace the C and substantially change the surface chemical properties. Pasupuleti et. al. proved from their experimental studies that substitutional doping of electron‐deficient B atoms with C atoms induces the generation of impurity states, which results in the reduction in the bandgap, enrichment in carrier mobility, and high chemical reactivity.^[^ ^209^ ^]^ Fascinatingly, B‐doped g‐C_3_N_4_ SAW sensor exhibited an excellent sensitivity (Δ*f* = 35.97 kHz/50 ppm), ultra‐low detection limit (DL∼7.9 ppb), and fast response/recovery times (35 s/43 s) under UV light illumination.

#### 4.3.3 Surface Functionalization

The functionalization of hBN involves several physical and chemical processes for gas‐sensing applications.^[^ ^46^, ^212^ ^]^ The limited exploration of hBN in gas sensing is due to its high resistance, poor selectivity, and inert nature.^[^ ^82^ ^]^ Metal nanoparticle functionalization on hBN resulted in high thermal conductivity and defect‐induced surface reactivity, which helps in low‐temperature gaseous diffusion on the sensing layer.^[^ ^82^ ^]^ The noble metal functionalization on hBN demonstrates a synergetic effect to make the device more gas‐selective and hydrophobic, showing its suitability in harsh environmental conditions.^[^ ^212^ ^]^ Feng et. al. reported Pt functionalized hBN nanosheets for detecting pollutant gases (H_2_ and CH_4_) under humid conditions.^[^ ^46^ ^]^ They focused on the hydrophobic nature of the hBN sensor to design a humidity‐tolerant selective sensor system. On the contrary, Chen et. al. designed a new strategy to enhance the sensitivity of the hBN to water molecules, by a simple supramolecular functionalization approach for breath monitoring.^[^ ^46^ ^]^ The noncovalent supermolecular functionalization provides the stability and lack of re‐aggregation of the material (Figure 8g). A wireless sensor system with excellent sensitivity (>1010 Ohms per %RH) and fast response (0.1 ms) was reported to demonstrate the applicability of hBN for humidity sensing applications. This PS1 functionalization in hBN sheets enhances the sensitivity toward water molecules and can be attributed to the sulfonic group of PS1 that increases the interaction of hBN sheets toward water molecules by hydrogen bonding (Figure 8h). They also designed a real‐time noninvasive breath monitoring system that can detect the smallest change in respiratory signals associated with day‐to‐day activities and flu symptoms (Figure 8i). Surface‐activated hBN material demonstrated a higher sensitivity toward gases such as CO_2_,^[^ ^213^ ^]^ NH_3_,^[^ ^214^ ^]^ and NO_2_,^[^ ^215^ ^]^ significantly higher than pristine hBN and most other pristine 2D materials.

The outstanding properties of g‐C_3_N_4_ make it an ideal candidate for photoactivated gas sensors, but lower conductivity compared to other primitive gas sensing materials like metal oxides inhibits the desirability of the material in gas sensors.^[^ ^216^, ^217^, ^218^ ^]^ Therefore, different approaches, dopant engineering, high‐temperature graphitization, and surface functionalization were adopted to improve the properties of g‐C_3_N_4_.^[^ ^67^, ^210^, ^211^, ^217^ ^]^ Amidst surface engineering, metal oxide was added in the g‐C_3_N_4_ to tune the properties like charge transportation and surface defects. In this scenario, CuO hollow polyhedral functionalized g‐C_3_N_4_ nanosheets marked room temperature remarkable diethylamine(DEA) gas sensing capabilities (Figure 8j).^[^ ^217^ ^]^ Among various sensors, 1.8%‐g‐C_3_N_4_‐CuO‐400 hollow polyhedral structures showed the highest selectivity and sensitivity to DEA, which was 21.6 times higher than pure CuO at RT (Figure 8j). Nanoparticle functionalization in a suitable ratio on the g‐C_3_N_4_ channel can be the best approach to facilitate more charge transfer between the material and lead to a huge free carrier and improved conductivity.^[^ ^217^, ^219^ ^]^ Nevertheless, noble metal nanoparticles significantly influence the g‐C_3_N_4_ adsorption capacity and interaction efficiency of the target gas analytes, resulting in enhanced gas sensing performance.^[^ ^50^, ^51^, ^216^, ^220^, ^221^, ^222^ ^]^ Midst noble metals, Ag is more prominent for gas sensing due to its high reactivity toward oxidation, high electrical conductivity, and highly mobile cation species.^[^ ^50^, ^51^, ^222^ ^]^ Moradi et. al. examined the effect of Ag concentration on the gas‐sensing performance of g‐C_3_N_4_ sheets.^[^ ^50^ ^]^ An optimum amount of Ag nanoparticles on the g‐C_3_N_4_ channel can show the maximum catalytic action and accelerated decomposition of toluene into CO_2_ and H_2_O. Consequently, a huge interaction between toluene gas resulted in a high response (61.3% for 50 ppm) at a decreased operating temperature of 200 °C. few ternary composites of g‐C_3_N_4_ with Au/TiO_2_,^[^ ^23^ ^]^ Ag/ZnO,^[^ ^51^ ^]^ Au/In_2_O_3_,^[^ ^220^ ^]^ and Ag/rGO^[^ ^222^ ^]^ were reported and claimed that the ternary heterostructure between participating materials modulates the transfer of free charge carriers from the g‐C_3_N_4_ channel and eventually huge carriers contribute to the surface sensing process outcomes in high response value. Furthermore, a verified survey claimed that the incorporation of noble metals (Ag,^[^ ^51^ ^]^ Au,^[^ ^23^, ^220^ ^]^) could be a worthwhile approach in photoactivated gas sensing applications. Photosensitized metal nanoparticles accelerated the transfer and separation of electron‐hole pairs due to the formation of Schottky barriers at the metal‐semiconductor junction.^[^ ^23^, ^51^ ^]^ The extraordinary localized surface plasmon resonance (LSPR) effect, size‐dependent visible light adsorption, and light‐assisted catalytic behavior of Au nanoparticles make it an ideal catalyst for photoactivated gas sensors.^[^ ^23^, ^220^ ^]^ Malik et. al. reported an Au−TiO_2_ loaded cubic gC_3_N_4_ nanohybrids for the sensing of volatile organic amines (VOAs), such as triethylamine (TEA), and photocatalytic degradation.^[^ ^23^ ^]^ Under the influence of the Au spillover effect, the sensor Au−TiO_2_@m‐CN (R_a_/R_g_ = 79.8) displays a 2.5 times better response. Such multifunctional materials could be the solution in the energy and environment domains in terms of expense and space viability. Zhen and coworkers implemented a similar idea and designed an H_2_S selective visible light‐activated electrochemical sensor using gC_3_N_4_ nanosheets with N_2_ pretreatment and probed with Cd.^[^ ^219^ ^]^ The Cd/N@g‐C_3_N_4_ nanocomposite showed a remarkably high response toward H_2_S under UV light illumination compared to the sensor without pretreatment of N_2_ plasma.

#### 4.3.4 Single Atom Catalyst

2D nitrides are not mature materials in the research field for various applications, still they show huge potential to incorporate single atoms in their coordination sites and result in significant drift in their electronic, physical, and chemical properties.^[^ ^223^, ^224^, ^225^, ^226^, ^227^, ^228^ ^]^ Among all, g‐C_3_N_4_ can facilitate ideal coordination sites for single‐atom catalysts within its so‐called nitrogen pot (filled with six lone pairs of electrons). To date, Considerable efforts have been devoted to the successful incorporation of transition metals such as Eu,^[^ ^223^ ^]^ Pt,^[^ ^224^, ^225^, ^226^ ^]^ Ru,^[^ ^227^ ^]^ and Co,^[^ ^228^ ^]^ in g‐C_3_N_4_ matrix have been reported. These single‐atom catalysts not only enhance the catalytic performance of the material but also improve stability and photo or electrochemical activities due to the strong chemical integration and presence of polarized charges.^[^ ^223^, ^224^ ^]^ Single‐atom catalyst‐based g‐C_3_N_4_ nanostructures have recently attracted immense attention for applications in selective gas sensors,^[^ ^223^, ^226^ ^]^ batteries,^[^ ^224^ ^]^ hydrogen production,^[^ ^225^ ^]^ photocatalysts,^[^ ^227^ ^]^ and water splitting.^[^ ^228^ ^]^ Mori et. al. reported single‐atom Eu within a framework of g‐C_3_N_4_ to drive a new research interest toward luminescent sensing material.^[^ ^223^ ^]^ It was evident that a single atom of Eu in the g‐C_3_N_4_ matrix acts as a potential interface for selective VOC sensing at room temperature. The findings shed light on how single‐atom Eu^3+^ complexes might be used in the exfoliated g‐C_3_N_4_ nanosheets to create potentially sensitive and selective luminous gas sensors.

#### 4.3.5 Black Phosphorus (BP)

Black phosphorus gained the wide attention of researchers in diverse fields of technology that benefit from its unique structural and physicochemical properties.^[^ ^68^ ^]^ 2D BP with high carrier mobility, band gap modulation, and honeycomb fold structures with larger adsorption sites can be extensively applied in gas sensors.^[^ ^52^, ^54^, ^229^, ^230^, ^231^, ^232^, ^233^, ^234^, ^235^, ^236^, ^237^, ^238^, ^239^ ^]^ However, poor stability and incomplete signal recovery in the pristine BP sheets demand further modification in the material.^[^ ^52^, ^68^ ^]^ In‐depth surface engineering in BP sheets is helpful for convenient sensing, on‐site, and in situ sensing for real‐time sensors.^[^ ^231^, ^232^, ^233^, ^234^, ^235^, ^236^, ^237^, ^238^, ^239^ ^]^ Recent progress demonstrated the effective employment of different surface engineering techniques in the pristine 2D BP to achieve good sensing properties.^[^ ^235^, ^236^, ^237^, ^238^, ^239^ ^]^ This part of the review article incorporates various surface engineering strategies in BP for gas sensing applications as discussed in Table 2.

##### Active site Engineering

**Figure 9:**
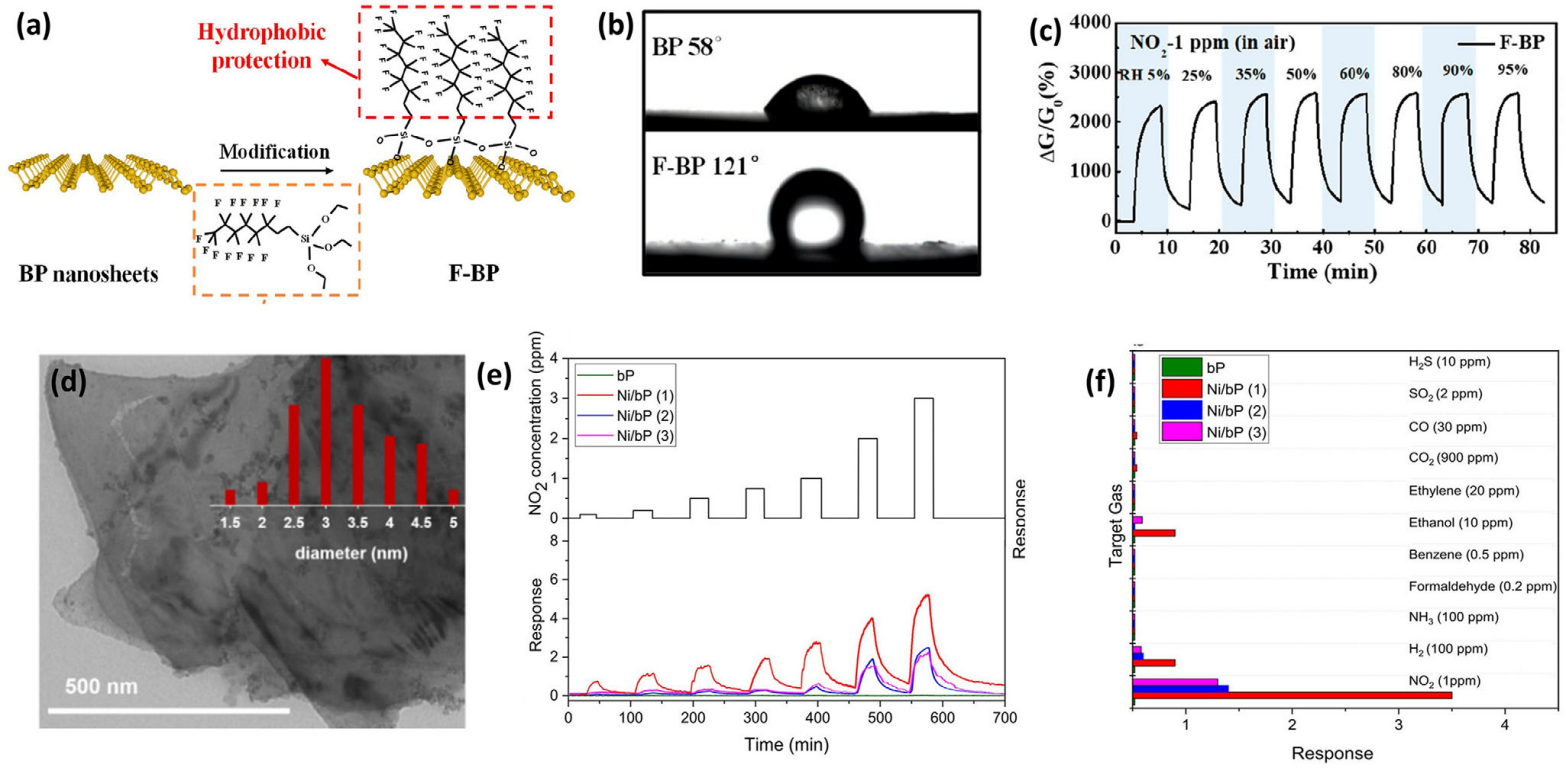
The surface engineering strategies in 2D BP gas sensors. a) Schematic view of the synthesis procedure of F‐BP. b) Water contact angle study of the pure BP and F‐BP films. c) Sensing response of the F‐BP sensor toward 1 ppm of NO_2_ at different relative humidity conditions. Reproduced with permission.^[^ ^21^ ^]^ Copyright 2021, American Chemical Society. d) TEM image of Ni functionalized BP film. e) Electrical characterization of different BP devices under dry conditions at different concentrations of NO_2_ at room temperature. f) Selectivity study of different BP devices for different interfering gases at RT. Reproduced with permission.^[^ ^56^ ^]^ Copyright 2021, American Chemical Society.

Black phosphorus‐based nanostructures have been extensively employed in gas sensors because of their high selectivity for paramagnetic gases such as NO_2_.^[^ ^68^ ^]^ Liquid phase exfoliated black phosphorus sheets have different layer spacing, increased oxygen incorporation, and phosphate ion defects, resulting in varied bandgap and surface states than the bulk material.^[^ ^54^, ^229^, ^230^ ^]^ Yasaei et. al. reported liquid exfoliated stacked nanoflakes of BP for highly sensitive and selective humidity detection based on the variation of ionic currents passing through the absorbed moist content.^[^ ^20^ ^]^ Evidently, an atomically thin layer of BP is more prone to chemical degradation due to humidity; perhaps robust configurations, such as multilayered films of BP, can withstand the degradation and potentially be utilized. The stability test confirmed their remark and showed a nearly unchanged response after prolonged exposures (up to 3 months) to humid conditions. The high adsorption energy toward NO_2_ and the higher surface‐to‐volume ratio of BP than other 2D materials, such as graphene, make it an ideal candidate for selective chemical sensors.^[^ ^52^, ^54^, ^231^ ^]^ Apart from this, the ambipolar behavior of BP sheets (both n‐type and p‐type doping) enables it to be designed as a field effect transistor (FET), which shows good noise performance, and a relatively high current on/off ratio that translates to enhanced gas response magnitude and detection limit.^[^ ^53^, ^232^ ^]^ Cui et. al. reported a detailed layer‐dependent NO_2_ sensing performance of a phosphorene FET gas sensor.^[^ ^232^ ^]^ The key focus was on theoretical evidence on layer‐dependent field effect mobility and bandgap of BP, and tuning the number of layers by the scotch tape method to report a potential chemical sensor. They claimed that 4.8 nm thick phosphorene nanosheets have demonstrated outstanding and reliable transistor properties with a sensing response of 190% for 20 parts per billion (ppb) NO_2_ at room temperature. Later, Abbas et. al discovered the high surface sensitivity of monolayered BP FET sensors toward NO_2_ even at a low concentration of 5 ppb.^[^ ^53^ ^]^ In monolayer BP, fast charge transfer is preferable, and due to this, conductance will be significantly increased while increasing the NO_2_ concentration. Defect engineering in black phosphorus (BP) gas sensors can also show remarkable potential for sensing applications. The susceptibility of BP toward NO_2_ gas was further improved by the researchers through defect‐enriched morphology and reported the lowest value of detection limit of 0.4 ppb, which was the lowest LOD for the NO_2_ detection so far.^[^ ^231^ ^]^ However, our group also reported a layered sensitive BP sensor for the selective detection of VOC.^[^ ^233^ ^]^ The high selectivity toward methanol was attributed to the impedance spectroscopy method, which is marked as a characteristic parameter for the selective detection of methanol. Few theoretical studies based on DFT analysis demonstrated that defect‐engineered phosphorene monolayer exhibits high sensitivity compared to the pristine toward toxic phosgene,^[^ ^234^ ^]^ SO_2_,^[^ ^235^ ^]^ and NO_2_^[^ ^237^ ^]^ gas detection. The pristine BP weakly interacts with toxic gases by van der Waals forces, characterized by small binding energies. Further controlled incorporation of multivalent vacancies and defects induces high adsorption energy for gas analytes, resulting in enhanced sensing resolution.^[^ ^234^, ^235^, ^236^ ^]^ Additionally, a suspended BP sensor fabricated by dry transfer has shown faster response and recovery with a fast NO_2_ gas desorption rate (two times faster) than the supported ones. The high sensitivity of the suspended 2D BP sensor was attributed to the larger adsorption area from all sides and neglected substrate effects.^[^ ^52^ ^]^ Xu and coworkers modeled the end groups of BP nanosheets via salinization treatment to control the chemical stability in oxidizing, humid environments by providing hydrophobic protection to the BP (**Figure** **9**a).^[^ ^21^ ^]^ The hydrophobic nature of modified BP sheets was optimized by water contact angle, where F‐BP material showed 121° of water contact angle (Figure 9b). This chemical passivation strategy of BP sheets resulted in small changes in the puckered phosphorus's electronic structure, leading to a hydrophobic NO_2_ sensing layer. The fluoroalkyl silane‐modified BP sensor showed excellent humidity‐independent sensing properties under a range of humidity from 5% to 95% RH (Figure 9c). These strong research findings highlight the crucial role of defects in BP in achieving excellent sensing performance and provide great promise for expanding the research on BP‐based gas sensors.

##### Doping Engineering

Doping in BP changes the surface structure to form covalent bonds and inhibits the degradation of the material.^[^ ^68^ ^]^ In gas sensing applications, doping black phosphorus (BP) with various elements such as alkali metals (Li, Na, K),^[^ ^238^ ^]^ Zn,^[^ ^239^ ^]^ co‐doped with benzyl viologen (BV), Au nanoparticles,^[^ ^240^ ^]^ and Mn,^[^ ^241^ ^]^ has demonstrated notable benefits. For instance, alkali metal‐doped BP showed improved NO_2_ detection sensitivity and selectivity.^[^ ^238^ ^]^ It was theoretically proven that the gas adsorption configuration of BP is different with different alkali metals. Differently, the Li‐BP sensing layer favored a vertical stabilization rather than a parallel configuration concerning the surface and resulted in increased efficiency for NO_2_ detection.^[^ ^238^ ^]^ However, hole charge carrier generation in BP due to doping could improve the conductivity, but poor recovery and reproducibility limitations of BP gas sensors remain a big challenge for the researchers. In addition, Zhao et. al. experimentally found that BP nanosheets sensitized with Zn‐doped α‐Fe_2_O_3_ nanoclusters are suitable for achieving excellent sensitivity, a quick reaction time, and a low detection limit for acetone detection.^[^ ^239^ ^]^ Later, a breathable PLA membrane was added to the sensing layer to overcome the deficiency in response due to humidity and oxidation. The covered layer showed a stable signal toward acetone vapor under different RH and excellent long‐term stability. Moreover, doped BP‐based sensors have not been explored much to improve the poor sensing performance of the material and require significant attention from researchers. These investigations demonstrated the applicability of doping engineering in black phosphorus materials to create stable, highly sensitive, and selective gas sensors for a range of uses.

##### Surface Functionalization

Surface functionalization is always a demanding route to engineer a 2D material according to the requirements for a specific application. Similarly, surface functionalization in BP material played a crucial role in enhancing the gas sensor performance in terms of sensitivity, selectivity, and long‐term reproducibility.^[^ ^68^ ^]^ Many research groups emphasized controlling and improving the electronic sensing characteristics by modulating the carrier concentration in the BP channel and with chemical sensitization by noble metals.^[^ ^19^, ^242^, ^243^ ^]^ The use of polydopamine (PDA) as a surface transformer on BP nanosheets has shown a significant increase in NO_2_ sensitivity, leading to improved gas‐sensing properties.^[^ ^244^ ^]^ A protective overlayer of noble metals is also an effective and convenient approach to preventing BP degradation. With the association of first‐principles studies, different experimental studies on the addition of several metal adatoms on phosphorene have been carried out, envisaging that the incorporation of transition metals on the surface of BP sheets preserves its structural integrity. Cho et. al. proposed that the Au‐ or Pt‐incorporated BP system protects the material from oxidation and humidity damage and exhibits high chemical and ambient stability, which is more difficult to achieve in other surface engineering routes.^[^ ^242^ ^]^ Valt et al. synthesized air‐stable Ni coordinated to BP sheets, as shown in the TEM image in Figure 9d, avoiding binding of oxygen molecules with the lone pair electrons of the phosphorus atoms, and Ni nanoparticle dimensions played a crucial role in the stability.^[^ ^56^ ^]^ The smaller Ni nanoparticles suppress the ambient degradation for up to 4 weeks and can be practically used for stable BP gas sensors under ambient conditions. The dynamic response curve of different BP and Ni/BP nanocomposites toward various NO_2_ concentrations shows stable baseline resistance and repeatability in sensing single even at lower concentration NO_2_ at RT (Figure 9e). In the selectivity study, Ni/bP nanocomposite (molar ratio P: Ni = 10) demonstrated the highest response and natural selectivity toward NO_2_ (Figure 9f). Additionally, Wang et al. tried to boost the natural selectivity of BP toward NO_2_ gas by developing an Ag‐enabled sensitization in BP nanosheets.^[^ ^243^ ^]^ The sensor detected trace NO_2_ (25 ppb) at room temperature, attributed to interfacial heterojunctions between BP nanosheets and Ag nanoparticles. This material showed improved stability when exposed to light, water, and oxygen, thereby successfully extending the lifetime of BP. Furthermore, higher sensitivity, better repeatability, and excellent selectivity for NO_2_ gas have been shown by functionalizing BP/WS_2_ nanosheets with Pt nanoparticles at low working temperatures for the first time.^[^ ^19^ ^]^ Metal oxides have been explicitly used in functionalizing 2D black phosphorus compared to other 2D nanomaterials. Metal oxides in the form of nanoparticles or quantum dots acting as a heteroatom catalyst possessing high resistance and charge modulation ability are more favorable for a better sensing response and signal stability.^[^ ^55^, ^245^, ^246^ ^]^ Liu et. al. successfully used Co_3_O_4_ nanoparticles to functionalize black phosphorus for the fastest NO_x_ sensing while using branched polyethyleneimine as a noncovalent assembly to introduce an air‐stable overlayer.^[^ ^55^ ^]^ The size‐controlled (in the range of 4−6 nm) Co_3_O_4_ NPs on the sensing channel were more prone to gaseous reactions and exhibited an ultrafast response in 0.67 s to NO_x_ at room temperature, with the lowest detection limit of 10 ppb than those of the pure BP materials. Nanosized Co_3_O_4_ possesses a huge density of defects, facilitating more chemically active sites and reactive centers for the interaction of target gas, resulting in an exceptional sensing response in the shortest reaction time. Out of the league, the addition of metal oxides such as TiO_2_,^[^ ^245^ ^]^ SnO,^[^ ^246^ ^]^ and SnO_2_,^[^ ^247^ ^]^ in BP sheets modulated its natural NO_2_ selectivity behavior and showed a higher response for NH_3_, and H_2_S gases, elevating the alternative approaches to designing selective BP gas sensors. Ren and coworkers designed a passivated sensor for high NH_3_ responsivity with less baseline drift and stronger long‐term stability.^[^ ^246^ ^]^ To examine long‐range BP selectivity, Liu et al. designed BP functionalized films using PEI/PEG via the one‐pot method.^[^ ^248^ ^]^ PEI/PEG‐BP film showed good sensitivity to CO_2_ with a wide detection range. The amino groups, meso‐macropores structure, and P–N heterojunction between BP and PEI resulted in a wide range of detection possibilities, from 250 000 to 200 ppm CO_2_.

## 5 Challenges and Future Prospects

This review provides a detailed analysis of how surface engineering can play a crucial role in modulating the properties of 2D materials and their sensing applications. The review begins with the introduction of emerging 2D materials in the gas sensing field, briefly introducing different 2D material classes, their possible synthesis methods, and explaining their key properties, such as highly chemically reactive, modulating electronic properties, and ultra‐high signal‐to‐noise ratio, and also discussing the gas sensing mechanism. This review also highlighted the major limitations, like poor selectivity and limited stability in 2D materials, that hinder their development as a practically advanced gas sensing technology. This review article comprises a comprehensive discussion of various surface engineering strategies, such as defect or active site manipulation, surface oxidation of reduction, heteroatom doping, surface functionalization, and single‐atom catalysts, followed by their influence on the properties of 2D materials and improvement in sensing performances. The review article also thoroughly explains the limitations and current progress of surface engineering strategies in a particular class of 2D material. These recent progresses in surface‐engineered 2D nanomaterial‐based gas sensors to enhance the sensing properties pave the way for the design of smarter and real‐time advanced gas sensing solutions.

Pristine 2D nanomaterials‐based gas sensors lack in performance due to low signal stability, baseline drift, susceptibility to cross‐interference, and poor long‐term signal stability, inhibiting them from being a good candidate for real‐time applications. Specifically, key challenges faced by 2D TMDs‐based gas sensors are their low sensitivity at lower detection limits and slow or poor recovery of signals. Surface functionalization of TMD materials with noble metals such as Au, Pd, Pt, and Ru is a good way to resist environmental oxidation and stabilize the material. Reports claimed that metal nanoparticle functionalized TMD sensors show enhanced responsivity, recovery rate, and long‐term stability. Advanced defect engineering techniques have been implemented to increase the interaction of 2D TMD materials with various gas analytes. The nanosized atomic vacancy defects in TMDs can be achieved through a sophisticated proton irradiation annealing technique with a defect precision of up to 10 nm, which effectively enhances the interaction of the gases at edge and surface defect sites, leading to enhanced sensitivity toward the target gas. Metallic MXene materials have a narrower bandgap, but appropriate etching routes enable huge surface reactive atoms to interact with oxygenated groups and show modulation in the electronic band structure. The tuned semiconducting bandgap provides more active adsorption sites, shifted work function, which directly influence the charge carrier transfer and gas sensitivity in the MXene sensors. Additionally, poor oxidation and signal stability are major drawbacks in MXene‐based gas sensing devices. Partial and controlled oxidation in MXenes, such as titanium carbide and vanadium carbide, directly form oxide counterparts like TiO_2_ and V_2_O_5_, respectively. The partial oxidation contributes to stability by passivating the abundant defects or functional sites and maintains the higher gas sensitivity by forming discrete metal/semiconductor Ti_3_C_2_T_x_/TiO_2_ junctions. Carbon nitrides, mainly hBN and g‐C_3_N_4_, suffer from less surface reactivity, which leads to poor sensing response toward the gas analytes. hBN is found to be a great support material for controlling the electronic properties. A study showcased that MoS_2_ on an hBN substrate showed a lowest detection limit of 6 ppb at room temperature, which was attributed to the lesser number of interfacial traps and disorders in the MoS_2_ on the hBN substrate sensor. On the other hand, defect engineering is the best way to introduce defective sites in 2D g‐C_3_N_4_ material. Substitution of C atoms with plenty of N atoms in the g‐C_3_N_4_ framework permits the material to construct numerous reactive sites. Lastly, the easy degradation of BP in light, water, and air environments due to the lone pair electron of phosphorus limits its applicability in real‐time gas sensing technology. A protective covering from noble metal functionalization can prevent the BP degradation and improve the stability of the material. Different experimental studies have suggested that the incorporation of transition metals on the surface of BP sheets preserves its structural integrity and maintains the reliability of gas sensing devices. Moreover, surface engineering strategies resolved may unsolved challenges in 2D material‐based gas sensors, resulting in promising gas‐sensing properties.

**Figure 10:**
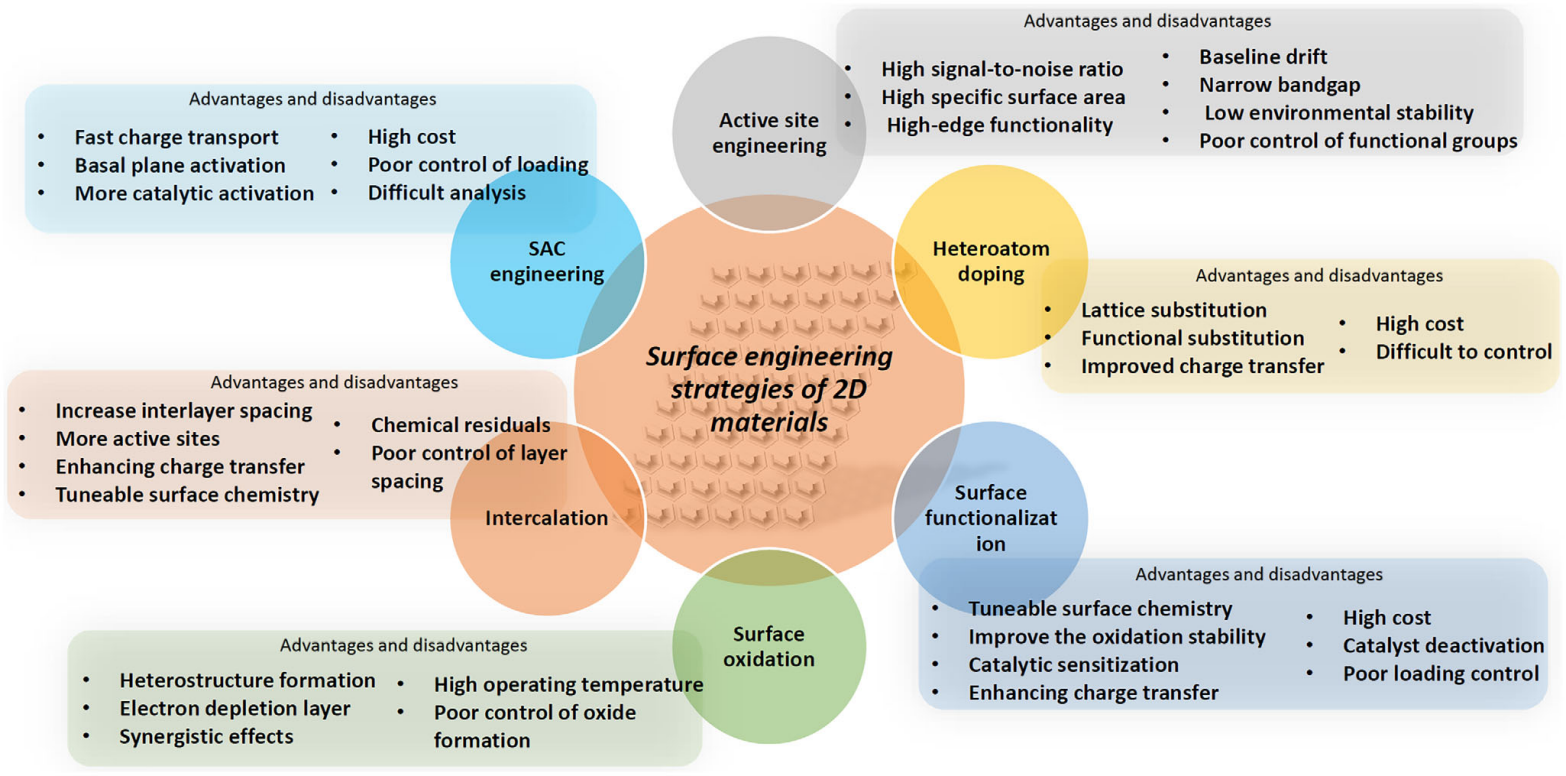
An analysis of different surface engineering approaches of 2D materials for gas sensing applications.

Although surface engineering routes marked a significant advancement in 2D materials gas sensing technology, several unresolved challenges must be addressed for more practical applications. **Figure** **10** demonstrates the pros and cons of surface engineering approaches in different 2D materials‐based gas sensors. Although improvements in various sensing parameters have been reported through surface modification techniques but it remains unclear which specific engineering approach is most effective for achieving particular performance enhancements. Additionally, the electronic and chemical mechanisms underlying these performance improvements are still not well understood and require more comprehensive investigation by researchers. Understanding the fundamental mechanisms driving the modified properties is essential to identifying the roles of participating materials and their interactions during the sensing process. 2D materials such as hBN, BP, and g‐C_3_N_4_ are less explored and offer extensive opportunities for modification to achieve superior sensing properties. Furthermore, there is a lack of research on the enhanced sensitivity and stability that result from surface engineering compared to pristine materials. Although some studies suggest that functionalization and doping with metal atoms or nanoparticles lead to improved detection limits and stability in 2D materials, the underlying reasons for these improvements have not been elucidated. Focused experimental and simulation studies are necessary to clarify these ambiguous explanations.

The future of surface‐engineered 2D materials appears highly promising due to their exceptional performance in gas‐sensing applications. While the majority of research in 2D material surface engineering has concentrated on a few basic surface modification strategies, such as heteroatom doping and metal nanoparticle functionalization, with widely recognized 2D materials like MoS_2_, WS_2_, Ti_3_C_2_T_x_, and g‐C_3_N_4_, there remain abundant opportunities for advancement in this field. Emerging and sophisticated surface engineering strategies, including quantum dot and single‐atom catalyst functionalization, atomic defect manipulation, and ion or nanoparticle intercalation in 2D sheets, hold the potential to enhance the compatibility of sensing materials with target gas analytes. For instance, the controlled introduction of noble metal single‐atom catalysts into photoactive g‐C_3_N_4_ material could significantly improve its photoactivated gas sensing properties and the performance of photovoltaic gas sensor devices. Additionally, other surface engineering methods, such as MXene doping with various catalytic heteroatoms and the functionalization of BP with surface capping agents, can further enhance the sensing capabilities of these materials. Combining 2D materials with appropriate surface engineering techniques and innovative device design represents a promising future alternative. For example, defect‐engineered atomically thin TMD and BP sheets are particularly significant in field‐effect transistor‐based gas sensor devices. New and fascinating SAC functionalization has shown excellent results in gas sensing, but is still immature and requires a lot more effort to optimize its preparation methods, gas sensing performance, and gas sensitization mechanisms. Furthermore, optimizing existing surface engineering strategies or employing novel ones to achieve precisely controlled sensing properties in 2D materials is crucial for their practical application in real‐time gas sensing. Strategies involving thermal, chemical, and light treatments show promise and offer feasibility in parameter control. Moreover, advanced surface modification techniques such as atomic layer deposition, ion beam exposure, and plasma treatment merit greater attention and could be explored further to effectively control distribution and parameters during synthesis. In summary, there is considerable scope for the development of new and advanced surface engineering techniques. Combining these with the most suitable 2D materials can achieve outstanding gas‐sensing outcomes in the future.

## Conflict of Interest

The authors declare no conflict of interest.

## References

[1] C. Yue , Z. Zhang , Z. Liu , Y. Mu , Z. Yang , D. Dastan , X. T. Yin , X. Ma , J. Alloys Compd. 2023, 981, 173742.

[2] S. H. Cho , J. M. Suh , B. Jeong , T. H. Lee , K. S. Choi , T. H. Eom , S. W. Choi , G. B. Nam , Y. J. Kim , H. W. Jang , Small 2024, 20, 2309744. 10.1002/smll.202309744 38507730

[3] J. Zhao , H. Wang , Y. Cai , J. Zhao , Z. Gao , Y. Y. Song , ACS Sens. 2024, 9, 1644. 38503265 10.1021/acssensors.4c00137

[4] H. Min , O. Kwon , J. Lee , E. Choi , J. Kim , N. Lee , K. Eum , K. H. Lee , D. W. Kim , W. Lee , Adv. Mater. 2024, 36, 2309041. 10.1002/adma.202309041 38041566

[5] S. Mishra , C. Patel , D. Pandey , S. Mukherjee , A. Raghuvanshi , Small 2024, 20, 2311448. 10.1002/smll.202311448 38326094

[6] N. Rohaizad , C. C. Mayorga‐Martinez , M. Fojtů , N. M. Latiff , M. Pumera , Chem. Soc. Rev. 2021, 50, 619. 33206730 10.1039/d0cs00150c

[7] C. C. Mayorga‐Martinez , A. Ambrosi , A. Y. S. Eng , Z. Sofer , M. Pumera , Adv. Funct. Mater. 2015, 25, 5611.

[8] F. J. Urbanos , S. Gullace , P. Samorì , ACS Nano 2022, 16, 11234. 35796589 10.1021/acsnano.2c04503

[9] Q. Zhao , W. Zhou , M. Zhang , Y. Wang , Z. Duan , C. Tan , B. Liu , F. Ouyang , Z. Yuan , H. Tai , Y. Jiang , Adv. Funct. Mater. 2022, 32, 2203528.

[10] Z. Cai , J. Chen , S. Xing , D. Zheng , L. Guo , J. Hazard. Mater. 2021, 416, 126195. 34492959 10.1016/j.jhazmat.2021.126195

[11] Q. Weng , X. Wang , X. Wang , Y. Bando , D. Golberg , Chem. Soc. Rev. 2016, 45, 3989. 27173728 10.1039/c5cs00869g

[12] G. Neri , Chemosensors 2015, 3, 1.

[13] L.‐X. Ou , M.‐Y. Liu , L.‐Y. Zhu , D. W. Zhang , H.‐L. Lu , Nano‐Micro Lett. 2022, 14, 206. 10.1007/s40820-022-00956-9 PMC9587164 36271065

[14] H. Long , Y. Li , K. Chai , W. Zeng , Sensors Actuators B Chem 2024, 417, 136183.

[15] F. Schedin , A. K. Geim , S. V. Morozov , E. W. Hill , P. Blake , M. I. Katsnelson , K. S. Novoselov , Nat. Mater. 2007, 6, 652. 17660825 10.1038/nmat1967

[16] Y. L. Chueh , S. Y. Tang , C. C. Yang , T. Y. Su , T. Y. Yang , S. C. Wu , Y. C. Hsu , Y. Z. Chen , T. N. Lin , J. L. Shen , H. N. Lin , P. W. Chiu , H. C. Kuo , ACS Nano 2020, 14, 12668. 32813498 10.1021/acsnano.0c01264

[17] E. Mitterreiter , B. Schuler , A. Micevic , D. Hernangómez‐Pérez , K. Barthelmi , K. A. Cochrane , J. Kiemle , F. Sigger , J. Klein , E. Wong , E. S. Barnard , K. Watanabe , T. Taniguchi , M. Lorke , F. Jahnke , J. J. Finley , A. M. Schwartzberg , D. Y. Qiu , S. Refaely‐Abramson , A. W. Holleitner , A. Weber‐Bargioni , C. Kastl , Nat. Commun. 2021, 12, 3822. 34158488 10.1038/s41467-021-24102-y PMC8219741

[18] B. Ozden , T. Zhang , M. Liu , A. Fest , D. A. Pearson , E. Khan , S. Uprety , J. E. Razon , J. Cherry , K. Fujisawa , H. Liu , N. Perea‐López , K. Wang , T. Isaacs‐Smith , M. Park , M. Terrones , ACS Nano 2023, 17, 25101. 38052014 10.1021/acsnano.3c07752

[19] X. Duan , D. Xu , W. Jia , B. Sun , R. Li , R. Yan , W. Zhao , Nanoscale 2023, 16, 2478. 10.1039/d3nr05424a 38226534

[20] P. Yasaei , A. Behranginia , T. Foroozan , M. Asadi , K. Kim , F. Khalili‐Araghi , A. Salehi‐Khojin , ACS Nano 2015, 9, 9898. 26401950 10.1021/acsnano.5b03325

[21] Y. Xu , X. Li , Y. Song , R. Zhang , W. Yuan , D. Xia , Q. Xue , ACS Appl. Mater. Interfaces 2021, 13, 50270. 34637261 10.1021/acsami.1c16776

[22] M. Sajjad , G. Morell , P. Feng , ACS Appl. Mater. Interfaces 2013, 5, 5051. 23662583 10.1021/am400871s

[23] R. Malik , V. K. Tomer , N. Joshi , T. Dankwort , L. Lin , L. Kienle , ACS Appl. Mater. Interfaces 2018, 10, 34087. 30198254 10.1021/acsami.8b08091

[24] H. Chen , H. Ma , C. Li , ACS Nano 2021, 15, 15502. 34597034 10.1021/acsnano.1c04423

[25] E. S. Muckley , M. Naguib , H. W. Wang , L. Vlcek , N. C. Osti , R. L. Sacci , X. Sang , R. R. Unocic , Y. Xie , M. Tyagi , E. Mamontov , K. L. Page , P. R. C. Kent , J. Nanda , I. N. Ivanov , ACS Nano 2017, 11, 11118. 29019645 10.1021/acsnano.7b05264

[26] D. Zhang , S. Yu , X. Wang , J. Huang , W. Pan , J. Zhang , B. E. Meteku , J. Zeng , J. Hazard. Mater. 2022, 423, 127160. 34537639 10.1016/j.jhazmat.2021.127160

[27] A. Ambrosi , C. K. Chua , A. Bonanni , M. Pumera , Chem. Rev. 2014, 114, 7150. 24895834 10.1021/cr500023c

[28] X. Chia , A. Y. S. Eng , A. Ambrosi , S. M. Tan , Pumera , Chem. Rev. 2015, 115, 11941. 26426313 10.1021/acs.chemrev.5b00287

[29] H. Li , J. Wu , Z. Yin , H. Zhang , Acc. Chem. Res. 2014, 47, 1067. 24697842 10.1021/ar4002312

[30] A. Ambrosi , M. Pumera , Chem. Soc. Rev. 2018, 47, 7213. 30132475 10.1039/c7cs00811b

[31] K. S. Kim , Y. Zhao , H. Jang , S. Y. Lee , J. M. Kim , K. S. Kim , J. H. Ahn , P. Kim , J. Y. Choi , B. H. Hong , Nature 2009, 457, 706. 19145232 10.1038/nature07719

[32] H. Li , G. Lu , Y. Wang , Z. Yin , C. Cong , Q. He , L. Wang , F. Ding , T. Yu , H. Zhang , Small 2013, 9, 1974. 23281258 10.1002/smll.201202919

[33] H. Schmidt , S. Wang , L. Chu , M. Toh , R. Kumar , W. Zhao , A. H. Castro Neto , J. Martin , S. Adam , B. Özyilmaz , G. Eda , Nano Lett. 2014, 14, 1909. 24640984 10.1021/nl4046922

[34] Y. Kim , I. Sohn , D. Shin , J. Yoo , S. Lee , H. Yoon , J. Park , S. Chung , H. Kim , Adv. Eng. Mater. 2024, 26, 2306063.

[35] R. Bhardwaj , A. Hazra , J. Mater. Chem. C. 2021, 9, 15735.

[36] Y. Gogotsi , B. Anasori , ACS Nano 2019, 13, 8491. 31454866 10.1021/acsnano.9b06394

[37] K. R. G. Lim , M. Shekhirev , B. C. Wyatt , B. Anasori , Y. Gogotsi , Z. W. Seh , Nat. Synth. 2022, 1, 601.

[38] M. Wu , M. He , Q. Hu , Q. Wu , G. Sun , L. Xie , Z. Zhang , Z. Zhu , A. Zhou , ACS Sens. 2019, 4, 2763. 31564092 10.1021/acssensors.9b01308

[39] H. Tai , Z. Duan , Z. He , X. Li , J. Xu , B. Liu , Y. Jiang , Sensors Actuators, B Chem. 2019, 298, 126874.

[40] H. Pazniak , A. S. Varezhnikov , D. A. Kolosov , I. A. Plugin , A. D.i Vito , O. E. Glukhova , P. M. Sheverdyaeva , M. Spasova , I. Kaikov , E. A. Kolesnikov , P. Moras , A. M. Bainyashev , M. A. Solomatin , I. Kiselev , U. Wiedwald , V. V. Sysoev , Adv. Mater. 2021, 33, 2104878. 34601739 10.1002/adma.202104878 PMC11468926

[41] K. Rathi , N. K. Arkoti , K. Pal , Adv. Mater. Interfaces 2022, 9, 2200415.

[42] Y. Bian , L. Li , H. Song , Y. Su , Y. Lv , Sensors Actuators, B Chem. 2021, 332, 129512.

[43] D. Lee , M. Chae , H. D. Kim , Sensors Actuators B Chem 2023, 401, 135063. 10.1016/j.snb.2022.133245 PMC9791791 36589904

[44] M. Chae , D. Lee , S. Kim , H. D. Kim , Sensors Actuators B Chem. 2023, 394, 134373.

[45] L. Li , C. Wei , H. Song , Y. Yang , Y. Xue , D. Deng , Y. Lv , Anal. Chem. 2019, 91, 13158. 31510739 10.1021/acs.analchem.9b03452

[46] L. Chen , K. Hu , M. Lu , Z. Chen , X. Chen , T. Zhou , X. Liu , W. Yin , C. Casiraghi , X. Song , Adv. Mater. 2024, 36, 2312621. 10.1002/adma.202312621 38168037

[47] T. V. K. Karthik , H. Martínez‐García , F. Ortiz‐Chi , C. G. Espinosa‐González , J. G. Torres‐Torres , A. G. Hernandez , S. Godavarthi , M. K. Kesarla , Diam. Relat. Mater. 2023, 133, 109736.

[48] K. S. Pasupuleti , D. J. Nam , N. H. Bak , M. Reddeppa , J. E. Oh , S. G. Kim , H. D. Cho , M. D. Kim , J. Mater. Chem. C. 2022, 10, 160.

[49] P. Srinivasan , S. Samanta , J. B. B. Rayappan , K. Kailasam , Sensors Actuators B Chem. 2021, 349, 130828.

[50] R. Moradi , R. Yousefi , Z. Adelpour , M. Sadeghi , J. Alloys Compd. 2023, 932, 167539.

[51] H. Li , Y. Sun , Q. Zhang , H. Yuan , C. Dong , S. Xu , M. Xu , Appl. Surf. Sci. 2023, 638, 158010.

[52] G. Lee , S. Kim , S. Jung , S. Jang , J. Kim , Sensors Actuators, B Chem. 2017, 250, 569.

[53] A. N. Abbas , B. Liu , L. Chen , Y. Ma , S. Cong , N. Aroonyadet , M. Köpf , T. Nilges , C. Zhou , ACS Nano 2015, 9, 5618. 25945545 10.1021/acsnano.5b01961

[54] T. Chen , Z. Cheng , Q. Tian , J. Wang , X. Yu , D. Ho , ACS Appl. Nano Mater. 2020, 3, 6440.

[55] Y. Liu , Y. Wang , M. Ikram , H. Lv , J. Chang , Z. Li , L. Ma , A. U. Rehman , G. Lu , J. Chen , K. Shi , ACS Sens. 2018, 3, 1576. 30019583 10.1021/acssensors.8b00397

[56] M. Valt , M. Caporali , B. Fabbri , A. Gaiardo , S. Krik , E. Iacob , L. Vanzetti , C. Malagù , M. Banchelli , C. D'Andrea , M. Serrano‐Ruiz , M. Vanni , M. Peruzzini , V. Guidi , ACS Appl. Mater. Interfaces 2021, 13, 44711. 34506713 10.1021/acsami.1c10763 PMC8461602

[57] R. K. Jha , N. Bhat , Adv. Mater. Interfaces 2020, 7, 1901992.

[58] R. R. Kumar , M. R. Habib , A. Khan , P. C. Chen , T. Murugesan , S. Gupta , A. Anbalagan , N. H. Tai , C. H. Lee , H. N. Lin , ACS Appl. Nano Mater. 2021, 4, 9459.

[59] Q. Sun , Z. Gong , Y. Zhang , J. Hao , S. Zheng , W. Lu , Y. Cui , L. Liu , Y. Wang , J. Hazard. Mater. 2021, 421, 126816. 34396968 10.1016/j.jhazmat.2021.126816

[60] Z. Qin , X. Song , J. Wang , X. Li , C. Wu , X. Wang , X. Yin , D. Zeng , Appl. Surf. Sci. 2021, 573, 151535.

[61] W. Yuan , K. Yang , H. Peng , F. Li , F. Yin , J. Mater. Chem. A 2018, 6, 18116.

[62] E. Lee , A. Vahidmohammadi , Y. S. Yoon , M. Beidaghi , D. J. Kim , ACS Sens. 2019, 4, 1603. 31244007 10.1021/acssensors.9b00303

[63] J. Song , J. Baek , J. Cho , T. Kim , M. Kim , H. S. Kim , J. Mun , S. W. Kang , Small Struct. 2023, 4, 2200392.

[64] J. Hu , J. Zhang , X. Liu , H. Zhang , X. X. Xue , Y. Zhang , Appl. Surf. Sci. 2023, 623, 157093.

[65] T. Kim , T. H. Lee , S. Y. Park , T. H. Eom , I. Cho , Y. Kim , C. Kim , S. A. Lee , M. J. Choi , J. M. Suh , I. S. Hwang , D. Lee , I. Park , H. W. Jang , ACS Nano 2023, 17, 4404. 36825770 10.1021/acsnano.2c09733

[66] Y. Shi , L. Ni , Z. Wang , M. Chen , L. Feng , Coord. Chem. Rev. 2024, 505, 215691.

[67] Y. Zhao , T. Wang , X. Li , Y. Fu , G. Zhao , X. Wang , Sensors Actuators A Phys. 2023, 355, 114313.

[68] D. An , X. Zhang , Z. Bi , W. Shan , H. Zhang , S. Xia , M. Qiu , Adv. Funct. Mater. 2021, 31, 2106484.

[69] K. S. Novoselov , A. K. Geim , S. V. Morozov , D. Jiang , Y. Zhang , S. V. Dubonos , I. V. Grigorieva , A. A. Firsov , Science 2004, 306, 666. 15499015 10.1126/science.1102896

[70] K. S. Subrahmanyam , L. S. Panchakarla , A. Govindaraj , C. N. R. Rao , J. Phys. Chem. C. 2009, 113, 4257.

[71] S. Manzeli , D. Ovchinnikov , D. Pasquier , O. V. Yazyev , A. Kis , Nat. Rev. Mater. 2017, 2, 17033.

[72] G. Eda , Y.‐Y. Lin , C. Mattevi , H. Yamaguchi , H.‐A. Chen , I.‐S. Chen , C.‐W. Chen , M. Chhowall , Phyl. Trans. R. Soc. London A. 2010, 22, 505.

[73] S. J. An , Y. H. Kim , C. Lee , D. Y. Park , M. S. Jeong , Sci. Rep. 2018, 8, 1. 30154571 10.1038/s41598-018-31374-w PMC6113326

[74] Y. H. Lee , X. Q. Zhang , W. Zhang , M. T. Chang , C. T.e Lin , K. D.i Chang , Y. C. Yu , J. T. W. Wang , C. S. Chang , L. J. Li , T. W. Lin , Adv. Mater. 2012, 24, 2320. 22467187 10.1002/adma.201104798

[75] S. Wang , X. Wang , J. H. Warner , W. E. T. Al , ACS Nano 2015, 9, 5246. 25895108 10.1021/acsnano.5b00655

[76] V. K. Singh , R. Pendurthi , J. R. Nasr , H. Mamgain , R. S. Tiwari , S. Das , A. Srivastava , ACS Appl. Mater. Interfaces 2020, 12, 16576. 32180391 10.1021/acsami.9b19820

[77] H. Miao , X. Hu , Q. Sun , Y. Hao , H. Wu , D. Zhang , J. Bai , E. Liu , J. Fan , X. Hou , Mater. Lett. 2016, 166, 121.

[78] C. J. Zhang , S. H. Park , O. Ronan , A. Harvey , A. Seral‐Ascaso , Z. Lin , N. McEvoy , C. S. Boland , N. C. Berner , G. S. Duesberg , P. Rozier , J. N. Coleman , V. Nicolosi , Small 2017, 13, 1701677. 10.1002/smll.201701677 28692755

[79] M. Naguib , O. Mashtalir , J. Carle , V. Presser , J. Lu , L. Hultman , Y. Gogotsi , M. W. Barsoum , ACS Nano 2012, 6, 1322. 22279971 10.1021/nn204153h

[80] P. Sutter , J. Lahiri , P. Albrecht , E. Sutter , ACS Nano 2011, 5, 7303. 21793550 10.1021/nn202141k

[81] H. Wang , X. Zhang , H. Liu , Z. Yin , J. Meng , J. Xia , X. M. Meng , J. Wu , J. You , Adv. Mater. 2015, 27, 8109. 26524600 10.1002/adma.201504042

[82] N. Goel , M. Kumar , J. Mater. Chem. C 2021, 9, 1537.

[83] A. Maity , X. Sui , H. Pu , K. J. Bottum , B. Jin , J. Chang , G. Zhou , G. Lu , J. Chen , Nanoscale 2020, 12, 1500. 31859311 10.1039/c9nr09354k

[84] S. Seo , H. U. Lee , S. C. Lee , Y. Kim , H. Kim , J. Bang , J. Won , Y. Kim , B. Park , J. Lee , Sci. Rep. 2016, 6, 23736. 27026070 10.1038/srep23736 PMC4812320

[85] Y. Yu , W. Yan , W. Gao , P. Li , X. Wang , S. Wu , W. Song , K. Ding , J. Mater. Chem. A 2017, 5, 17199.

[86] Y. Xu , X. Shi , Y. Zhang , H. Zhang , Q. Zhang , Z. Huang , X. Xu , J. Guo , H. Zhang , L. Sun , Z. Zeng , A. Pan , K. Zhang , Nat. Commun. 2020, 11, 1330. 32165616 10.1038/s41467-020-14902-z PMC7067838

[87] R. Arsat , M. Breedon , M. Shafiei , P. G. Spizziri , S. Gilje , R. B. Kaner , K. Kalantar‐zadeh , W. Wlodarski , Chem. Phys. Lett. 2009, 467, 344.

[88] M. Qazi , T. Vogt , G. Koley , Appl. Phys. Lett. 2007, 91, 233101.

[89] S. J. Choi , I. D. Kim , Korean Inst Metals Mater. 2018, 14, 221.

[90] J. Zhang , J. Ding , Y. Liu , C. Su , H. Yang , Y. Huang , B. Liu , Joule 2023, 7, 1700.

[91] L. Xue , Y. Ren , Y. Li , W. Xie , K. Chen , Y. Zou , L. Wu , Y. Deng , Small 2023, 19, 2302327. 10.1002/smll.202302327 37259638

[92] J. M. Suh , Y. S. Shim , K. C. Kwon , J. M. Jeon , T. H. Lee , M. Shokouhimehr , H. W. Jang , Electron. Mater. Lett. 2019, 15, 368.

[93] R. Bhardwaj , A. Hazra , Sensors Actuators B Chem. 2023, 401, 134967.

[94] X. Leng , Y. Wang , F. Wang , Adv. Mater. Interfaces 2019, 6, 1900010.

[95] L. Zhang , Y. Liang , L. Yu , H. Wang , M. Yin , Sensors Actuators B Chem 2022, 359, 131539.

[96] A. Kushwaha , N. Goel , Sensors Actuators B Chem 2023, 393, 134190.

[97] Q. Zhang , A. T. S. Wee , Q. Liang , X. Zhao , M. Liu , ACS Nano 2021, 15, 2165. 33449623 10.1021/acsnano.0c09666

[98] Y. Xia , C. Hu , S. Guo , L. Zhang , M. Wang , J. Peng , L. Xu , J. Wang , ACS Appl. Nano Mater. 2020, 3, 665.

[99] C. M. Lee , C. H. Jin , C. H. Ahn , H. K. Cho , J. H. Lim , S. M. Hwang , J. Joo , Phys. Status Solidi Appl. Mater. Sci. 2019, 216, 1800999.

[100] M. K. Rajbhar , S. De , G. Sanyal , A. Kumar , B. Chakraborty , S. Chatterjee , ACS Appl. Nano Mater. 2023, 6, 5284.

[101] R. Rao , H. Kim , N. Perea‐López , M. Terrones , B. Maruyama , Nanoscale 2021, 13, 11470. 34160535 10.1039/d1nr01483h

[102] B. Zhang , Y. Liu , T. Liang , T. Sakthivel , L. Yu , Z. Dai , ACS Appl. Nano Mater. 2020, 3, 4642.

[103] Q. Zhou , H. Song , T. Sun , L. Zhang , Y. Lv , Sensors Actuators B Chem. 2021, 2022, 353.

[104] N. Hou , Q. Sun , J. Yang , S. You , Y. Cheng , Q. Xu , W. Li , S. Xing , L. Zhang , J. Zhu , Q. Yang , Nano Res. 2020, 13, 1704.

[105] K. Zhao , X. Chang , J. Zhang , F. Yuan , X. Liu , ACS Sens. 2024, 9, 388. 38147687 10.1021/acssensors.3c02148

[106] S. P. Linto Sibi , M. Rajkumar , K. Govindharaj , J. Mobika , V. Nithya Priya , R. T. Rajendra Kumar , Anal. Chim. Acta. 2023, 1248, 340932. 36813461 10.1016/j.aca.2023.340932

[107] S. Y. Lee , G. Shim , J. Park , H. Seo , Phys. Chem. Chem. Phys. 2018, 20, 16932. 29682636 10.1039/c8cp00158h

[108] R. Wu , J. Hao , T. Wang , S. Zheng , Y. Wang , Inorg. Chem. Front. 2021, 8, 5006.

[109] C. Patel , V. K. Verma , S. Chaudhary , R. Bhardwaj , S. Mukherjee , ACS Appl. Nano Mater. 2024, 7, 4546.

[110] K. Wang , L. Lee , S. L. Loo , T. Y. Yang , C. T. Chen , T. W. Kuo , J. L. Chen , H. C. Kuo , Y. L. Chueh , ACS Appl. Nano Mater. 2023, 6, 5336.

[111] H. Yang , X. Li , Q. Wu , H. Su , C. Ma , X. Wang , C. Xie , D. Zeng , Sensors Actuators B Chem. 2023, 376, 133033.

[112] W. T. Koo , J. H. Cha , J. W. Jung , S. J. Choi , J. S. Jang , D. H. Kim , I. D. Kim , Adv. Funct. Mater. 2018, 28, 1802575.

[113] D. Burman , H. Raha , B. Manna , P. Pramanik , P. K. Guha , ACS Sens. 2021, 6, 3398. 34494827 10.1021/acssensors.1c01258

[114] T. Li , S. Yu , Q. Li , M. Chi , P. Li , New J. Chem. 2021, 45, 21423.

[115] J. Chang , C. Qin , Y. Zhang , L. Zhu , Y. Zhang , Y. Wang , J. Cao , Sensors Actuators B Chem. 2023, 395, 134511.

[116] A. Taufik , Y. Asakura , T. Hasegawa , H. Kato , M. Kakihana , S. Hirata , M. Inada , S. Yin , ACS Appl. Nano Mater. 2020, 3, 7835.

[117] S. Mobtakeri , S. Habashyani , Ö. Çoban , H. F. Budak , A. E. Kasapoğlu , E. Gür , Sensors Actuators B Chem. 2022, 381, 133485.

[118] V. Paolucci , J. De Santis , L. Lozzi , G. Giorgi , C. Cantalini , Sensors Actuators B Chem. 2021, 350, 130890.

[119] H. Tang , C. Gao , H. Yang , L. Sacco , R. Sokolovskij , 2D Mater. 2017,8, 045006.

[120] S. Rani , M. Kumar , P. Garg , R. Parmar , A. Kumar , Y. Singh , V. Baloria , U. Deshpande , V. N. Singh , ACS Appl. Mater. Interfaces 2022, 14, 15381. 35344324 10.1021/acsami.1c24679

[121] Y. Han , Y. Liu , C. Su , X. Chen , B. Li , W. Jiang , M. Zeng , N. Hu , Y. Su , Z. Zhou , Z. G. Zhu , Z. Yang , ACS Appl. Nano Mater. 2021, 4, 1626.

[122] S. Mobtakeri , S. Habashyani , E. Gür , ACS Appl. Mater. Interfaces 2022, 14, 25741. 35608898 10.1021/acsami.2c04804 PMC9185678

[123] X. Liu , J. Xu , Z. Cheng , J. Yang , Y. Li , ACS Appl. Nano Mater. 2022, 5, 12592.

[124] H. Yang , C. Zhu , Q. Wu , X. Li , H. Wang , J. Wan , C. Xie , D. Zeng , Appl. Surf. Sci. 2022, 601, 154213.

[125] Z. Zhang , M. Zhang , Z. Wu , S. Wang , J. Zhang , Phys. Status Solidi – Rapid Res. Lett. 2023, 17, 1.

[126] X. Li , W. Liu , B. Huang , H. Liu , X. Li , J. Mater. Chem. C 2020, 8, 15804.

[127] H. Park , J. Kim , S. Ahn , A. Mirzaei , J. Kim , C. Park , Sensors Actuators B. Chem. 2024, 427, 137167.

[128] S. Lee , Y. Kang , J. Lee , J. Kim , J. W. Shin , S. Sim , D. Go , E. Jo , S. Kye , J. Kim , J. An , Appl. Surf. Sci. 2021, 571, 151256.

[129] R. Bhardwaj , V. Selamneni , U. N. Thakur , P. Sahatiya , A. Hazra , New J. Chem. 2020, 44, 16613.

[130] P. Bharathi , S. Harish , M. Shimomura , S. Ponnusamy , M. Krishna Mohan , J. Archana , M. Navaneethan , Sensors Actuators B Chem. 2022, 360, 131600.

[131] N. Sakhuja , A. Gupta , R. Jha , N. Bhat , J. Alloys Compd. 2022, 899, 163166.

[132] A. Alagh , F. E. Annanouch , K. A.l Youssef , C. Bittencourt , F. Güell , P. R. Martínez‐Alanis , M. Reguant , E. Llobet , Sensors Actuators B Chem. 2022, 364, 131905.

[133] R. Yan , W. Zhao , X. Duan , T. Yu , W. Cui , W. Quan , Y. Chen , D. Xu , Microchem. J. 2024, 209, 112892.

[134] J. Zhao , S. Zhang , M. Xu , P. Xu , J. Wang , B. Gao , Materials Science & Engineering B. 2025, 312, 117882.

[135] W. Guo , K. Chen , S. Wang , H. Zhang , D. Wu , Sensors Actuators B. Chem. 2025, 433, 137490.

[136] X. Duan , W. Zhao , R. Yan , T. Yu , W. Quan , Y. Chen , D. Xu , Chem. Eng. J. 2025, 509, 161219.

[137] R. Gond , S. Barala , P. Shukla , G. Bassi , S. Kumar , M. Kumar , M. Kumar , B. R. G. Rawat , S. Barala , P. Shukla , G. Bassi , S. Kumar , M. Kumar , M. Kumar , B. Rawat , ACS Sens. 2025, 10, 3412. 40292931 10.1021/acssensors.4c03297

[138] J. Liu , Z. Hu , Y. Zhang , H. Y. Li , N. Gao , Z. Tian , L. Zhou , B. Zhang , J. Tang , J. Zhang , F. Yi , H. Liu , Nano‐Micro Lett. 2020, 12, 59. 10.1007/s40820-020-0394-6 PMC7770826 34138314

[139] J. H. Kim , I. Sakaguchi , S. Hishita , T. Ohsawa , T. T. Suzuki , N. Saito , Sensors Actuators B Chem. 2022, 382, 133501.

[140] D. Shin , I. Sohn , J. Kim , T. Nakazawa , S. Lee , H. Yoon , J. Yoo , J. Park , S. M. Chung , H. Kim , ACS Appl. Nano Mater. 2023, 6, 19327.

[141] Q. Guang , B. Huang , J. Yu , M. Bonyani , M. Moaddeli , M. Kanani , A. Mirzaei , H. W. Kim , S. S. Kim , X. Li , Sensors Actuators B Chem. 2023, 394, 134399.

[142] Z. Li , Y. Liao , Y. Liu , W. Zeng , Q. Zhou , Appl. Surf. Sci. 2022, 610, 155527.

[143] J. Chang , C. Qin , W. Guo , L. Zhu , Y. Zhang , Y. Wang , J. Cao , Sensors Actuators B Chem. 2023, 385, 133633.

[144] J. H. Kim , A. Mirzaei , I. Sakaguchi , S. Hishita , T. Ohsawa , T. T. Suzuki , S. Sub Kim , N. Saito , Appl. Surf. Sci. 2023, 641, 158478.

[145] W. Liu , D. Gu , X. Li , ACS Appl. Mater. Interfaces 2021, 13, 20336. 33900063 10.1021/acsami.1c02500

[146] L. Li , H. Su , L. Zhou , Z. Hu , T. Li , B. Chen , H. Y. Li , H. Liu , Chem. Eng. J. 2023, 472, 144796.

[147] B. Liu , Q. Zhu , Y. Pan , F. Huang , L. Tang , C. Liu , Z. Cheng , P. Wang , J. Ma , M. Ding , ACS Sens. 2022, 7, 1533. 35546283 10.1021/acssensors.2c00356

[148] D. Chen , Y. Li , S. Xiao , C. Yang , J. Zhou , B. Xiao , Appl. Surf. Sci. 2021, 579, 152141.

[149] F. Xia , J. Lao , R. Yu , X. Sang , J. Luo , Y. Li , J. Wu , Nanoscale 2019,11, 23330. 31793604 10.1039/c9nr07236e

[150] A. Junkaew , R. Arróyave , Phys. Chem. Chem. Phys. 2018, 20, 6073. 29457806 10.1039/c7cp08622a

[151] Q. Sun , J. Wang , X. Wang , J. Dai , X. Wang , H. Fan , Z. Wang , H. Li , X. Huang , W. Huang , Nanoscale 2020, 12, 16987. 32780062 10.1039/c9nr08350b

[152] Y. Yao , Y. Han , Z. Wang , Z. Li , Z. Zhu , Sensors Actuators B Chem. 2023, 402, 135078.

[153] T. Thomas , J. A. Ramos Ramón , V. Agarwal , A. Á. Méndez , J. A. A. Martinez , Y. Kumar , K. C. H. S. Sanal , Microporous Mesoporous Mater. 2022, 336, 111872.

[154] X. Li , Z. An , Y. Lu , J. Shan , H. Xing , G. Liu , Z. Shi , Y. He , Q. Chen , R. P. S. Han , D. Wang , J. Jiang , F. Zhang , Q. Liu , Adv. Mater. Technol. 2022, 7, 2100872.

[155] S. J. Kim , H. J. Koh , C. E. Ren , O. Kwon , K. Maleski , S. Y. Cho , B. Anasori , C. K. Kim , Y. K. Choi , J. Kim , Y. Gogotsi , H. T. Jung , ACS Nano 2018, 12, 986. 29368519 10.1021/acsnano.7b07460

[156] Y. Pei , X. Zhang , Z. Hui , J. Zhou , X. Huang , G. Sun , W. Huang , ACS Nano 2021, 15, 3996. 33705113 10.1021/acsnano.1c00248

[157] S. Kim , J. Lee , S. Doo , Y. C. Kang , C. M. Koo , S. J. Kim , ACS Appl. Nano Mater. 2021, 4, 14249.

[158] M. Wu , Y. An , R. Yang , Z. Tao , Q. Xia , Q. Hu , M. Li , K. Chen , Z. Zhang , Q. Huang , S. H. Ma , A. Zhou , ACS Appl. Nano Mater. 2021, 4, 6257.

[159] H. J. Koh , S. J. Kim , K. Maleski , S. Y. Cho , Y. J. Kim , C. W. Ahn , Y. Gogotsi , H. T. Jung , ACS Sens. 2019, 4, 1365. 31062965 10.1021/acssensors.9b00310

[160] J. Lee , Y. C. Kang , C. M. Koo , S. J. Kim , ACS Appl. Nano Mater. 2022, 5, 11997.

[161] Y. Zhang , Y. Jiang , Z. Duan , Q. Huang , Y. Wu , B. Liu , Q. Zhao , S. Wang , Z. Yuan , H. Tai , Sensors Actuators B Chem 2021, 344, 130150.

[162] Z. Yang , A. Liu , C. Wang , F. Liu , J. He , S. Li , J. Wang , R. You , X. Yan , P. Sun , Y. Duan , G. Lu , ACS Sens. 2019, 4, 1261. 30990023 10.1021/acssensors.9b00127

[163] J. L. Hart , K. Hantanasirisakul , A. C. Lang , B. Anasori , D. Pinto , Y. Pivak , J. T. van Omme , S. J. May , Y. Gogotsi , M. L. Taheri , Nat. Commun. 2019, 10, 522. 30705273 10.1038/s41467-018-08169-8 PMC6355901

[164] J. Choi , B. Chacon , H. Park , K. Hantanasirisakul , T. Kim , K. Shevchuk , J. Lee , H. Kang , S. Y. Cho , J. Kim , Y. Gogotsi , S. J. Kim , H. T. Jung , ACS Sens. 2022, 7, 2225. 35838305 10.1021/acssensors.2c00658

[165] Y. Zhou , Y. Wang , Y. Wang , H. Yu , R. Zhang , J. Li , Z. Zang , X. Li , ACS Appl. Mater. Interfaces 2021, 13, 56485. 34787994 10.1021/acsami.1c17429

[166] Y. Zhou , Y. Wang , Y. Wang , X. Li , Anal. Chem. 2020, 92, 16033. 33237743 10.1021/acs.analchem.0c03664

[167] S. N. Shuvo , A. M. Ulloa Gomez , A. Mishra , W. Y. Chen , A. M. Dongare , L. A. Stanciu , ACS Sens. 2020, 5, 2915. 32786375 10.1021/acssensors.0c01287

[168] M. Sanna , K. A. Novčić , S. Ng , M. Černý , M. Pumera , J. Mater. Chem. A. 2023, 11, 3080.

[169] S. M. Majhi , A. Ali , Y. E. Greish , H. F. El‐Maghraby , S. T. Mahmoud , Sci. Rep. 2023, 13, 3114. 36813817 10.1038/s41598-023-30002-6 PMC9947003

[170] H. Pazniak , I. A. Plugin , M. J. Loes , T. M. Inerbaev , I. N. Burmistrov , M. Gorshenkov , J. Polcak , A. S. Varezhnikov , M. Sommer , D. V. Kuznetsov , M. Bruns , F. S. Fedorov , N. S. Vorobeva , A. Sinitskii , V. V. Sysoev , ACS Appl. Nano Mater. 2020, 3, 3195.

[171] F. Cao , Y. Zhang , H. Wang , K. Khan , A. K. Tareen , W. Qian , H. Zhang , H. Ågren , Advanced Materials. 2022, 34, 2107554. 10.1002/adma.202107554 34816509

[172] S. Liu , M. Wang , G. Liu , N. Wan , C. Ge , S. Hussain , H. Meng , M. Wang , G. Qiao , Appl. Surf. Sci. 2021, 567, 150747.

[173] D. Kuang , L. Wang , X. Guo , Y. She , B. Du , C. Liang , W. Qu , X. Sun , Z. Wu , W. Hu , Y. He , J. Hazard. Mater. 2020, 416, 126171. 10.1016/j.jhazmat.2021.126171 34492947

[174] W. Y. Chen , S. N. Lai , C. C. Yen , X. Jiang , D. Peroulis , L. A. Stanciu , ACS Nano 2020, 14, 11490. 32857499 10.1021/acsnano.0c03896

[175] A. N. Kumar , K. Pal , Mater. Adv. 2022, 3, 5151.

[176] N. Li , Y. Jiang , Y. Xiao , B. Meng , C. Xing , H. Zhang , Z. Peng , Nanoscale 2019, 11, 21522. 31686085 10.1039/c9nr06751e

[177] M. S. Nam , J. Y. Kim , A. Mirzaei , M. H. Lee , H. W. Kim , S. S. Kim , Sensors Actuators B Chem. 2023, 403, 135112.

[178] G. Feng , S. Wang , S. Wang , P. Wang , C. Wang , Y. Song , J. Xiao , C. Song , Sensors Actuators B Chem. 2024, 400, 134852.

[179] Z. Wang , F. Yan , Z. Yu , H. Cao , Z. Ma , Z. N. YeErKenTai , Z. Li , Y. Han , Z. Zhu , ACS Sens. 2024, 9, 1447. 38412069 10.1021/acssensors.3c02558

[180] Z. Zhu , C. Liu , F. Jiang , J. Liu , X. Ma , P. Liu , J. Xu , L. Wang , R. Huang , J. Hazard. Mater. 2020, 399, 123054. 32526430 10.1016/j.jhazmat.2020.123054

[181] S. Zou , J. Gao , L. Liu , Z. Lin , P. Fu , S. Wang , Z. Chen , J. Alloys Compd. 2020, 817, 152785.

[182] Z. Wang , K. Yu , Y. Feng , R. Qi , J. Ren , Z. Zhu , Appl. Surf. Sci. 2019, 496, 143729.

[183] K. S. Ranjith , S. Sonwal , A. Mohammadi , G. Seeta , R. Raju , Y. S. Huh , Y. Han , J. Mater. Chem. A. 2025, 13, 2950.

[184] P. Shi , Z. Wang , F. Shi , Mater. Res. Express 2025, 12, 015901.

[185] J. Liang , Y. Han , H. Chen , Y. Zhang , X. Gao , J. Alloys Compd. 2024, 1010, 177798.

[186] B. Li , J. Liu , Z. Lin , C. Mai , A. Cao , D. Yao , Z. Chen , F. Qu , Mater. Lett. 2024, 383, 137979.

[187] S. Kotteeswaran , S. Kondee , W. Pon‐on , W. H. Al‐qahtani , Microchem. J. 2025, 212, 113291.

[188] B. Zong , Q. Xu , S. Mao , ACS Sens. 2022, 7, 1874. 35820060 10.1021/acssensors.2c00475

[189] T. Chu , C. Rong , L. Zhou , X. Mao , B. Zhang , F. Xuan , Adv. Mater. 2023, 35, 2206783. 10.1002/adma.202206783 36106690

[190] W. Chen , P. Li , J. Yu , P. Cui , X. Yu , W. Song , C. Cao , Nano Res. 2022, 15, 9544.

[191] H. Wu , J. Yu , G. Yao , Z. Li , W. Zou , X. Li , H. Zhu , Z. Huang , Z. Tang , Sensors Actuators B Chem. 2022, 369, 132195.

[192] R. Decker , Y. Wang , V. W. Brar , W. Regan , H.‐Z. Tsai , Q. Wu , W. Gannett , A. Zettl , M. F. Crommie , Nano Lett. 2011, 11, 2291. 21553853 10.1021/nl2005115

[193] T. Ayari , C. Bishop , M. B. Jordan , S. Sundaram , X. Li , S. Alam , Y. Elgmili , G. Patriarche , P. L. Voss , J. P. Salvestrini , A. Ougazzaden , Sci. Rep. 2017, 7, 15212. 29123115 10.1038/s41598-017-15065-6 PMC5680310

[194] A. Harju , Nano Lett. 2013, 13, 3199. 23786613 10.1021/nl401265f

[195] A. R. Cadore , E. Mania , A. B. Alencar , N. P. Rezende , S. de Oliveira , K. Watanabe , T. Taniguchi , H. Chacham , L. C. Campos , R. G. Lacerda , Sensors Actuators, B Chem. 2018, 266, 438.

[196] Y. Liu , S. Li , S. Meng , S. Xiao , H. Song , K. Du , Sensors Actuators B Chem. 2023, 396, 134558.

[197] A. Kotbi , M. Benyoussef , E. M. Ressami , M. Lejeune , B. Lakssir , M. Jouiad , Chemosensors 2022, 10, 470.

[198] K. S. Pasupuleti , D. Vidyasagar , L. N. Ambadi , N. Bak , S. G. Kim , M. D. Kim , Sensors Actuators B Chem. 2023, 394, 134471.

[199] J. Yan , M. T. F. Rodrigues , Z. Song , H. Li , H. Xu , H. Liu , J. Wu , Y. Xu , Y. Song , Y. Liu , P. Yu , W. Yang , R. Vajtai , H. Li , S. Yuan , P. M. Ajayan , Adv. Funct. Mater. 2017, 27, 1700653.

[200] F. Sun , Z. Hao , G. Liu , C. Wu , S. Lu , S. Huang , C. Liu , Q. Hong , X. Chen , D. Cai , J. Kang , Nanoscale 2018, 10, 4361. 29446428 10.1039/c7nr08035b

[201] B. A. Kalwar , W. Fangzong , A. M. Soomro , M. R. Naich , M. H. Saeed , I. Ahmed , RSC Adv. 2022, 12, 34185. 36545633 10.1039/d2ra06307g PMC9709776

[202] M. T. Ahmed , S. Hasan , S. Islam , F. Ahmed , Appl. Surf. Sci. 2023, 623, 157083.

[203] K. Liu , X. Zhu , B. Lin , Z. Lu , G. Zhang , Phys. E Low‐Dimensional Syst. Nanostructures 2020, 135, 114977.

[204] Y. Yang , Y. Xie , J. Liu , H. Wang , X. Li , T. Zhou , F. Sun , Z. Feng , X. Wang , F. Jia , Sensors Actuators B. Chem. 2024, 428, 137251.

[205] A. Govind , P. Bharathi , G. Mathankumar , M. K. Mohan , J. Archana , S. Harish , M. Navaneethan , Diam. Relat. Mater. 2022, 128, 109205.

[206] P. K. Basivi , K. S. Pasupuleti , D. Gelija , M. D. Kim , V. R. Pasupuleti , C. W. Kim , New J. Chem. 2022, 46, 19254.

[207] K. S. Pasupuleti , S. S. Chougule , D. Vidyasagar , N. Bak , N. Jung , Y.‐H. Kim , J. Lee , S.‐G. Kim , M. Kim , Nano Res. 2023, 16, 7682.

[208] S. Sethuraman , A. Marimuthu , R. Kattamuthu , G. Karuppasamy , Appl. Surf. Sci. 2021, 561, 150077.

[209] K. S. Pasupuleti , S. Ghosh , N. Jayababu , C. J. Kang , H. D. Cho , S. G. Kim , M. D. Kim , Sensors Actuators B Chem. 2022, 378, 133140.

[210] W. Pi , X. Chen , M. Humayun , Y. Yuan , W. Dong , G. Zhang , B. Chen , Q. Fu , Z. Lu , H. Li , Z. Tang , W. Luo , ACS Appl. Mater. Interfaces 2023, 15, 14979. 36894512 10.1021/acsami.3c00213

[211] J. Gao , Y. Yang , F. Li , D. Li , H. Yu , X. Dong , T. Wang , Sensors Actuators B Chem. 2024, 417, 136072.

[212] P. X. Feng , E. Chavez , C. Malca , Chemosensors 2018, 6, 49.

[213] N. J. Bareza , B. Paulillo , T. M. Slipchenko , M. Autore , I. Dolado , S. Liu , J. H. Edgar , S. Vélez , L. Martín‐Moreno , R. Hillenbrand , V. Pruneri , ACS Photonics 2022, 9, 34.

[214] B. J. Matsoso , C. Garcia‐Martinez , T. H. Mongwe , B. Toury , J. P. M. Serbena , C. Journet , JPhys Mater 2021, 4, 044007.

[215] W. Tian , H. Zhang , Y. Zhang , Y. Wang , J. Cao , Adv. Powder Technol. 2021, 32, 3801.

[216] A. Ibrahim , U. B. Memon , S. P. Duttagupta , R. S. Raman , A. Sarkar , G. Pendharkar , S. S. V. Tatiparti , Int. J. Hydrogen Energy 2021, 46, 23962.

[217] A. Hussain , M. Y. Suleiman , H. Liu , S. Xia , T. Eticha , Y. Guan , W. Chen , G. Xu , Anal. Chem. 2024, 96, 8965. 38764427 10.1021/acs.analchem.3c05968

[218] N. Meghana , V. Zimba , J. Nayak , Ceram. Int. 2025, 51, 6233.

[219] X. Chen , W. Zhang , L. Zhang , L. Feng , C. Zhang , J. Jiang , H. Wang , ACS Appl. Mater. Interfaces 2021, 13, 2052. 33347275 10.1021/acsami.0c19572

[220] C. Han , X. Li , J. Liu , H. Dong , W. Cheng , Y. Liu , J. Xin , X. Li , C. Shao , Y. Liu , Sensors Actuators B Chem 2022, 371, 132448.

[221] A. Nasri , B. Jaleh , M. Daneshnazar , R. S. Varma , Biosensors 2023, 13, 315. 36979527 10.3390/bios13030315 PMC10046684

[222] R. Moradi , Z. Adelpour , M. Sadeghi , R. Yousefi , Adv. Powder Technol. 2023, 34, 104170.

[223] K. Mori , H. Yamashita , T. Murakami , ACS Appl. Nano Mater. 2020, 3, 10209.

[224] W. Zhao , J. Wang , R. Yin , B. Li , X. Huang , L. Zhao , L. Qian , J. Colloid Interface Sci. 2020, 564, 28. 31896425 10.1016/j.jcis.2019.12.102

[225] Y. Zhu , T. Wang , T. Xu , Y. Li , C. Wang , Appl. Surf. Sci. 2018, 464, 36.

[226] S. Luo , Q. Zhou , W. Xue , N. Liao , Vacuum 2022, 200, 111014.

[227] C. Li , X. Dong , Y. Zhang , J. Hu , J. Yuan , G. Li , D. Chen , Y. Li , Appl. Surf. Sci. 2022, 596, 153471.

[228] F. Chen , P. Jiao , C. Zhao , Catal. Letters 2024, 154, 2579.

[229] D. Han , X. Han , X. Zhang , W. Wang , D. Li , H. Li , S. Sang , Sensors Actuators B Chem. 2022, 367, 132038.

[230] G. He , T. Dong , Z. Yang , P. Ohlckers , Chem. Mater. 2019, 31, 9917.

[231] D. Han , X. Han , L. Liu , D. Li , Y. Liu , Z. Liu , D. Liu , Y. Chen , K. Zhuo , S. Sang , ACS Appl. Mater. Interfaces 2022, 14, 13942. 35275490 10.1021/acsami.2c00407

[232] S. Cui , H. Pu , S. A. Wells , Z. Wen , S. Mao , J. Chang , M. C. Hersam , J. Chen , Nat. Commun. 2015, 6, 8632. 26486604 10.1038/ncomms9632 PMC4639804

[233] C. C. Mayorga‐Martinez , Z. Sofer , M. Pumera , Angew. Chemie – Int. Ed. 2015, 54, 14317. 10.1002/anie.201505015 26403872

[234] M. Ghambarian , Z. Azizi , M. Ghashghaee , Phys. Chem. Chem. Phys. 2020, 22, 9677. 32329502 10.1039/d0cp00427h

[235] T. Kaewmaraya , L. Ngamwongwan , P. Moontragoon , A. Karton , T. Hussain , J. Phys. Chem. C. 2018, 122, 20186.

[236] P. V. Aaryashree Shinde , A. Kumar , D. J. Late , C. S. Rout , J. Mater. Chem. C. 2021, 9, 3773.

[237] X. Liang , Z. Wu , Z. Zhao , M. Xu , Y. Liu , G. Tai , Chem. Eng. J. 2025, 506, 159679.

[238] A. Marjani , M. Ghambarian , M. Ghashghaee , Sci. Rep. 2021, 11, 842. 33436873 10.1038/s41598-020-80343-9 PMC7804848

[239] H. Zhao , J. Li , X. She , Y. Chen , Y. Wang , C. Zou , Y. Zhou , Sensors Actuators B Chem. 2023, 395, 134496.

[240] J. Li , Y. Zhou , Y. Wang , S. Zhou , R. Zhang , Y. Wang , Z. Zang , J. Electrochem. Soc. 2022, 169, 017513.

[241] M. Ghadiri , M. Ghashghaee , M. Ghambarian , Phys. Chem. Chem. Phys. 2020, 22, 15549. 32608400 10.1039/d0cp02013c

[242] S. Y. Cho , H. J. Koh , H. W. Yoo , H. T. Jung , Chem. Mater. 2017, 29, 7197.

[243] Y. Wang , Y. Zhou , J. Li , R. Zhang , H. Zhao , Y. Wang , J. Hazard. Mater. 2022, 435, 129086. 35650733 10.1016/j.jhazmat.2022.129086

[244] Y. Xu , X. Xie , R. Zhang , W. Yuan , Sensors Actuators B Chem. 2022, 372, 132670.

[245] Y. Wang , Y. Zhou , Y. Wang , R. Zhang , J. Li , X. Li , Z. Zang , Sensors Actuators B Chem. 2021, 349, 130770.

[246] H. Ren , Y. Zhou , Y. Wang , Y. Ou , C. Gao , Y. Guo , Sensors Actuators B Chem. 2022, 365, 131910.

[247] Y. Wang , Z. Hu , J. Li , H. Zhao , R. Zhang , Y. Ou , L. Xie , J. Yang , C. Zou , Y. Zhou , ACS Appl. Nano Mater. 2023, 6, 4034.

[248] L. Liu , S. Sang , D. Han , Z. Liu , X. Han , D. Li , Y. Chen , D. Liu , X. Liu , K. Yang , Y. Cheng , Sensors Actuators B Chem. 2022, 369, 132303.

